# LC3-dependent intercellular transfer of phosphorylated STAT1/2 elicits CXCL9^+^ macrophages and enhances radiation-induced antitumor immunity

**DOI:** 10.1172/JCI195279

**Published:** 2025-12-01

**Authors:** Jun-Yan Li, Ying-Qing Li, Jia-Hao Dai, Sha Gong, Qing-Mei He, Jie-Wen Bai, Sai-Wei Huang, Ying-Qi Lu, Yu-Fei Duan, Sen-Yu Feng, Xi-Rong Tan, Xiao-Yu Liang, Jun Ma, Rui Guo, Na Liu

**Affiliations:** 1 State Key Laboratory of Oncology in South China, Guangdong Key Laboratory of Nasopharyngeal Carcinoma Diagnosis and Therapy,; 2 Department of Pathology,; 3 Department of Outpatient,; 4 Department of Experimental Research, and; 5 Department of Radiation Oncology, Sun Yat-sen University Cancer Center, Guangzhou, China.

**Keywords:** Cell biology, Immunology, Oncology, Head and neck cancer, Innate immunity

## Abstract

The efficacy of anticancer treatments, including radiotherapy, depends on the activation of type I IFN signaling. However, its regulatory networks and mechanisms remain to be elucidated. Here, we report that tumor cell–intrinsic type I IFN signaling can be transferred to macrophages via secretory autophagy, inducing CXCL9^hi^ macrophages and enhancing CD8^+^ T cell–mediated antitumor immunity. Mechanistically, K63-linked ubiquitination at the K167 site of phosphorylated STAT2 (p-STAT2) facilitates its binding to LC3B, promoting the loading of p-STAT1 and p-STAT2 into extracellular vesicles and intercellular transference from tumor cells to macrophages, which, however, is suppressed by USP5-mediated STAT2 deubiquitination. Genetic depletion or pharmacological inhibition of USP5 promotes autophagy-dependent unconventional protein secretion of p-STAT1 and p-STAT2, leading to the induction of CXCL9^+^ macrophages. This process promotes the expression of T cell chemokines and upregulates the antigen presentation machinery, thereby enhancing radiation-induced CD8^+^ T cell antitumor immunity and radiotherapy efficacy. Our findings reveal a critical role of USP5 in type I IFN–induced antitumor immunity, providing potential targets for improving the efficacy of radiotherapy.

## Abstract 2

Inhibition of USP5 facilitates LC3-dependent intercellular transfer of P-STAT1/2 between tumor cells and macrophages, which thus elicits CXCL9^+^ macrophages and enhances radiation-induced antitumor immunity.

## Graphical abstract

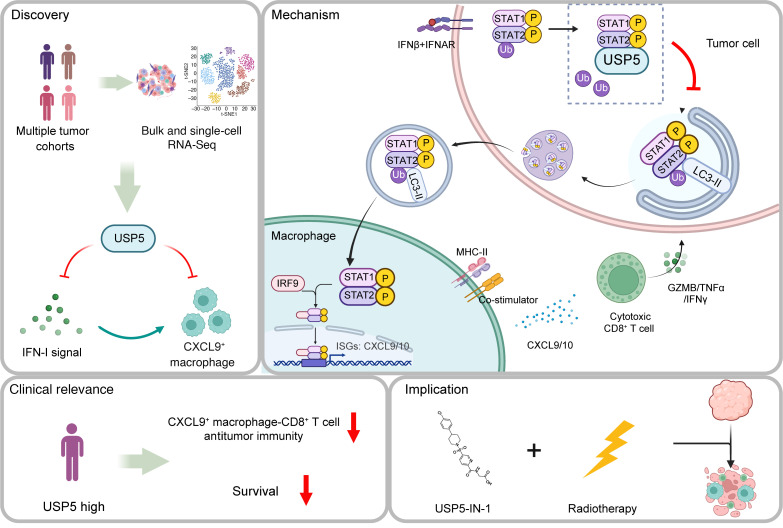

## Introduction

The efficacy of anticancer treatments, including radiotherapy, depends on the activation of intratumoral type I IFN (IFN-I) signaling (1, 2). While the importance of DNA sensing by the cGAS/STING pathway in treatment-induced IFN-I production has been well established (2–4), the mechanisms by which IFN-I signaling reprograms cell populations in the tumor microenvironment (TME) warrant further investigation. Macrophages are abundant, functionally active, and decisive cells in the TME (5, 6). Previous studies have summarized that IFN-I can inhibit the differentiation of tumor-supportive macrophages and promote M1 polarization and tumoricidal activity (7); however, recent research has emphasized the crucial role of macrophage subsets in antitumor immunity and treatment efficacy, especially in different tumor contexts (8, 9). To date, it remains unclear whether IFN-I signaling promotes antitumor immune response and enhances therapeutic efficacy by regulating a specific macrophage subset.

Intercellular communication plays critical roles in the induction and regulation of specific cell subsets within the TME. Secretory autophagy represents a form of unconventional secretion wherein autophagosomes, instead of fusing with lysosomes for the degradation of cellular components, fuse with the plasma membrane to release their contents extracellularly (10, 11). Secretory autophagy couples the autophagy machinery to the secretion of cellular contents via extracellular vesicles (EVs) and thus provides information to neighboring cells (12). Recently, it was reported that secretory autophagy can transport various substances, including cytokines such as IL-1β and HMGB1, proteins like α-synuclein, and organelles such as mitochondria, into the extracellular milieu (13–15). Consequently, secretory autophagy exerts pivotal functions in physiological or pathological processes, such as neurodevelopment, neurodegeneration, and myocardial homeostasis (16–18). In contrast, the role and mechanism of secretory autophagy in immunomodulation and the TME remain largely unknown.

Here, we demonstrate that the secretory autophagy of phosphorylated STAT1 and -2 (p-STAT1/2) in tumor cells elicits CXCL9^hi^ macrophages, thereby enhancing CD8^+^ T cell–mediated antitumor immunity. However, this process is suppressed by the tumor cell–intrinsic deubiquitinase USP5. Depletion of USP5 permits the transfer of p-STAT1/2 from tumor cells to macrophages in an LC3- and autophagy-dependent manner, conferring an immunostimulatory phenotype of macrophages. Furthermore, we provide proof-of-concept evidence demonstrating that the inhibition of USP5 with genetic and pharmacological interventions can suppress tumor progression and sensitize tumors to radiotherapy. Collectively, our study provides biological insight into IFN-I–mediated immune activation and may have significant therapeutic implications for improving the efficacy of radiotherapy-based anticancer treatments.

## Results

### IFN-I activates CXCL9^hi^ macrophage–mediated antitumor immunity.

**Figure 1:**
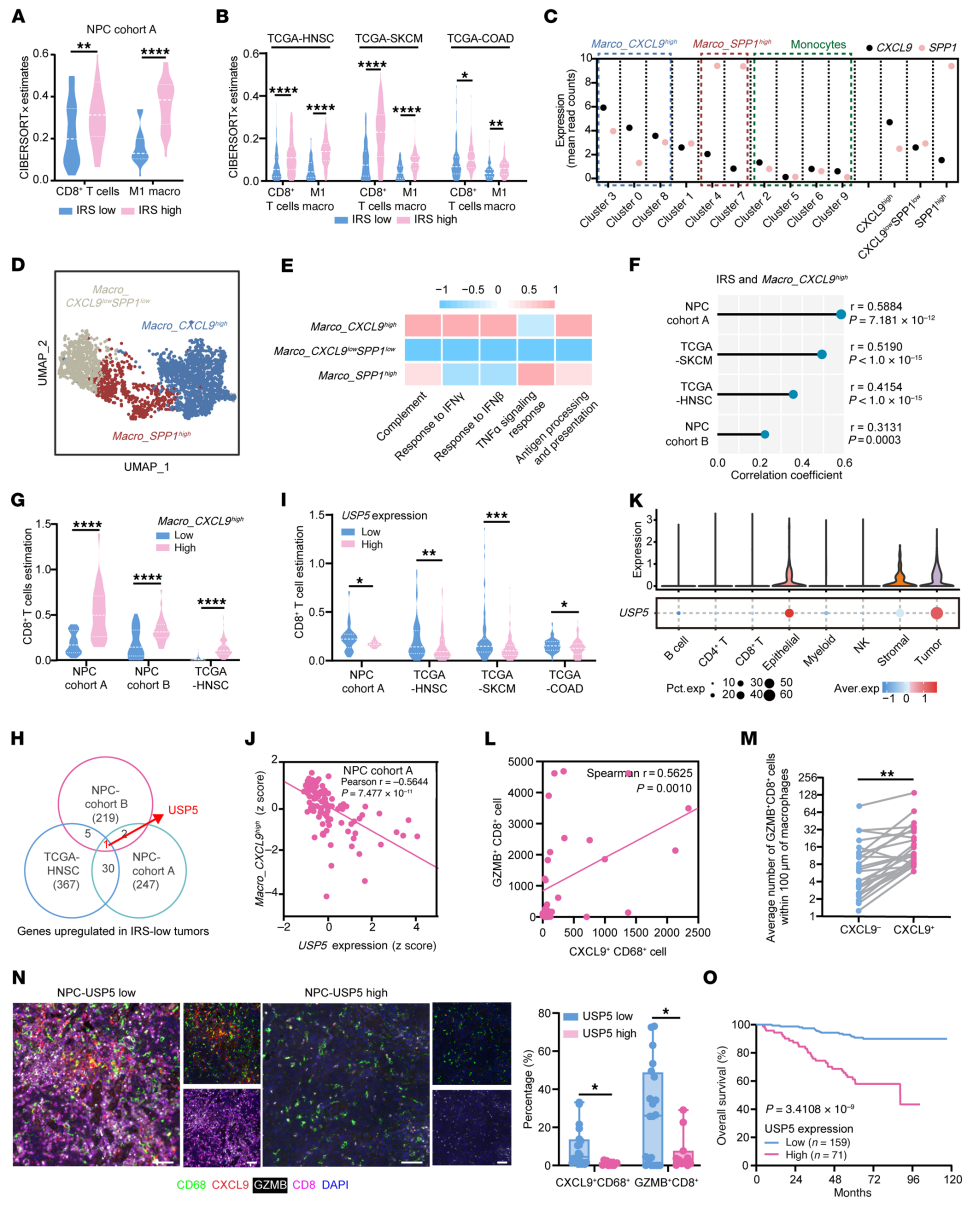
Tumor-intrinsic USP5 inversely correlates with type I IFN–mediated CXCL9^+^ macrophage–CD8^+^ T cell antitumor immunity. (**A**) CIBERSORTx analysis of CD8^+^ T cells and M1 macrophages in NPC with high or low IRS. (**B**) CIBERSORTx analyses of CD8^+^ T cells and M1 macrophages in HNSC, SKCM, and COAD with high or low IRS. (**C**) Expression of *CXCL9* and *SPP1* in monocyte and macrophage states. (**D**) UMAP visualization of *CXCL9^hi^*, *SPP1^hi^*, and *CXCL9^lo^SPP1^lo^* macrophage clusters from single-cell transcriptomes (2,601 cells) of 15 NPC samples. (**E**) Heatmap of expression of curated gene signatures across *CXCL9^hi^*, *SPP1^hi^*, and *CXCL9^lo^SPP1^lo^* macrophages. (**F**) The correlation between IRS and *CXCL9^hi^* macrophages in NPC, SKCM, and HNSC. (**G**) CIBERSORTx analyses of CD8^+^ T cells in NPC and HNSC with high or low *CXCL9^hi^* macrophages. (**H**) A Venn diagram showing *USP5* is the only gene upregulated in both IRS-low NPC and HNSC. (**I**) Immune estimation of CD8^+^ T cells in NPC, HNSC, SKCM, and COAD with high or low *USP5* expression. (**J**) Correlation between *USP5* expression and *CXCL9^hi^* macrophages in NPC. (**K**) *USP5* levels in different cell types from single-cell transcriptomes of 15 NPC samples. (**L**) Spearman’s correlation of CXCL9^+^CD68^+^ macrophages and GZMB^+^CD8^+^ T cells in 31 NPC tissues. (**M**) The average number of GZMB^+^CD8^+^ T cells within 100 μm of CXCL9^+^ or CXCL9^–^ macrophages in NPC tissues. (**N**) Representative images and quantitative results for CXCL9^+^CD68^+^ macrophages and GZMB^+^CD8^+^ T cells in NPC with high or low USP5 expression. Scale bars: 50 μm. (**O**) Kaplan-Meier analysis of overall survival based on USP5 expression level (log-rank test). Comparisons were performed using a paired, 2-tailed Student’s *t* test (**M**), 2-way ANOVA with Bonferroni’s multiple-comparison test (**A** and **G**), or multiple *t* tests with Bonferroni’s correction (**B**, **I**, and **N**). *P < 0.05, **P < 0.01, ***P < 0.001, ****P < 0.0001.

To explore the effect of IFN-I signaling on the TME, we first characterized patients from 2 distinct nasopharyngeal carcinoma (NPC) cohorts (19, 20) as well as patients from The Cancer Genome Atlas (TCGA) cohorts by calculating the IFN-I response gene score (IRS). Survival analysis confirmed that patients with low IRS had poorer survival in NPC, skin cutaneous melanoma (SKCM), and colon adenocarcinoma (COAD) (Supplemental Figure 1, A–C; supplemental material available online with this article; https://doi.org/10.1172/JCI195279DS1). Immune infiltration estimation showed that tumors with high IRS exhibited a higher proportion of M1 macrophages alongside enriched CD8^+^ T cells in NPC, head and neck squamous cell carcinoma (HNSC), SKCM, and COAD (Figure 1, A and B, and Supplemental Figure 1D). The IRS was positively correlated with the abundance of M1 macrophages and CD8^+^ T cells in almost all cancer types in TCGA (Supplemental Figure 1E), confirming the relationship between IFN-I response and macrophage–CD8^+^ T cell immunity.

Since the M1/M2 nomenclature of macrophages only roughly represents the activity of macrophages with distinct stimuli (21), we analyzed previously published NPC single-cell RNA-Seq (scRNA-Seq) data (22) and found 10 monocyte/macrophage subpopulations (Supplemental Figure 1F). Among the 6 macrophage subsets, 3 subsets showed high expression levels of the CD8^+^ T cell chemokine *CXCL9*, while 2 subsets exhibited high levels of the secreted phosphoprotein 1 (*SPP1*) (Figure 1C and Supplemental Figure 1G). Thus, we divided them into 3 subgroups as previously described (23): *CXCL9^hi^*, *SPP1^hi^*, and *CXCL9^lo^SPP1^lo^* macrophages (Figure 1D). Gene expression profiles revealed that *CXCL9^hi^* macrophages expressed high levels of IFN-stimulated genes, including *CXCL10*, *GBP1*, *IFIH1*, *IFI44L*, *STAT1*, and *STAT2* (Supplemental Figure 1H and Supplemental Table 1), and the *CXCL9^hi^* subset had better IFN-I and IFN-II responsiveness as well as stronger antigen-presenting capacity (Figure 1E). Furthermore, IRS was positively correlated with *CXCL9^hi^* macrophage abundance in NPC, as well as in HNSC and SKCM (Figure 1F). Also, the tumors with higher *CXCL9^hi^* macrophage infiltration were enriched with CD8^+^ T cells (Figure 1G and Supplemental Figure 1I). The group with higher intratumoral *CXCL9^hi^* macrophage infiltration demonstrated better disease-free survival in patients with NPC and longer overall survival in patients with HNSC, SKCM, or COAD (Supplemental Figure 1, J–M). Our data suggest that intratumoral IFN-I–responsive CXCL9*^hi^* macrophages mediate CD8^+^ T cell antitumor immunity.

### Tumor-intrinsic USP5 impairs CXCL9^hi^ macrophage–mediated antitumor immunity.

We tried to identify oncogenic targets that could impair IFN-I–mediated macrophage and CD8^+^ T cell antitumor immunity. By comparing gene expression profiles between NPC and HNSC with high or low IRS, we found that deubiquitinase *USP5* was the only gene highly upregulated in IRS-low tumors (Figure 1H), which was also demonstrated in SKCM and COAD (Supplemental Figure 2A). Immune infiltration analysis showed that tumors with higher *USP5* expression contained fewer CD8^+^ T cells and macrophages (Figure 1I and Supplemental Figure 2, B and C), as well as fewer *CXCL9^hi^* macrophages in NPC, HNSC, and SKCM (Figure 1J and Supplemental Figure 2D). Based on the scRNA-Seq data of NPC, melanoma, and colon cancer, we found that *USP5* was dominantly expressed in tumor cells (Figure 1K and Supplemental Figure 2, E and F), indicating that tumor-intrinsic *USP5* might inhibit IFN-I response and *CXCL9^hi^* macrophage–CD8^+^ T cell antitumor immunity.

To test the impact of *USP5* on antitumor immunity, we performed IHC staining of USP5 protein and multiplex immunofluorescent staining of CD68^+^, CXCL9^+^, CD8^+^, and GZMB^+^ cells in 31 NPC tissues. In line with the above finding, USP5 staining was mainly located in tumor cells (Supplemental Figure 2G). The tumor-infiltrating GZMB^+^CD8^+^ T cells were spatially adjacent to CXCL9^+^CD68^+^ macrophages, which were positively correlated (Figure 1, L and M). Moreover, USP5 expression was inversely correlated with the abundance of infiltrating CXCL9^+^CD68^+^ macrophages and GZMB^+^CD8^+^ T cells (Figure 1N). These data suggest that the tumor-intrinsic USP5 could impair IFN-I–activated CXCL9^+^ macrophage and CD8^+^ T cell antitumor immunity.

We further performed IHC staining in a cohort of 230 NPC samples and dichotomized the patients into a high or low USP5 expression group (Supplemental Table 2) for Kaplan-Meier analysis, which revealed that the patients with high USP5 expression had shorter overall, disease-free, and distant metastasis–free survival rates (Figure 1O and Supplemental Figure 2H). Multivariate Cox regression analysis showed that USP5 expression was an independent factor for poor prognosis in NPC (Supplemental Figure 2I). Similarly, higher *USP5* was a risk factor for overall survival in cancers such as HNSC and SKCM (Supplemental Figure 2J). Overall, our results suggest that high USP5 expression indicates poor antitumor immune response and prognosis in patients with tumors.

### USP5 depletion boosts radiation-induced macrophage-mediated antitumor immunity through tumor cell–intrinsic IFN-I signaling.

**Figure 2:**
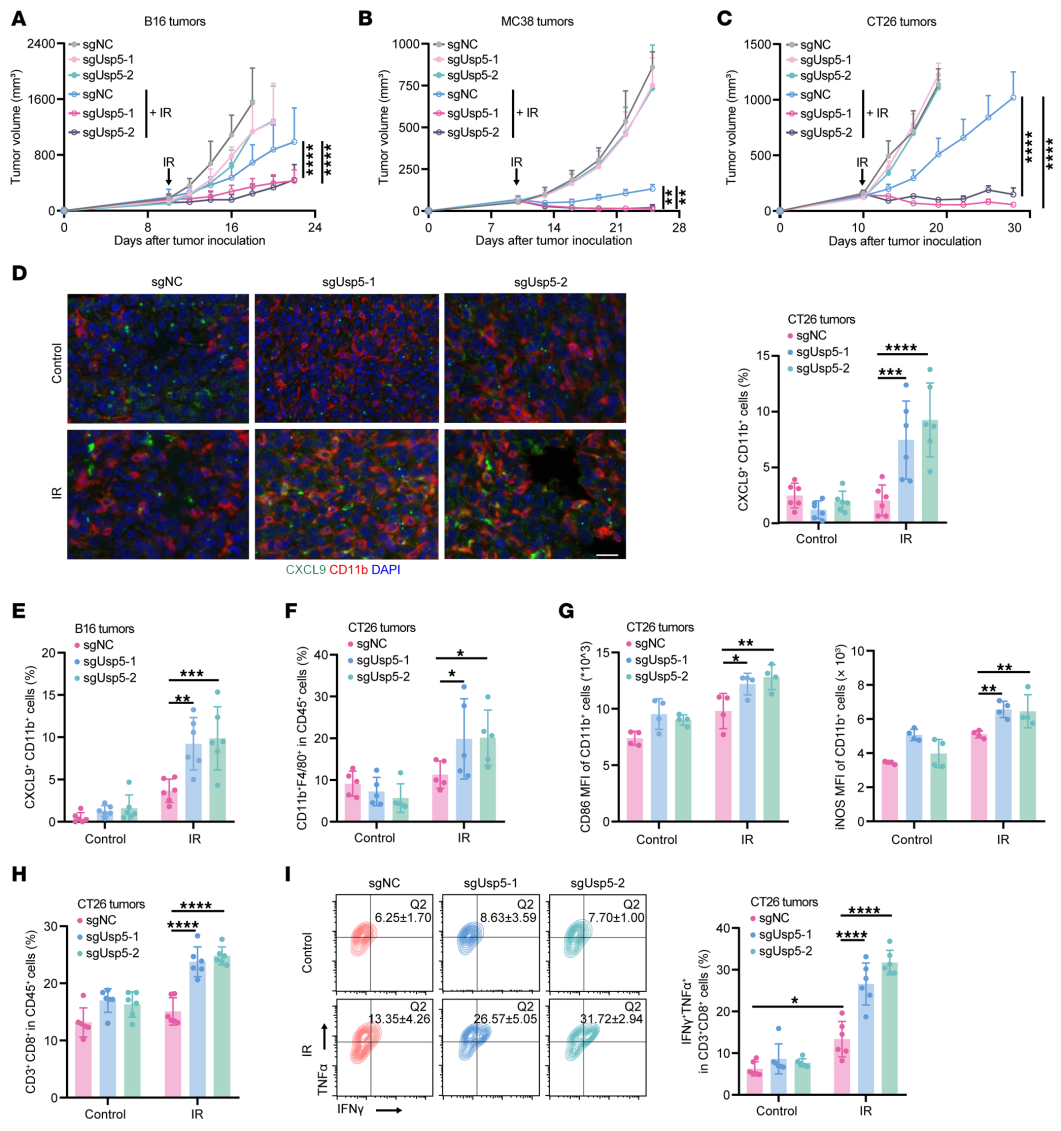
USP5 depletion boosts radiation-induced CXCL9^+^ macrophages and CD8^+^ T cell antitumor immunity. (**A**–**C**) Control and Usp5-KO B16, MC28, and CT26 murine tumor cells were inoculated into C57BL/6 or BALB/c mice. Mice in the irradiation-treated groups were subjected to focal radiation with a single fraction of 15 Gy on day 10 after tumor cell inoculation. The growth rates of B16 tumors (**A**), MC38 tumors (**B**), and CT26 tumors (**C**) were monitored and reported. (**D**) Representative images and quantitative results of multiplex immunofluorescent staining for CXCL9^+^CD11b^+^ macrophages in control and Usp5-depleted CT26 tumors (*n* = 6 per group). Scale bar: 20 μm. (**E**) Quantitative results of CXCL9^+^CD11b^+^ macrophages in control and Usp5-depleted B16 tumors (*n* = 6 per group). (**F**) Flow cytometric results showing the increased tumor-infiltrating CD11b^+^F4/80^+^ macrophages in USP5-depleted CT26 tumors after irradiation (*n* = 5 in each group). (**G**) The CD86 and iNOS expression levels in CD11b^+^ cells in CT26 tumors (*n* = 4 per group). (**H** and **I**) The proportion of CD3^+^CD8^+^ T cells (**G**, *n* = 6 per group) and IFN-γ^+^TNF-α^+^ subsets (**I**, *n* = 5 per group) in CT26 tumors. Comparisons were performed using 2-way ANOVA with Bonferroni’s test for multiple comparisons (**A**–**I**). *P < 0.05, **P < 0.01, ***P < 0.001, ****P < 0.0001.

To explore whether and how USP5 interrupts antitumor immunity, we employed the B16 murine melanoma and CT26 and MC38 murine colon cancer models. The Usp5-WT or -KO murine tumor cells were exposed to radiation or not, and USP5 depletion did not affect cell growth and viability in vitro (Supplemental Figure 3, A–D). The irradiation-induced *Ifnb1* expression was also comparable between WT and Usp5-depleted cells (Supplemental Figure 3E) (24, 25). However, we found that the Usp5-depleted tumors displayed obvious growth retardation after radiation treatment, and the survival of mice bearing Usp5-deficient tumors was significantly prolonged (Figure 2, A–C, and Supplemental Figure 3, F–H). Multiplex immunofluorescence showed enriched CXCL9^+^CD11b^+^ macrophages in Usp5-deficient CT26 and B16 tumors (Figure 2, D and E). Moreover, KO of Usp5 increased costimulatory protein CD86 and inducible nitric oxide synthase (iNOS) in macrophages, indicating their enhanced immune-activating property (Figure 2, F and G). Likewise, the number of infiltrating CD3^+^CD8^+^ T cells and their cytotoxic IFN-γ^+^TNF-α^+^ subpopulation were increased in CT26 tumors (Figure 2, H and I). The enriched macrophages and CD3^+^CD8^+^ T cells were also observed in Usp5-depleted B16 melanoma (Supplemental Figure 3I). These data confirm that tumor-intrinsic USP5 can inhibit radiation-mediated *CXCL9^+^* macrophage and CD8^+^ T cell antitumor immunity.

**Figure 3:**
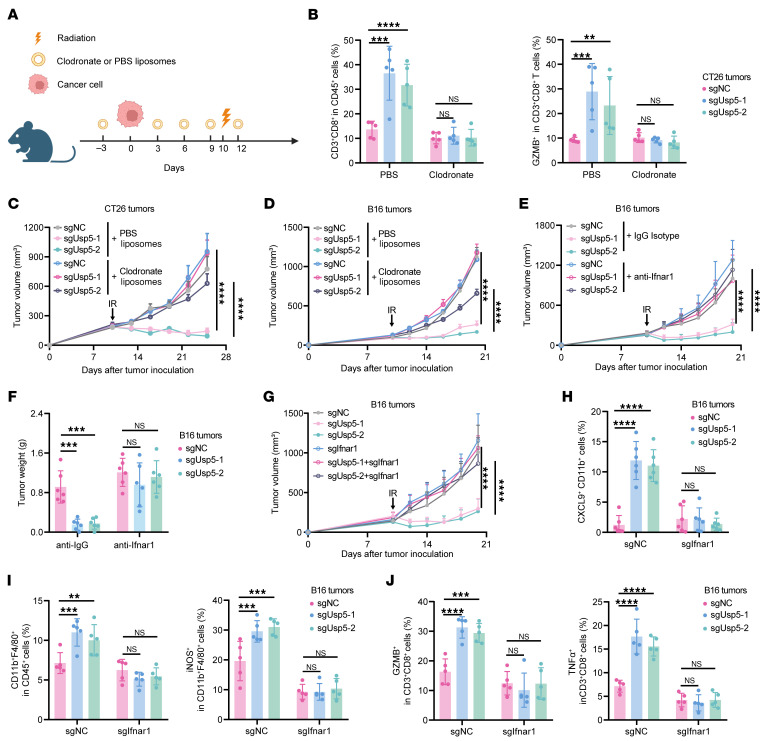
USP5 depletion promotes CXCL9^+^ macrophages and CD8^+^ T cell antitumor immunity in a tumor-intrinsic IFN-I signaling–dependent manner. (**A**–**D**) Every 3 days, 100 μL PBS or clodronate liposome was intratumorally injected, starting 3 days before CT26 or B16 tumor inoculation. The CD3^+^CD8^+^ T cells and their GZMB^+^ T cell subset in CT26 tumors were measured (**B**, *n* = 5 per group). The growth rates of CT26 tumors (**C**) and B16 tumors (**D**) are reported (*n* = 6 per group). (**E** and **F**) Anti-Ifnar1 antibody was intraperitoneally administered (days 7, 10, and 13) to C57BL/6 mice bearing control or Usp5-deficient B16 tumors. The tumor growth (**E**) and tumor weights (**F**) are reported (*n* = 6 per group). (**G**–**J**). Ifnar1-intact or -deficient B16 tumor cells were inoculated into C57BL/6 mice, and the tumors were subjected to radiation. The tumor growth (**G**, *n* = 6 per group), CXCL9^+^CD11b^+^ macrophages (**H**, *n* = 6 per group), proportions of total macrophages and iNOS^+^ subsets (**I**, *n* = 5 per group), and GZMB^+^CD8^+^ or TNF-α^+^CD8^+^ T cells (**J**, *n* = 5 per group) are reported. The data are presented as mean ± SD. Comparisons were performed using 2-way ANOVA with Bonferroni’s test for multiple comparisons (**B**–**J**). **P < 0.01, ***P < 0.001, ****P < 0.0001.

To clarify whether the enrichment of activated macrophages is a critical event in USP5 KO–induced enhancement of CD8^+^ T cell antitumor immunity and radiotherapy efficacy, we depleted infiltrating macrophages by intratumoral administration of clodronate liposomes (26, 27) (Figure 3A) and found that the infiltration of CD3^+^CD8^+^ T cells and their cytotoxicity were significantly decreased along with macrophages (Figure 3B and Supplemental Figure 4A). As a result of impaired antitumor immunity, the enhanced radiotherapy efficacy of Usp5-deficient CT26 and B16 tumors was completely reversed upon macrophage depletion (Figure 3, C and D, and Supplemental Figure 4, B–E). In addition, both systematic Ifnar1 blocking and KO of tumor cell–intrinsic Ifnar1 reversed the growth retardation of Usp5-depleted murine B16 melanomas (Figure 3, E–G, and Supplemental Figure 4, F–H). CXCL9^+^ macrophages were reduced in Ifnar1-depleted tumors, indicated by multiplex immunofluorescence (Figure 3H). Also, the increased infiltrating macrophages, iNOS^+^ macrophage subpopulation, and GZMB^+^CD3^+^CD8^+^ or TNF-α^+^CD3^+^CD8^+^ T cells in Usp5-deficient tumors were also significantly decreased upon Ifnar1 depletion (Figure 3, I and J, and Supplemental Figure 4I). These findings suggest that USP5 inhibits tumor cell–intrinsic IFN-I signaling–dependent CXCL9^+^ macrophage and CD8^+^ T cell antitumor immunity.

### USP5 depletion facilitates the transfer of tumor-intrinsic p-STAT1/2 to macrophages.

**Figure 4:**
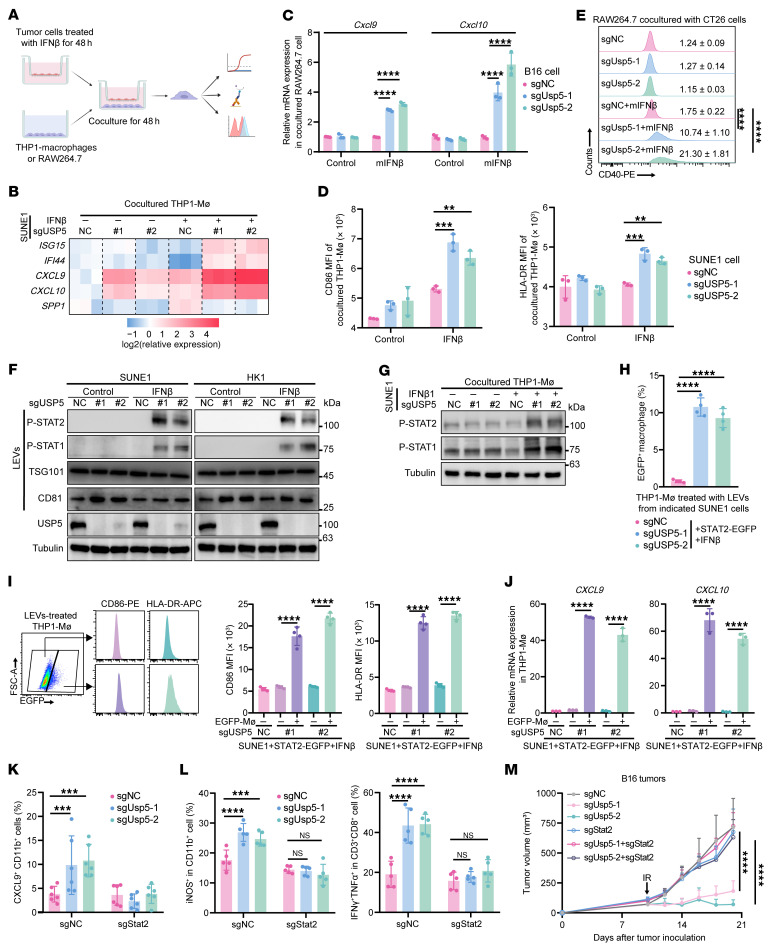
USP5 depletion in tumor cells induces and activates CXCL9^+^ macrophages. (**A**) The coculture system. Human or mouse tumor cells were pretreated with IFN-β for 48 hours and cocultured with THP1-derived macrophages (THP1-MΦ) or RAW264.7 cells for another 48 hours. The macrophages were collected for analysis. (**B**) Heatmap showing the *ISG15*, *IFI44*, *CXCL9*, *CXCL10*, and *SPP1* mRNA expression in cocultured THP1-MΦ. (**C**) Relative *Cxcl9* and *Cxcl10* expression in cocultured RAW264.7 cells. (**D**) CD86 and HLA-DR expression on cocultured THP1-MΦ. (**E**) CD40 expression on RAW264.7 cells cocultured with Usp5-deficient CT26 cells. (**F**) p-STAT2 and p-STAT1 in large extracellular vesicles (LEVs) from the culture medium of USP5-deficient SUNE1 and HK1 cells after IFN-β treatment. (**G**) p-STAT1 and p-STAT2 levels in THP1-MΦ after coculture with SUNE1 cells. (**H**–**J**) THP1-MΦ were treated with LEVs collected from the culture medium of STAT2-EGFP–transfected control and USP5-deficient SUNE1 cells after IFN-β treatment. The proportion of EGFP^+^ macrophages (**H**), CD86 and HLA-DR expression (**I**), and relative *CXCL9* and *CXCL1* expression (**J**) in EGFP^+^ and EGFP^–^ macrophages were measured. (**K**–**M**) Indicated B16 tumors were subjected to irradiation on day 10 after inoculation. The proportions of CXCL9^+^CD11b^+^ macrophages measured by multiplex immunofluorescent staining (**K**, *n* = 6 per group), the iNOS^+^CD11b^+^ macrophages and IFN-γ^+^TNF-α^+^CD8^+^ T cells measured by flow cytometry (**L**, *n* = 5 per group), and tumor growth (**M**, *n* = 6 per group) are reported. The results are representative of 3 independent experiments (**B**–**J**). Data are presented as mean ± SD. Comparisons were performed using 1-way ANOVA (**H**) or 2-way ANOVA with Bonferroni’s test (**C**–**E** and **I**–**M**) for multiple comparisons. **P < 0.01, ***P < 0.001, ****P < 0.0001.

To clarify the impact of USP5 on tumor-intrinsic IFN-I signaling–mediated macrophage activation, USP5-depleted tumor cells were pretreated with IFN-β and then cocultured with macrophages (Figure 4A). We found that THP1-derived macrophages (THP1-MΦ) cocultured with IFN-β–treated USP5-deficient tumor cells exhibited upregulated *ISG15*, *IFI44*, *CXCL9*, and *CXCL10* mRNA expression (Figure 4B). Similarly, *Cxcl9* and *Cxcl10* mRNA expression in murine RAW264.7 macrophages was also increased after coculture with IFN-β–treated Usp5-KO B16 tumor cells (Figure 4C). In addition, USP5 depletion in tumor cells upregulated the expression of costimulator CD86 and antigen-presentation molecule HLA-DR on cocultured THP1-MΦ (Figure 4D), and the CD40 protein of murine RAW264.7 macrophages was also upregulated after coculture with IFN-β–treated Usp5-KO CT26 cells (Figure 4E), confirming that depletion of tumor-intrinsic USP5 could induce IFN-I–responsive *CXCL9^hi^* macrophages and augment their immune-activating capacity.

How tumor-intrinsic IFN-I signaling in USP5-depleted cells activates macrophages warrants further investigation. We inquired whether USP5 inhibited macrophage activation via STAT1 or STAT2, since they are key transducers of IFN-I signaling. However, USP5 deficiency did not affect IFN-I response in tumor cells because neither p-STAT1 and p-STAT2 levels nor IFN-stimulated gene expression were changed in USP5-depleted cells (Supplemental Figure 5, A and B). We performed gene set enrichment analysis (GSEA) and identified enrichment of extracellular transport and exocytosis in NPC tumor cells with low USP5 expression (Supplemental Figure 5C). Recent studies have reported that tumor cell–intrinsic proteins, such as activated STING and PTEN, can be secreted, thus facilitating their function in recipient macrophages (28, 29). We therefore inquired whether p-STATs in tumors could be secreted via EVs and enhance IFN-I response in macrophages after being captured. Through the isolation of large EVs (LEVs) and classical exosomes from culture medium, we found p-STAT1 and p-STAT2 in LEVs, but not in classical exosomes, from USP5-depleted cancer cells after IFN-β treatment (Figure 4F and Supplemental Figure 5, D and E). Interestingly, Western blotting showed increased p-STAT1 and p-STAT2 levels in THP1-MΦ cocultured with IFN-β–treated USP5-deficient cancer cells (Figure 4G). Since STAT2 interacted with USP5 after IFN-β treatment and the phosphorylation-deficient STAT2 lost its binding potential (Supplemental Figure 5, F and G), we then knocked out STAT2 in USP5-deficient cancer cells and found that it eliminated the secretion of p-STAT2 and p-STAT1 (Supplemental Figure 5, H and I). STAT2 deficiency in NPC cells abolished the increase in p-STAT1 and p-STAT2 in cocultured THP1-MΦ (Supplemental Figure 5J), implying the transfer of p-STATs from tumor cells to macrophages.

To confirm that p-STATs secreted by tumor cells can enter macrophages, THP1-MΦ were cocultured with STAT2-FLAG–transfected and IFN-β–treated cancer cells. IP of IRF9 in THP1-MΦ coprecipitated the FLAG-tagged STAT2 derived from tumor cells (Supplemental Figure 5K). We further treated THP1-MΦ with LEVs isolated from STAT2-EGFP–transfected and IFN-β–treated cancer cells. As expected, flow cytometric analysis showed the occurrence of an EGFP^+^ subpopulation in THP1-MΦ treated with LEVs from the USP5-depleted tumor cells (Figure 4H). EGFP^+^ THP1-MΦ exhibited higher expression of surface CD86 and HLA-DR (Figure 4I) as well as *CXCL9* and *CXCL10* mRNA, but lower *SPP1* compared with their EGFP^–^ counterparts (Figure 4J and Supplemental Figure 5L). STAT2 depletion in USP5-deficient cancer cells significantly reduced upregulated *CXCL9* and *CXCL10* mRNA, as well as CD86 and HLA-DR protein of cocultured THP1-MΦ (Supplemental Figure 6, A and B); these reductions were rescued by restoring WT-STAT2 but not the Y690A mutant in USP5-depleted cells (Supplemental Figure 6, C–E). In line with these findings, depletion of tumor-intrinsic Stat2 significantly reduced intratumoral CXCL9^+^ macrophages (Figure 4K), iNOS^+^ macrophages, and IFN-γ^+^TNF-α^+^CD8^+^ T cell infiltration in Usp5-deficient B16 melanomas in vivo (Figure 4L and Supplemental Figure 6, F and G). Consequently, the radiation efficacy of Usp5-deficient tumors was greatly impaired upon Stat2 depletion (Figure 4M and Supplemental Figure 6, H and I). Taken together, our results indicate that depletion of tumor-intrinsic USP5 triggers the transfer of p-STATs from tumor cells to macrophages, inducing CXCL9^+^ macrophage and CD8^+^ T cell antitumor immunity.

### USP5 deficiency promotes LC3-dependent secretory autophagy of p-STAT1/2.

**Figure 5:**
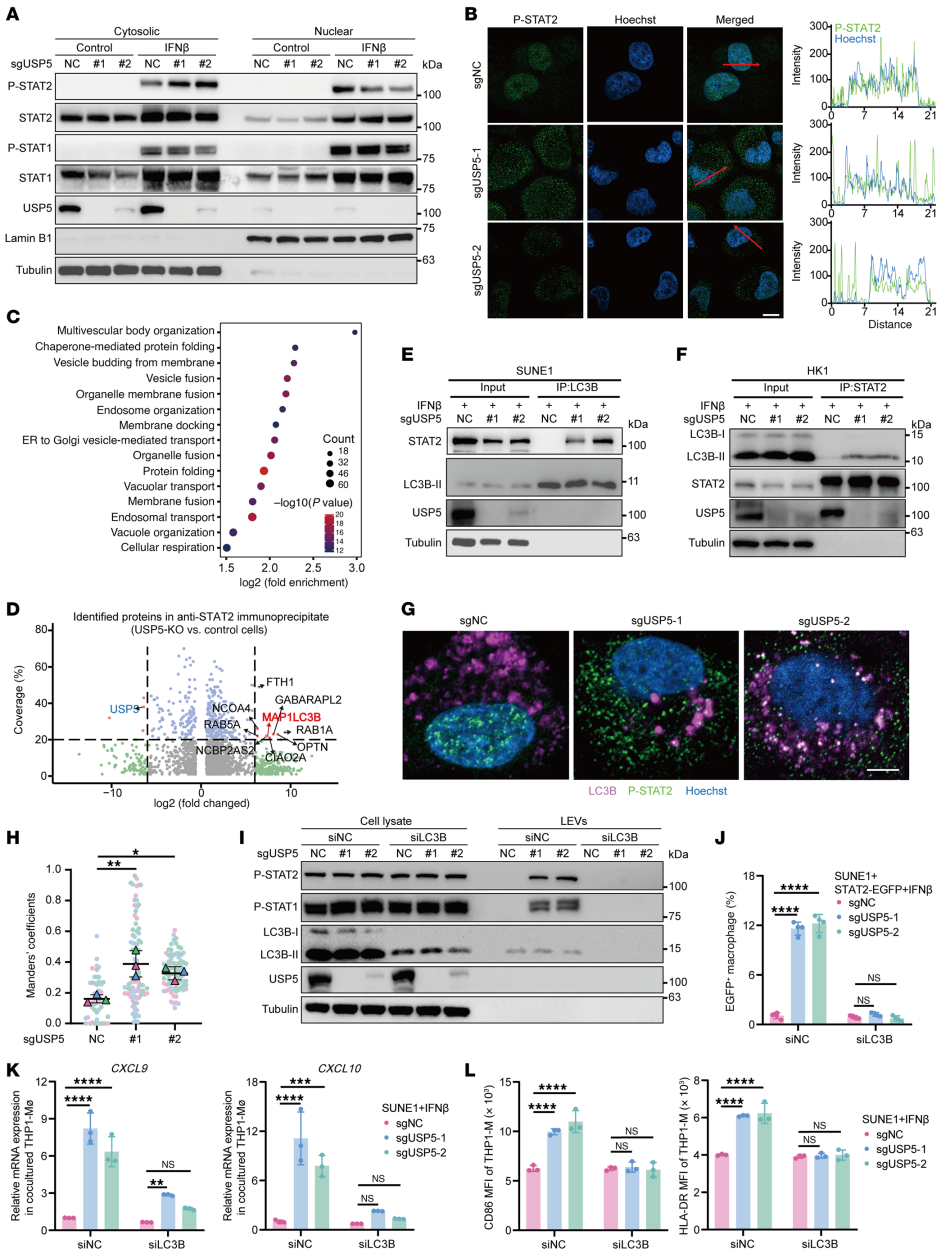
USP5 deficiency promotes LC3-dependent secretory autophagy of p-STAT1/2. (**A**) The cytosolic and nuclear p-STAT1 and p-STAT2 levels in WT and USP5-deficient SUNE1 cells. (**B**) Representative confocal microscopy images and line scan analysis showing the different localization of p-STAT2 in WT or USP5-deficient SUNE1 cells. Scale bar: 10 μm. (**C**) Bubble diagram shows the enriched biological process of anti-STAT2 immunoprecipitated proteins in USP5-deficient SUNE1 cells after IFN-β treatment. (**D**) Volcano plot shows 1,353 upregulated and 566 downregulated proteins (FC ≥ 1.5 and *P* < 0.05) in anti-STAT2 immunoprecipitates from USP5-deficient compared with control SUNE1 cells. (**E** and **F**) Co-IP shows the interaction between STAT2 and LC3B-II in USP5-deficient SUNE1 cells (**E**) and HK1 cells (**F**) after IFN-β treatment. (**G** and **H**) Representative immunofluorescence assay images (**G**) showing the colocalization of p-STAT2 and LC3B in USP5-deficient SUNE1 cells after IFN-β treatment. Scale bar: 10 μm. (**H**) The Manders’ colocalization coefficients are reported. (**I**) The decreased p-STAT2 and p-STAT1 in large extracellular vesicles (LEVs) from the culture medium of USP5-deficient SUNE1 after LC3B knockdown. (**J**) LC3B knockdown in STAT2-EGFP SUNE1 cells decreased the EGFP^+^ population in coculture with THP1-MΦ. (**K** and **L**) Control and USP5-deficient SUNE1 cells were transfected with si-LC3B and treated with IFN-β for 48 hours. LEVs were collected from the culture medium and used to treat the THP1-MΦ. The relative *CXCL*9 and *CXCL10* expression in macrophages was measured by RT-qPCR (**K**); the CD86 and HLA-DR expression on macrophages was measured by flow cytometry (**L**). The results are representative of 3 independent experiments (**A**, **B**, and **E**–**L**). The data are presented as mean ± SD (**H** and **J**–**L**). Comparisons were performed using 1-way ANOVA (**H**) or 2-way ANOVA (**J**–**L**) with Bonferroni’s test for multiple comparisons. *P < 0.05, **P < 0.01, ***P < 0.001, ****P < 0.0001.

The mechanism by which the tumoral p-STATs are loaded into LEVs and secreted from USP5-deficient cells remains unexplored. Our findings indicated that USP5 depletion caused p-STAT2 accumulation in the cytoplasm of NPC cells (Figure 5A), and the accumulated p-STAT2 formed puncta, as visualized by immunofluorescence and intensity analysis (Figure 5B and Supplemental Figure 7A). To search for key proteins that may facilitate the loading of STAT2 into LEVs, we performed mass spectrometry of anti-STAT2 immunoprecipitates from IFN-β–treated USP5-intact and USP5-deficient cells. GSEA of the upregulated immunoprecipitated proteins from USP5-depleted cells showed enhanced protein transportation and vesicular trafficking (Figure 5C and Supplemental Table 3). Notably, proteins relevant to autophagy, including MAP1LC3B, OPTN, RAB5A, and RAB1A, were found to be upregulated in anti-STAT2 immunoprecipitates from USP5-depleted SUNE1 cells (Figure 5D and Supplemental Figure 7B).

The LC3-conjugation machinery can promote the loading of LC3-interacting proteins into EVs (30), presenting a new mechanism for secretory autophagy. Since the STAT2 protein contains an LC3-interaction region (Supplemental Figure 7C), we hypothesized that the interaction between STAT2 and LC3B facilitated p-STAT1/2 secretion. As expected, co-IP verified the interaction between STAT2 and LC3B-II in USP5-depleted NPC cells (Figure 5, E and F), and a mutation in the LC3-interaction region domain of STAT2 eliminated their binding (Supplemental Figure 7D). The STAT2-LC3B interaction was validated by the enhanced colocalization of p-STAT2 and LC3B in the cytoplasm (Figure 5, G and H), indicating that p-STAT2 could bind to LC3B-II in USP5-deficient NPC cells after IFN-β treatment. Furthermore, knockdown of LC3B in cancer cells substantially decreased the secreted–p-STAT1/2 and EGFP^+^ THP1-MΦ subpopulation (Figure 5, I and J). The upregulated *CXCL9* and *CXCL10* mRNA, as well as CD86 and HLA-DR protein of cocultured THP1-MΦ, were abolished upon tumoral LC3B reduction (Figure 5, K and L).

Since LC3-dependent EV loading and secretion are usually increased upon the inhibition of autophagy-related protein degradation (31), and USP5 has been reported to promote autophagy by protecting BECN1 from degradation (32), we investigated whether p-STAT1/2 secretion is autophagy dependent. Our results showed that lysosomal function was not impaired in USP5-depleted NPC cells (Supplemental Figure 8A). However, the inhibition of lysosomal acidification by chloroquine, rather than the inhibition of autophagy initiation, was associated with a decreased p-STAT2 level in USP5-depleted NPC cells (Supplemental Figure 8B). Both chloroquine and bafilomycin A1 increased the p-STAT1/2 levels in LEVs (Supplemental Figure 8C). In contrast, the knockdown of ATG5, which mediates LC3 lipidation, resulted in decreased p-STAT1/2 levels in LEVs from USP5-depleted NPC cells, which is consistent with the prior finding that LC3-dependent EV loading and secretion is ATG5 dependent (30, 31) (Supplemental Figure 8D). In addition, LEVs from IFN-β–treated and chloroquine-treated USP5-deficient cells further increased *CXCL9* and *CXCL10* expression of THP1-MΦ (Supplemental Figure 8E). Our data suggest that USP5 depletion in tumor cells drives LC3-related secretory autophagy of p-STAT1/2, resulting in intercellular transference of IFN-I signaling and macrophage activation.

### Ubiquitination of STAT2 permits its binding with LC3 for secretory autophagy.

**Figure 6:**
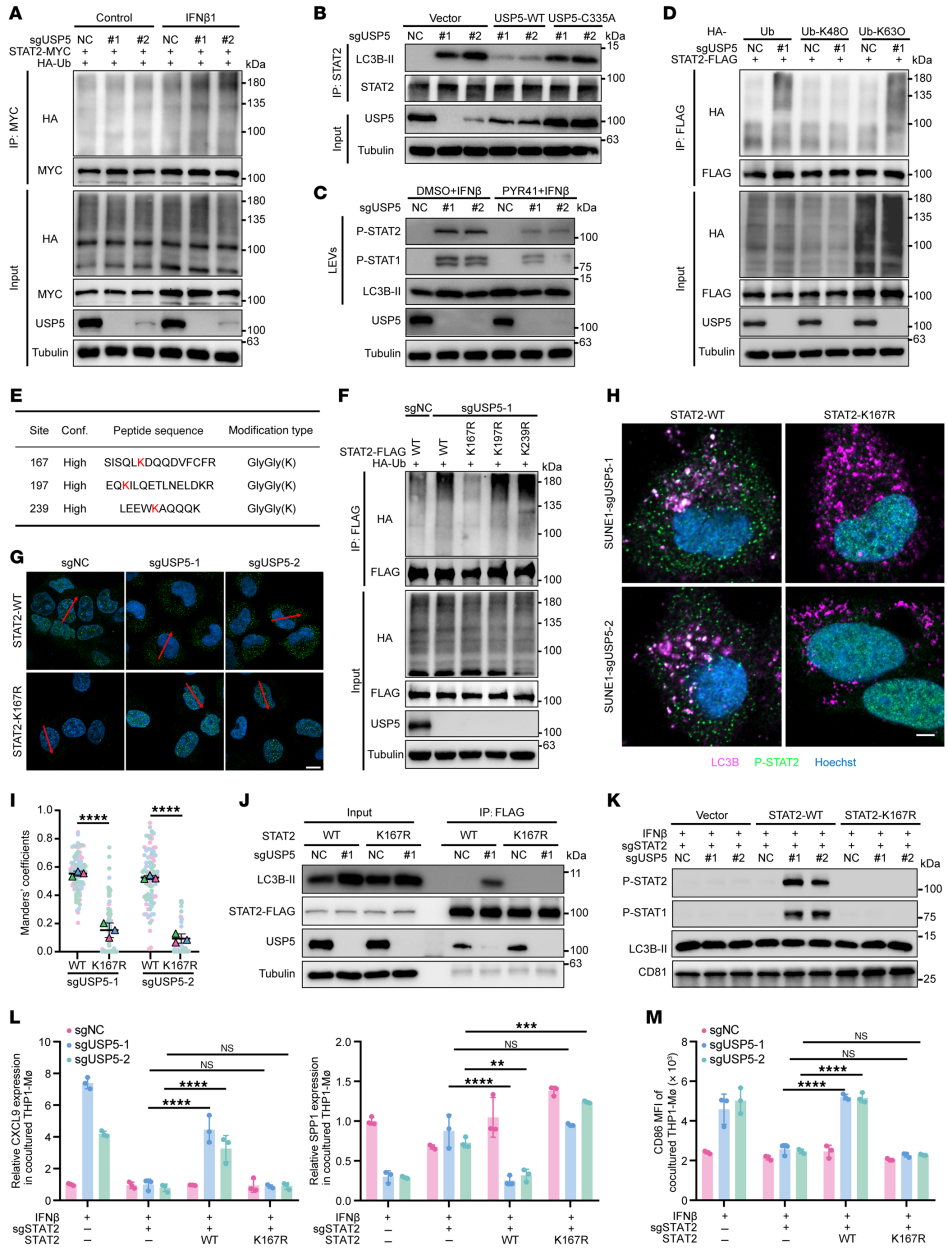
USP5 depletion enhances STAT2 ubiquitination to permit its binding with LC3 for secretory autophagy. (**A**) Ubiquitination of STAT2 in control and USP5-depleted cells after IFN-β treatment. (**B**) The binding of STAT2 and LC3B-II in USP5-deficient SUNE1 cells after WT or C335A-mutated USP5 restoration and IFN-β treatment. (**C**) PRY41 treatment decreased p-STAT1 and p-STAT2 levels in LEVs from USP5-deficient SUNE1 cells. (**D**) Knockout of USP5 increased K63O-linked ubiquitination of STAT2 in IFN-β–treated SUNE1 cells. (**E**) Mass spectrometry analysis of STAT2 ubiquitination sites. (**F**) Ubiquitination of FLAG-tagged WT and K167R, K197R, and K239R-STAT2 mutants in USP5-deficient SUNE1 cells. (**G**) Representative confocal microscopy images showing the different localization of phosphorylated WT or K167R-mutated STAT2 in WT or USP5-deficient SUNE1 cells. Scale bar: 10 μm. (**H** and **I**) The colocalization relationship and Manders’ colocalization coefficients between p-STAT2 and LC3B in STAT2- and USP5-deficient SUNE1 cells with WT STAT2 or K167R-mutant reconstruction and IFN-β treatment. Scale bar: 10 μm. (**J**) The binding between LC3B with WT or K167R-STAT2 in SUNE1 cells after IFN-β treatment. (**K**) The p-STAT1 and p-STAT2 levels in LEVs collected from the culture medium of STAT2-deficient SUNE1 cells transfected with WT or K167R-mutated STAT2 after IFN-β treatment. (**L** and **M**) Control and USP5-deficient SUNE1 cells were transfected with WT STAT2 or K167R-mutant and treated with IFN-β for 48 hours. LEVs were collected from culture medium and treated for THP1-MΦ. The *CXCL*9 and *SPP1* expression in macrophages was measured by RT-qPCR (**L**), and the CD86 expression on macrophages was measured by flow cytometry (**M**). The results are representative of 3 independent experiments (**A**–**D** and **F**–**M**). The data are presented as mean ± SD. Comparisons were performed using 2-way ANOVA with Bonferroni’s test (**I**, **L**, and **M**) for multiple comparisons. **P < 0.01, ***P < 0.001, ****P < 0.0001.

Ubiquitination is critical for cargo selection and loading in secretory autophagy (10). We thus hypothesized that USP5 inhibited the loading of p-STAT2 into LC3-related vesicles by decreasing its ubiquitination. Our results revealed that the ubiquitination of STAT2 in USP5-depleted cells was markedly increased after IFN-β treatment (Figure 6A and Supplemental Figure 9A). The restoration of WT USP5 but not its deubiquitinase-inactivated mutant C335A decreased the ubiquitination of STAT2, weakened its interaction with LC3B-II, and reduced p-STAT2 in LEVs (Figure 6B and Supplemental Figure 9, B and C). In addition, treatment with PYR41, a ubiquitin-activating enzyme E1 inhibitor, and MLN4924, an E3 ligase inhibitor, did not decrease total levels of p-STAT1/2, but markedly reduced the cytosolic p-STAT2 puncta as well as p-STAT1/2 levels in LEVs from USP5-depleted cancer cells (Figure 6C and Supplemental Figure 9, D and E). Moreover, the EGFP^+^ subpopulation in THP1-MΦ was significantly decreased upon treatment with LEVs from the PYR41-pretreated SUNE1 cells (Supplemental Figure 9F), suggesting that ubiquitination promotes the loading of p-STAT2 into LC3 vesicles and its subsequent secretion.

We next inquired about the ubiquitin type that promotes the loading of p-STAT2. To this end, we expressed ubiquitin variants (K6, K11, K27, K29, K33, K48, or K63-only) alongside FLAG-STAT2 in either control or USP5-depleted SUNE1 cells, followed by IFN-β treatment. Our results demonstrated that K63-only ubiquitin substantially enhanced STAT2 ubiquitylation in USP5-deficient cells. In contrast, the K63R ubiquitin mutant failed to increase STAT2 ubiquitination (Figure 6D and Supplemental Figure 10, A–C). To search for ubiquitinated sites in STAT2, we performed mass spectrometry analysis of anti-STAT2 immunoprecipitates from USP5-depleted SUNE1 cells after IFN-β treatment. This analysis identified 3 potential ubiquitinated sites (K167, K197, and K239) in STAT2 (Figure 6E and Supplemental Figure 10D). However, upon performing the lysine (K) to arginine (R) point mutations, only residue K167 was confirmed as the critical residue for STAT2 ubiquitylation (Figure 6F). These results indicate that residue K167 of STAT2 undergoes K63-linked ubiquitination.

To clarify whether the K167 ubiquitination is responsible for p-STAT2-LC3B interaction and secretion, we restored the WT or K167R-mutated STAT2 in STAT2-KO SUNE1 cells, and found that the K167R mutation did not impair the phosphorylation of STAT2 induced by IFN-β treatment (Supplemental Figure 10E). The phosphorylated K167R mutant could not form cytosolic p-STAT2 puncta as the WT p-STAT2 did (Figure 6G and Supplemental Figure 10F). Moreover, the K167R mutation abolished the colocalization and interaction of p-STAT2 and LC3B (Figure 6, H–J). Consequently, neither p-STAT2 nor p-STAT1 was detected in LEVs from cells transfected with the K167R mutant (Figure 6K), indicating that this mutant failed to be loaded into LEVs. In addition, LEVs from STAT2-KO NPC with K167R mutant restoration could not increase *CXCL9* mRNA and CD86 protein in THP1-MΦ. *SPP1* mRNA expression was not decreased in THP1-MΦ treated with LEVs from K167R-overexpressing cells (Figure 6, L and M). Our results show that the K63-linked ubiquitination on K167 facilitates the loading of p-STAT2 into the LC3-related LEVs and promotes its secretion.

### Targeting USP5 enhances radiation-induced antitumor immunity and treatment efficacy.

**Figure 7:**
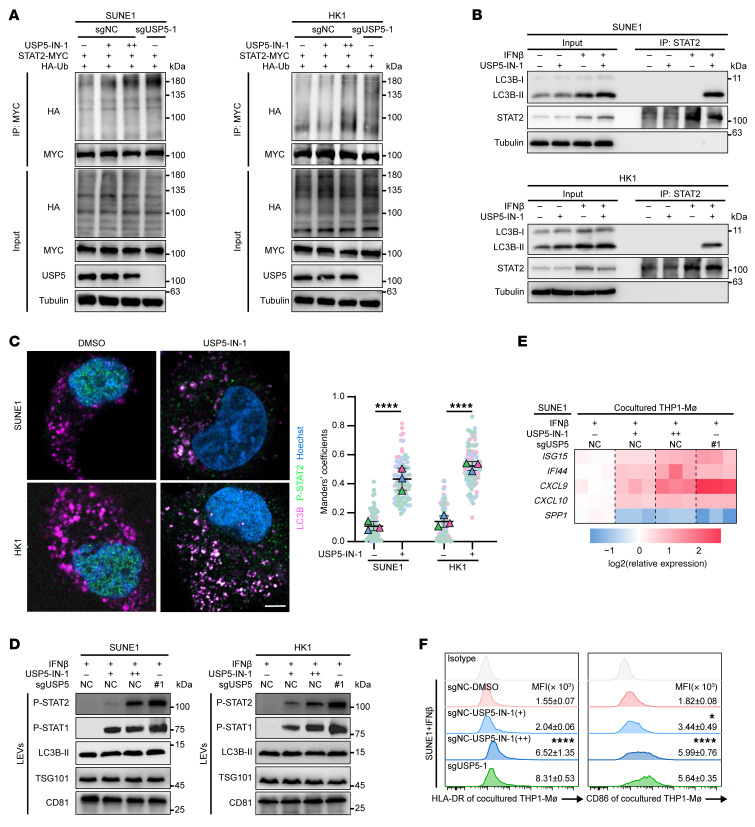
USP5 inhibition increased the ubiquitination and secretion of p-STAT2. (**A**) USP5-IN-1 increased STAT2 ubiquitination in SUNE1 and HK1 cells after IFN-β treatment. (**B**) Co-IP shows that the USP5-IN-1 treatment promotes the interaction between STAT2 and LC3B-II. (**C**) Immunofluorescence assay and representative images show the colocalization of p-STAT2 and LC3B in USP5-IN-1– and IFN-β–treated SUNE1 and HK1 cells. Scale bar: 10 μm. The Manders’ colocalization coefficients are reported. (**D**) Western blot showing the p-STAT1 and p-STAT2 levels in large extracellular vesicles (LEVs) from SUNE1 and HK1 cells treated with USP5-IN-1 and IFN-β. (**E** and **F**) Relative mRNA expression of *ISG15*, *IFI44*, *CXCL9*, *CXCL10*, and *SPP1* (**E**), and HLA-DR and CD86 expression (**F**) in THP1-MΦ after coculture with SUNE1 cells pretreated with DMSO or USP5-IN-1 and IFN-β. The results are representative of 3 independent experiments (**A**–**F**). The data are presented as mean ± SD. Comparisons were performed using 2-way ANOVA with Bonferroni’s test (**C** and **F**) for multiple comparisons. *P < 0.05, ****P < 0.0001.

To investigate the value of USP5 inhibition in enhancing antitumor immunity, the USP5-specific inhibitor USP5-IN-1 (33) was used to treat tumor cells together with IFN-β treatment. We found that USP5-IN-1 increased the ubiquitination of STAT2 in NPC cells treated with IFN-β (Figure 7A). USP5-IN-1 treatment did not affect p-STAT1/2 protein levels but promoted the binding of STAT2 and LC3B-II (Figure 7B and Supplemental Figure 11A). Also, the cytosolic p-STAT2 puncta and the colocalization of p-STAT2 and LC3B were found in NPC cells upon USP5-IN-1 and IFN-β treatment (Figure 7C and Supplemental Figure 11B). USP5-IN-1 increased p-STAT2 and p-STAT1 in LEVs from the IFN-β–treated NPC cells (Figure 7D). Accordingly, USP5 inhibition in NPC cells led to an increase in *CXCL9* and *CXCL10* mRNA and a decrease in *SPP1* mRNA in cocultured THP1-MΦ (Figure 7E). In addition, the expression levels of HLA-DR and CD86 were increased when THP1-MΦ were cocultured with NPC cells pretreated with USP5-IN-1 and IFN-β (Figure 7F). These data suggest that USP5 inhibition may activate macrophages for antitumor immunity.

**Figure 8:**
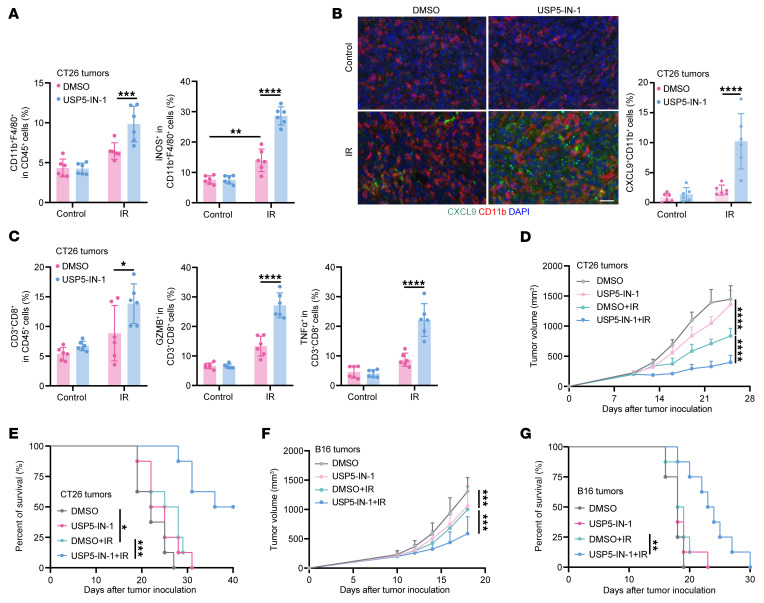
Targeting USP5 enhances CXCL9^+^ macrophage–CD8^+^ T cell antitumor immunity and treatment efficacy of radiotherapy. (**A**) The proportion of intratumoral CD11b^+^F4/80^+^ macrophages and iNOS^+^ macrophages in control and USP5-IN-1–treated CT26 tumors after irradiation (*n* = 6 per group). (**B**) Representative images and quantitative results of multiplex immunofluorescent staining for CXCL9^+^CD11b^+^ macrophages in control and USP5-IN-1–treated CT26 tumors (*n* = 6 per group). Scale bar: 20 μm. (**C**) The proportion of intratumoral CD3^+^CD8^+^ T cells and GZMB^+^ or TNF-α^+^ T cells in control and USP5-IN-1–treated CT26 tumors after irradiation (*n* = 6 per group). (**D** and **E**) CT26 tumor growth and Kaplan-Meier survival curves (log-rank test) of BALB/c mice receiving indicated treatment are reported (*n* = 8 per group). (**F** and **G**) B16 tumor growth and Kaplan-Meier survival curves (log-rank test) of C57BL/6 mice receiving indicated treatment are reported (*n* = 8 per group). The data are presented as mean ± SD. Comparisons were performed using 2-way ANOVA with Bonferroni’s test (**A**–**D** and **F**) for multiple comparisons. *P < 0.05, **P < 0.01, ***P < 0.001, ****P < 0.0001.

We next investigated whether USP5 inhibition could potentiate radiation-induced antitumor immunity and improve radiotherapy efficacy in vivo. BALB/c mice bearing CT16 tumors and C57BL/6 mice bearing B16 tumors were subjected to irradiation and intraperitoneal injection of USP5-IN-1. Flow cytometric analyses demonstrated that USP5-IN-1 treatment increased tumor-infiltrating macrophages and their iNOS^+^ subpopulation in CT26 tumors after irradiation (Figure 8A). Multiplex immunofluorescence further revealed increased CXCL9^+^CD11b^+^ macrophages in CT26 tumors after USP5-IN-1 and irradiation (Figure 8B). The number and the cytotoxicity of intratumoral CD8^+^ T cells were increased upon USP5-IN-1 treatment (Figure 8C). A similar enrichment of CXCL9^+^CD11b^+^ macrophages, iNOS^+^ macrophages, and IFN-γ^+^TNF-α^+^ CD8^+^ T cells was found in B16 tumors with USP5-IN-1 and radiation treatment (Supplemental Figure 11, C–E). As a result of the enhanced antitumor immunity, USP5-IN-1 treatment significantly slowed the growth of CT26 and B16 tumors after irradiation and effectively extended the survival of the tumor-bearing mice (Figure 8, D–G).

We also monitored the toxicity of USP5-IN-1 treatment in mice and found that the body weight of BALB/c and C57BL/6 mice remained stable during USP5-IN-1 treatment, despite weight loss induced by radiotherapy (Supplemental Figure 11, F and G). Routine blood tests showed that USP5-IN-1 treatment did not decrease peripheral red blood cells, white blood cells, or platelets (Supplemental Figure 11, H and I). Additionally, blood biochemical tests showed comparable levels of serous alanine aminotransferase, creatinine, and lactate dehydrogenase between control and USP5-IN-1–treated mice (Supplemental Figure 11, J and K), confirming the safety of USP5-IN-1 treatment. Altogether, our findings demonstrate that USP5 inhibition represents an effective and advisable strategy for enhancing antitumor immune response and improving radiation efficacy.

## Discussion

The roles of innate and adaptive immunity mediated by IFN-I signaling in anticancer treatments, particularly in radiotherapy, have been well demonstrated. However, compared with the extensively studied mechanisms of cGAS/STING and DCs in IFN-I production, the understanding of how IFN-I programs different cell populations within the TME and their underlying mechanisms remains limited. Here, we uncover the intercellular transfer of IFN-I signaling in the TME and demonstrate that tumor cell–intrinsic p-STAT1/2 is transferred to macrophages via secretory autophagy. This process elicits CXCL9^+^ macrophage and CD8^+^ T cell antitumor immunity, thus enhancing the efficacy of radiotherapy.

The macrophage, as an immune cell type, plays a critical role in determining the efficacy of tumor treatment. Consequently, targeting macrophages and reprogramming or activating their function have emerged as prominent therapeutic strategies in cancer therapy (9). The CXCL9^+^ macrophage represents a newly defined macrophage subset characterized by superior immune activation capacity and indicates a favorable prognosis in various cancers (23). Nevertheless, the mechanisms underlying the programming of CXCL9^+^ macrophages remain poorly understood, thereby hindering the development of tumor treatment strategies based on this antitumor population. In the current study, we revealed that tumor-infiltrating CXCL9^+^ macrophages are one of the most important responders to IFN-I. This macrophage population is reprogrammed by the IFN-I signaling transmitted from tumor cells, leading to increased expression of T cell chemokine CXCL9/XCL10 and upregulation of the antigen-presenting machinery, thereby boosting CD8^+^ T cell–mediated antitumor immunity. Our study establishes a connection between intratumoral IFN-I signaling and the CXCL9^+^ macrophage subset, clarifying their critical roles in radiation-induced antitumor immunity and providing a potential macrophage-targeting strategy to enhance radiotherapy efficacy.

The release of damage-associated molecular patterns and the secretion of IFN-β are the two classical mechanisms by which tumor cells activate IFN-I signaling within macrophages (1, 2). Recent studies have shown that the second messenger cyclic guanosine monophosphate–adenosine monophosphate (2′3′-cGAMP) and activated STING in tumor cells can be transmitted to macrophages through cell gap junctions (34–36) or exosomes (28), which also enhances IFN-I signaling in macrophages. Secretory autophagy is a newly discovered cell communication and has not yet been investigated in IFN-regulated antitumor immunity. In this study, we found that p-STAT1 and p-STAT2, the key transducers of IFN-I signaling, can also be transferred from tumor cells to macrophages via secretory autophagy, thereby realizing the transmission and spread of IFN-I signaling between tumor cells and macrophages. Our results reveal a pathway for intercellular IFN signaling transfer between tumors and macrophages, but also raise a question regarding how the p-STAT–containing LEVs enter macrophages. We found that inhibiting phagocytosis of macrophages only slightly reduced the STAT2^–^EGFP^+^ cell population (data not shown), suggesting that phagocytosis may not be the primary way by which macrophages acquire LEVs. Considering the common ways that EVs enter the receptor cells, we hypothesize that LEVs derived from tumors might transfer p-STAT2 to macrophages via plasma membrane fusion, as suggested by previous studies (37). However, further extensive experimental evidence is required to support this hypothesis.

The ubiquitination of STAT2 usually happens after the activation of IFN-I signaling. This process is mainly observed in virus-infected cells, where STAT2 is linked with E3 ligases by the virus-associated proteins, leading to the proteasomal degradation of STAT2 and subsequent suppression of IFN signaling (38, 39), thereby allowing immune evasion by the virus. In this study, we report a mechanism by which the ubiquitination of STAT2 in IFN-treated tumor cells leads to its secretion instead of degradation, ultimately promoting enhanced immune activation. We propose that the secretory autophagy of STAT2 is attributable to its K63-linked ubiquitination, as the loss of this modification eliminates STAT2 secretion. However, it remains unclear whether the K63-linked STAT2 ubiquitination is cell-type specific or is mediated by a specific E3 ligase. Several candidate ligases, such as TRIM25 and TRIM32, were found in the mass spectrometry analysis of anti-STAT2 immunoprecipitates, and their roles in STAT2 ubiquitination have not been reported. Further studies are warranted to identify the specific E3 ligase that mediates the K63-ubiquitination of STAT2 and to explore its potential clinical applications.

Modulating deubiquitinase activity is an emerging therapeutic strategy to inhibit cancer growth or improve responsiveness to anticancer therapies (40). USP5 has been reported as a typical tumor promoter, as it not only stabilizes oncoproteins to promote cancer metastasis but also contributes to treatment resistance in tumors (41–44). Moreover, it promotes T cell exhaustion and tumor immune escape by deubiquitinating and stabilizing PD-L1 in cancer cells and PD-1 in CD8^+^ T cells (45, 46). Our current study further elucidated the immunosuppressive role of tumor-intrinsic USP5 by revealing its inhibition of IFN-I–mediated macrophage reprogramming and antitumor immune responses. Interestingly, USP5 tends to interact with active substrates, such as p-STAT2 in our study and previously reported phosphorylated PD-1 and SNAIL (42, 45), suggesting that it might serve as a checkpoint in intracellular signal transduction. Therefore, USP5 inhibition may effectively suppress tumor malignancy while promoting antitumor immunity. USP5-IN-1 is a newly identified molecular compound that can specifically inhibit the deubiquitinase activity of USP5 (33). Here, we provide evidence that it potentiates the reprogramming of IFN-I–mediated CXCL9^+^ macrophages to elicit a potent CD8^+^ T cell antitumor immune response and encourage radiotherapy efficacy, thereby demonstrating a promising potential for clinical application.

NPC is a malignancy originating from the nasopharyngeal epithelium, which is prevalent in southern China and Southeast Asia (47). Radiotherapy, either alone or in combination with chemo-immunotherapy, is the recommended strategy for patients with nonmetastatic disease (48). However, approximately 20% of patients with advanced-stage NPC experience tumor progression due to resistance to treatment (49–51). Our previous studies have emphasized the importance of the treatment-induced IFN-I response in therapeutic efficacy in NPC (22, 52, 53). Nevertheless, the mechanisms by which immune cells enriched in the TME of NPC (20) are programmed by IFN-I remain to be discovered. In this study, we found that IFN-I is related to CXCL9^+^ macrophages and CD8^+^ T cell immunity in NPC, and then identified that tumor-intrinsic USP5 is a key suppressor of this IFN-I–activated antitumor immunity. In addition, we confirmed the prognostic significance of USP5 expression in patients with NPC. All these findings indicate that USP5 inhibition may be a potential strategy for improving therapeutic efficacy in NPC.

**Figure 9:**
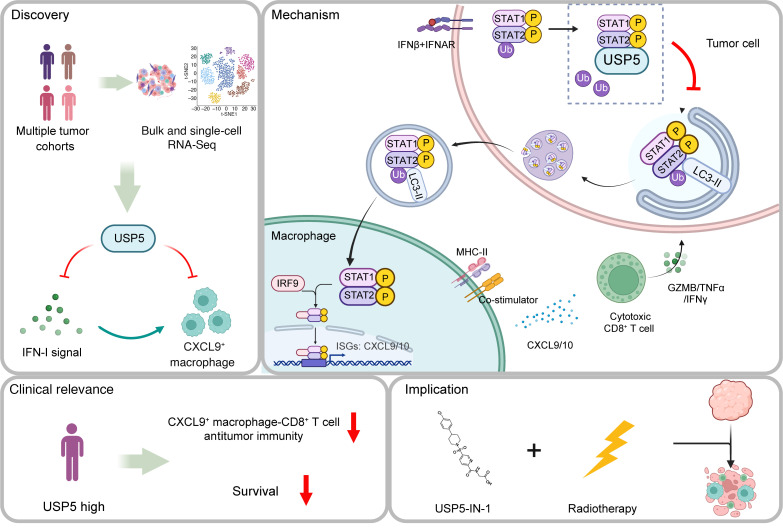
Proposed working model. By performing integrated analyses of bulk and single-cell RNA-Seq data of multiple human malignancies, we demonstrate that the deubiquitinase USP5 inhibits intratumoral IFN-I signaling–induced CXCL9^+^ macrophage–mediated antitumor immunity. Mechanistically, K63-linked ubiquitination at the K167 site of STAT2 facilitates LC3-dependent secretory autophagy of p-STAT1 and p-STAT2, thereby transferring tumor-intrinsic IFN-I signals to macrophages and inducing CXCL9^+^ macrophages to enhance CD8^+^ T antitumor immunity. However, this process is inhibited by USP5-mediated deubiquitination. Higher USP5 expression predicts worse survival in some patients with cancer, and preclinical studies show that pharmacological inhibition of USP5 enhances the efficacy of radiotherapy.

In summary, our study adds secretory autophagy–mediated intercellular IFN-I signaling delivery to the existing network of IFN mechanisms of action and regulation. The study also highlights the depletion of USP5 as a stimulus for the transfer of IFN-I signaling from tumor cells to macrophages, thus reprogramming macrophages to IFN-I–responsive cells with potent antitumor activity (Figure 9), which enhances radiotherapy efficacy. Pharmacological inhibition of USP5 may be a promising strategy for current cancer therapies.

## Methods

### Sex as a biological variable.

Our study examined male and female patients, and similar findings are reported for both sexes. Our study exclusively examined female mice. It is unknown whether the findings are relevant for male mice.

### Clinical specimens.

We collected 261 paraffin-embedded NPC samples from Sun Yat-sen University Cancer Center between August 2009 and September 2016; 31 NPC samples were used for IHC detection of USP5 protein expression and tumor immune infiltration analysis. The other 230 samples were used for IHC detection of USP5 protein expression and patient survival analysis. None of the patients had received any antitumor therapy before sampling. Tumor pathological types were classified according to the WHO classification, and tumor-node-metastasis (TNM) stages were classified based on the 7th edition of the American Joint Committee on Cancer (AJCC) Cancer Staging Manual. All patients received radical radiotherapy and platinum-based chemotherapy. The patients’ clinical characteristics are listed in Supplemental Table 2.

### Cell culture and treatment.

The human NPC cell lines SUNE1 and HK1 were provided by Mu-Sheng Zeng at Sun Yat-sen University Cancer Center. The human monocyte leukemia cells THP1, mouse monocyte-macrophage leukemia cells RAW264.7, B16 murine melanoma cells, and MC38 and CT26 murine colon adenocarcinoma cells were obtained from American Type Culture Collection (ATCC). All cell lines were cultured in RPMI-1640 or DMEM (Invitrogen) supplemented with 10% FBS (Gibco) and 100 U/mL penicillin-streptomycin (Gibco, 15140122). We knocked out the *USP5* (*Usp5*), *STAT2* (*Stat2*), or *Ifnar1* gene by CRISPR/Cas9 genome editing with the PX458 plasmid. The sequences of the small-guide RNAs are listed in Supplemental Table 5.

Cells were seeded in a culture plate 1 day before treatment with IFN-β or an inhibitor after complete adherence. Human NPC and murine tumor cells were respectively treated with human IFN-β (10 ng/mL; R&D Systems) or murine IFN-β (20 ng/mL; TargetMol) unless otherwise indicated. Cells were treated with MRT68921(2 μM; TargetMol), 3-MA (10 mM; TargetMol), CQ (50 μM; Sigma-Aldrich), bafilomycin A1 (10 nM; MedChemExpress), PYR-41 (10 μM; MedChemExpress), MLN4924 (1 μM; Selleck), and USP5-IN-1 (10–20 μM; TargetMol).

### Plasmids and transfection.

The USP5 coding sequence was cloned into the pSin-EF2-puro and PHAGE vectors to construct the following plasmids: pSin-EF2-puro-USP5-FLAG, pSin-EF2-puro-USP5-C335A-FLAG, and PHAGE-USP5-FLAG. The pCMV-STAT2(human)-FLAG-Neo and pCDH-CMV-STAT2(human)-Linker-EGFP-Myc-EF1a-Puro plasmids were purchased from MiaoLing Bio. The FLAG-tagged Y690A-, K167R-, K197R-, and K239R-STAT2 mutants were cloned into the pCMV vector to construct the relevant plasmids. PRK-HA-Ub and mutants were gifted by Bo Zhong (Wuhan University, Wuhan, Hubei, China). The shRNA sequences targeting ATG5 and ATG9 were obtained via shRNA sequence prediction website portal (Sigma-Adrich; https://www.sigmaaldrich.cn/CN/zh/semi-configurators/shrna? activeLink=productSearch) (Supplemental Table 5) and were then synthesized and inserted into the pLKO.1-RFP vector to construct the PLKO.1-shATG5 or PLKO.1-shATG9 plasmid. The LC3B-siRNA was designed by Beijing Tsingke Biotech, and the sequence is provided in Supplemental Table 5. Transfections were performed with Lipofectamine 3000 (Invitrogen), and the transfection efficiency was determined by RT-qPCR and Western blotting after 24–48 hours of transfection.

### Tumor growth, treatment, and analyses.

Six-week-old female, specific pathogen–free BALB/c mice and C57BL/6 mice were purchased from Charles River Laboratories (Zhejiang) and cohoused in the Animal Experiment Center of Sun Yat-sen University Cancer Center. The control and *Usp5*-depleted CT26 cells (1 × 10^6^) were subcutaneously inoculated into BALB/c mice. The control and *Usp5*-depleted MC38 (1 × 10^6^) or B16 (5 × 10^5^) cells were subcutaneously inoculated into C57BL/6 mice. On day 10 after cell inoculation, mice with a tumor mass were randomly grouped, anesthetized with isoflurane, and fixed. The tumors were locally irradiated with a single fraction (15 Gy) using an RS-200-PRO-225 Biological Irradiator; mouse bodies were covered with lead plates to protect them from radiation. The anti-IFNAR1 antibody (100 μg per mouse, on days 7 and 14; BioXcell, BE0241) and USP5-IN-1 (0.25 μg/g, every 2 days from day 7; TargetMol) were administered intraperitoneally. The tumors were monitored every 2 or 3 days. To monitor the adverse effects of USP5-IN-1 treatment, the body weight of mice was measured every 2 days. Peripheral blood was collected by orbital vein sampling for blood routine examination and serum biochemical test after 5 doses of USP5-IN-1.

Mice were euthanized by CO_2_ asphyxiation for tumor harvesting or once the tumor diameter was greater than 1.5 cm. The weights of the excised tumors were recorded. The tumor tissues were digested into single-cell suspensions, and flow cytometry was used for immune infiltration analysis. For CD8^+^ T cell cytotoxicity evaluation, single-cell suspensions were treated with Cell Stimulation Cocktail (plus protein transport inhibitors) (Thermo Fisher Scientific, 00-4975-93) for 4 hours before staining, and the percentages of GZMB^+^CD8^+^ and IFN-γ^+^TNF-α^+^CD8^+^ T cells were determined.

### CCK-8 assays.

For the CCK-8 assay, cells were irradiated and then seeded in 96-well plates. CCK-8 reagent (1:10 per well; TargetMol) was added and incubated at 37°C for 2 hours. Then, the absorbance value at 450 nm was measured at the indicated times.

### Macrophage generation and activation assays.

Human macrophages were generated by culturing THP1 cells in RPMI-1640 medium supplemented with 10% FBS and 100 ng/mL PMA for 2 days. THP1-MΦ were then cocultured with IFN-β–treated NPC cells or treated with LEVs from IFN-β–treated NPC cells for 48 hours. The murine RAW264.7 cells were cocultured with IFN-β–treated B16 or CT26 cells for 48 hours. The expression of HLA-DR and CD86 on human THP1-MΦ and CD40 expression on RAW264.7 cells was determined by flow cytometry. The relative mRNA expression of *ISG15*, *IFI44*, *CXCL9*, *CXCL10*, and *SPP1* in THP1-MΦ, and the relative mRNA expression of *Cxcl9* and *Cxcl10* in RAW264.7 cells, was measured by RT-qPCR.

### Flow cytometric analysis.

For tumor immune infiltration analysis, fixable viability dye eFluor 455UV or eFluor 780 (eBioscience, 65-0868-14 and 65-0865-14) was used to label dead cells. An intracellular fixation and permeabilization buffer set (eBioscience, 88-8824-00) was used for intracellular staining, and FcR-blocking reagents (BioLegend) were used to avoid nonspecific binding according to the manufacturer’s instructions. Cells were incubated with the indicated antibodies on ice for 30 minutes in a staining buffer, washed twice, and suspended in a flow staining buffer. All data were obtained with a CytoFLEX flow cytometer (Beckman Coulter), and the results were analyzed using FlowJo software (BD Biosciences). The antibodies used are listed in Supplemental Table 4.

### RT-qPCR.

Total RNA was extracted using TRIzol reagent (Invitrogen). HiScript III RT SuperMix for qPCR (gDNA wiper) (Vazyme, R323-01) was used to synthesize the complementary DNA. Real-time quantitative PCR with ChamQ SYBR qPCR Master Mix (Vazyme, Q311-03) on a CFX96 Touch sequence detection system (Bio-Rad) or a LightCycler 480 System (Roche) was used to evaluate the relative gene expression with the calculation in the 2^–ΔΔCt^ method. *GAPDH* expression was used as the internal control. The primer sequences are listed in Supplemental Table 5.

### Western blot analysis.

Cells were lysed with 1× RIPA lysis buffer (Millipore) supplemented with protease and phosphatase inhibitors (Roche). The nuclear and cytoplasmic proteins were extracted with NE-PER nuclear and cytoplasmic extraction reagents (Thermo Fisher Scientific). Total protein was denatured by heating in 0.1% SDS loading buffer, separated by SDS-PAGE, and transferred to PVDF membranes (Millipore). The membranes were blocked with 5% BSA and incubated first with primary antibodies and then with HRP-conjugated secondary antibodies. The antibodies used are listed in Supplemental Table 4, and the unprocessed scans of the immunoblots are provided.

### IHC analyses.

Multiplex IHC was performed using a PANO 7-plex IHC kit (Panovue) according to the manufacturer’s instructions. Tumor-infiltrating CD68^+^ macrophages, CXCL9^+^ cells, CD8^+^ T cells, and GZMB^+^CD8^+^ T cells in human NPC tissue and CXCL9^+^CD11b^+^ macrophages in murine tumors were analyzed using the HALO image analysis platform. USP5 IHC staining was analyzed, and staining intensity values were calculated as reported in our previous study (49). USP5 staining intensity was scored as follows: 0 (negative), 1 (weak), 2 (moderate), and 3 (strong). The percentage of positive tumor cells was scored as follows: 1 (<25%), 2 (25%–50%), 3 (50%–75%), and 4 (≥75%). The scores were calculated as the product of the staining intensity score and the score of percentage of positive tumor cells. For instance, if 70% of tumor cells are USP5^+^ and the staining intensity is strong, the USP5 staining score will be 3 multiplied by 3, which equals 9. The USP5 staining scores and numbers of cases in 230 NPC tumors are as follows: 0 (22 cases), 1 (27 cases), 2 (46 cases), 3 (36 cases), 4 (28 cases), 6 (29 cases), 8 (26 cases), 9 (7 cases), and 12 (9 cases). NPCs with a score of less than 6 were defined as tumors with low expression of USP5 (159 out of 230), and the rest were defined as tumors with high expression of USP5 (71 out of 230). The scoring was conducted without awareness of the clinical information of the NPC cases. The antibodies used are listed in Supplemental Table 4.

### Immunofluorescence analysis.

Cells were fixed with 4% paraformaldehyde and permeabilized with 0.1% Tween 20 and 0.5% Triton X-100. Then, the cells were blocked with QuickBlock blocking buffer for Immunol Staining (Beyotime, P0260) and incubated with primary antibodies overnight at 4°C and Alexa Fluor secondary antibodies for 1 hour at room temperature. The antibodies used are listed in Supplemental Table 4. The cells were then stained with Hoechst. Images were obtained with an LSM 980 confocal laser scanning microscope operated with ZEN software (Zeiss). The fluorescence intensity and colocalization were analyzed in ImageJ (NIH) software, and Manders’ colocalization coefficients were calculated in at least 40 cells from 10 or more randomly selected fields of view.

### LEV isolation.

LEVs were purified according to a standard differential centrifugation protocol (18, 54). Briefly, cells seeded in 15 cm culture dishes at approximately 70% confluence were incubated with serum-free DMEM and IFN-β for 48 hours. The culture medium was collected and centrifuged serially at 2,000*g* for 20 minutes to pellet cellular debris and apoptotic bodies, 10,000*g* for 60 minutes to pellet LEVs, and 100,000*g* in an ultracentrifuge for 2 hours to pellet exosomes. The LEV pellets were gently washed twice with cold PBS. LEVs from different cells or experimental conditions were corrected based on total cell number to ensure that LEV or LEV protein quantification was not confounded by seeding differences.

### Co-IP and mass spectrometry analysis.

Indicated SUNE1 cells were lysed on ice with IP lysis buffer supplemented with protease and phosphatase inhibitors. Cell lysates were immunoprecipitated with the indicated antibodies overnight at 4°C, and immune complexes were captured by Pierce Protein A/G Magnetic Beads (Thermo Fisher Scientific). The eluates were then separated by SDS-PAGE and stained with a fast silver stain kit (Beyotime, P0017S) or Coomassie blue super-fast staining solution (Beyotime, P0003S). Mass spectrometry analysis investigating the STAT2-interacting proteins and STAT2 ubiquitinated sites was conducted by NuoBiotech and Wininnovate Biotechnology, respectively. The proteins of interest in the co-IP products were verified by Western blotting. The ubiquitin assay was conducted under denaturing conditions as previously described (55, 56). The antibodies used are listed in Supplemental Table 4.

### Bioinformatics analysis.

We determined the IFN-I response of 2 cohorts of NPC tumors and 18 other malignancies in TCGA datasets, based on their bulk tumor RNA-Seq data, by generating an IRS score that reflects the IFN-I response for each sample. The score was calculated by the single-sample GSEA (ssGSEA), and the gene set GOBP_RESPONSE_ TO_TYPE_I_ INTERFERON was used. Genes that are highly expressed in tumors with low (bottom 25% vs. top 25%) IRS were identified (log_2_FC ≥ 1.2, FDR < 0.01).

We then used our previously published scRNA-Seq data of NPC (22) to perform a monocyte and macrophage subpopulation analysis by unsupervised clustering and subsequently divided the macrophages into *CXCL9^hi^*, *SPP1^hi^*, and *CXCL9^lo^SPP1^lo^* subgroups. The AddModuleScore function of Seurat was applied to score the curated gene signature in each cell subgroup. Genes that were highly expressed in each of the 3 subgroups were identified (FC > 1.5, adjusted *P* < 0.01). Furthermore, 16 genes highly expressed in *CXCL9^hi^* macrophages were selected as a gene set to evaluate the abundance of *CXCL9^hi^* macrophages in NPC, HNSC, SKCM, and COAD with ssGSEA. CIBERSORTx and TIMER2.0 were used to estimate infiltrating immune cells in tumors (57, 58). The relationship between IRS, *USP5* expression, *CXCL9^hi^* macrophages, and intratumoral CD8^+^ T cells was then investigated. The *USP5* expression levels in different cell types in NPC were investigated using the scRNA-Seq data. Similar research was conducted using public scRNA-Seq data of melanoma and colorectal carcinoma, and the results were visualized by Single Cell Portal (https://singlecell.broadinstitute.org).

### Statistics and reproducibility.

Data are presented as the mean ± SD of at least 3 independent experiments. Statistical analyses were performed using SPSS version 25 (IBM) or GraphPad Prism. Two-tailed unpaired Student’s *t* test and 1-way or 2-way ANOVA with Bonferroni’s test for multiple comparisons were used to calculate *P* values. The clinical characteristics of patients with NPC were compared with the χ^2^ test. Time-to-event data were described using Kaplan-Meier curves, and differences in survival were determined using the log-rank test. The independent prognostic factors were evaluated by a multivariate Cox proportional hazards model. A *P* value of less than 0.05 was considered statistically significant.

### Study approval.

The IRBs of Sun Yat-sen University Cancer Center approved this study (G2024-168-01), in which anonymized data were analyzed, and waived the requirement for informed consent. All animal experiments were approved by the Experimental Animal Ethics Committee of Sun Yat-sen University Cancer Center (L025501202208015).

### Data availability.

The NPC sequencing data used in this study were previously published in NCBI’s Gene Expression Omnibus (GEO GSE150430, GSE102349, and CNP0001503). The sequencing and clinical data of HNSC, SKCM, COAD, and other cancers were obtained from TCGA. Original data for this study have been uploaded onto the Research Data Deposit public platform (www.researchdata.org.cn) under RDDB2025949013 and can be obtained from the corresponding author. Values for all data points in graphs are reported in the Supporting Data Values file.

## Author contributions

The order of the co–first authors was determined based on the significance, time, and effort each author invested in the project. JYL and NL designed the experiments. JYL, YQL, and JHD carried out most of the experiments and analyzed the resulting data. SG, QMH, and JWB collected NPC samples and performed the IHC experiments. XRT, SWH, and XYL helped with the data analyses. YQL, YFD, and SYF helped to perform the bioinformatics analysis. JYL and NL wrote and revised the manuscript. NL, RG, and JM conceived and supervised the study and provided funding and scientific direction. All authors reviewed and discussed the final version of the paper.

## Funding support

This work was supported by the following grants:

- National Natural Science Foundation of China (82403970 to JYL, 82172806 to NL).
- China Postdoctoral Science Foundation (2023TQ0389 and GZB20230895 to JYL).
- Young Talents Program of Sun Yat-sen University Cancer Center (YTP-SYSUCC-0010 to NL).
- Cancer Innovative Research Program of Sun Yat-sen University Cancer Center (CIRP-SYSUCC-0005 to JM).

## Supplementary Material

Supplemental data

Unedited blot and gel images

Supporting data values

## Version 1 12/01/2025

Electronic publication
