# Supplementary material for: LC3-dependent intercellular transfer of phosphorylated STAT1/2 elicits CXCL9+ macrophages and enhances radiation-induced antitumor immunity
Source: J Clin Invest. 2025 Dec 1;135(23):e195279. doi: 10.1172/JCI195279 (PMC12646668; doi:10.1172/JCI195279)
Supplement: Supplemental data [file jci-135-195279-s126.pdf]

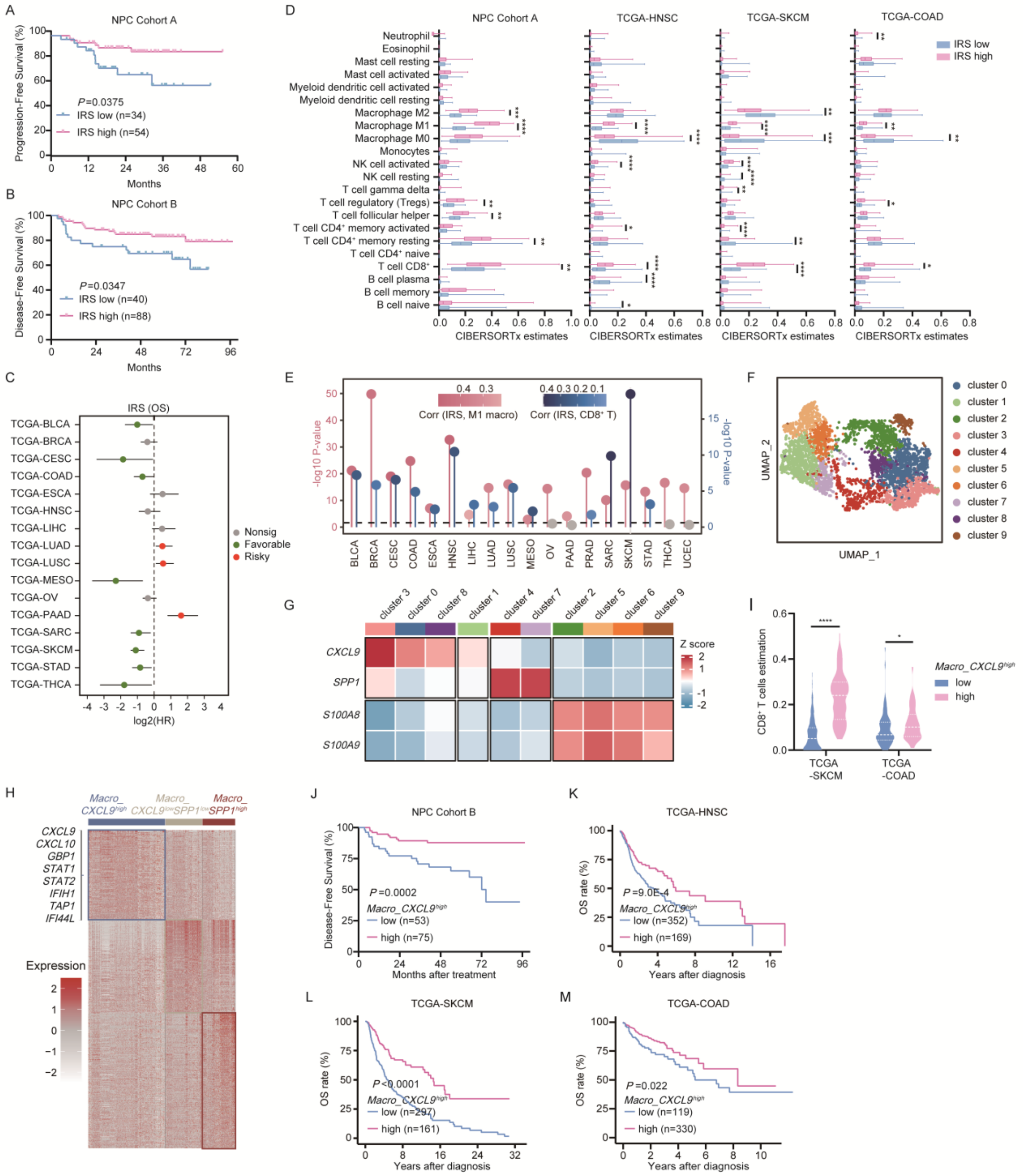

Supplemental Figure 1 (Extended data related to Main Figure 1)

(**A** and **B**) Kaplan–Meier survival curves for NPC patients in two different cohorts with high or low IFN-I response gene scores (IRS) (log-rank test). (**C**) Forest plot showing the prognostic value of IRS for overall survival in patients with different kinds of cancers in TCGA datasets. (**D**) The CIBERSORTx analysis result of NPC, HNSC, SKCM, and COAD with high or low IRS (multiple t-tests with Bonferroni's correction). (**E**) Lollipop diagram showing the positive correlation between IRS with M1 macrophages or CD8$^+$ T cells in

different kinds of cancers in the TCGA database. (**F**) Uniform manifold approximation and projection (UMAP) visualization of minor monocyte and macrophage clusters from single-cell transcriptomes (3751 cells) of 15 NPC samples. (**G**) Heatmap showing the relative expression of *CXCL9*, *SPP1*, *S100A8,* and *S100A9* between the monocyte and macrophage subsets. (**H**) Heatmap showing the highly expressed genes (FC > 1.5, *P* < 0,01) in the $CXCL9^{high}$, $SPP1^{high}$, and $CXCL9^{low}SPP1^{low}$ macrophage subpopulations. IFN-stimulated genes highly expressed in $CXCL9^{high}$_Macrophages are shown. (**I**) CIBERSORTx analyses of $CD8^+$ T cells in SKCM (n = 118 in each group) and COAD (n = 94 in each group) tumors with high or low intratumoral $CXCL9^{high}$ macrophages (multiple t-tests with Bonferroni's correction). (**J-M**) Kaplan–Meier survival curves for NPC (**J**), HNSC (**K**), SKCM (**L**), and COAD (**M**) tumor patients with high or low intratumoral Macro_$CXCL9^{high}$ (log-rank test). *P < 0.05, **P < 0.01, ***P < 0.001 and ****P < 0.0001.

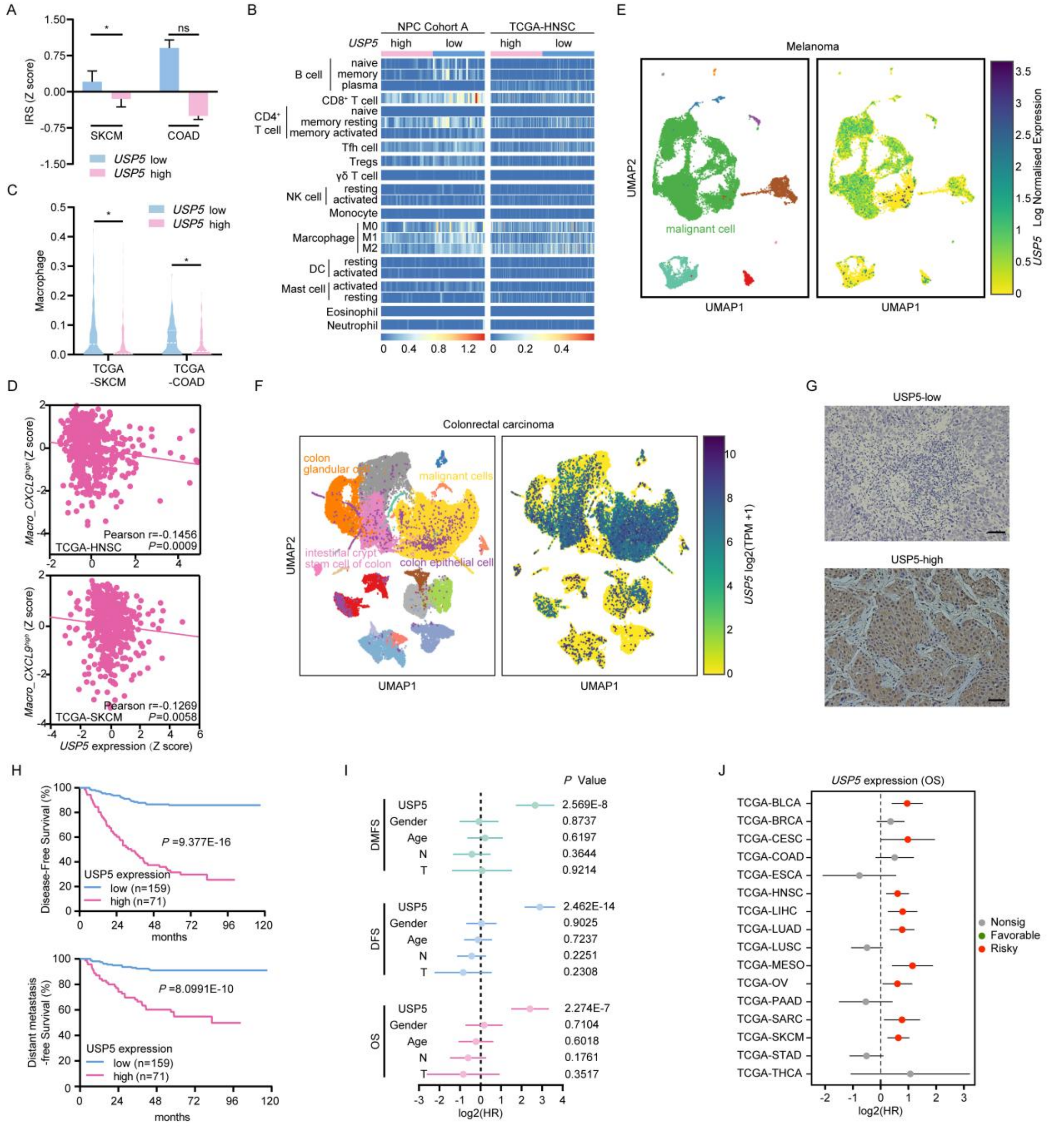

Supplemental Figure 2 (Extended data related to Main Figure 1)

(**A**) IRS in SKCM (n=118 in each group) and COAD (n=94 in each group) with high or low *USP5* expression. (**B**) Heatmap showing the CIBERSORTx analyses results of NPC (n=28 in each group) and HNSC (n=129 in each group) with high or low *USP5* expression. (**C**) Macrophage estimation in SKCM (n=118 in each group) and COAD (n=94 in each group) with high or low *USP5* expression. (**D**) Pearson correlation analyses (two-tailed) of *USP5* expression and CXCL9^{high} macrophages in HNSC and SKCM. (**E** and **F**) UMAP diagrams showing *USP5* expression in different cell types in human melanoma and colorectal carcinoma.

(**G**) Representative images of NPC with low or high USP5 protein expression. Scale bar, 100 μm. (**H**) Kaplan–Meier analyses of disease-free and distant metastasis-free survivals based on the USP5 expression level (log-rank test). (**I**) Forest plots showing the results of multivariate Cox regression analyses indicated that USP5 expression is a significant prognostic indicator for overall, disease-free, and distant metastasis-free survivals in NPC patients (Cox proportional-hazards model). (**J**) Forest plot showing the prognostic value of *USP5* expression for overall survival in patients with different kinds of cancers in the TCGA database. The data are presented as the mean ± SD, and comparisons were performed using t-tests (**A** and **C**). *P < 0.05.

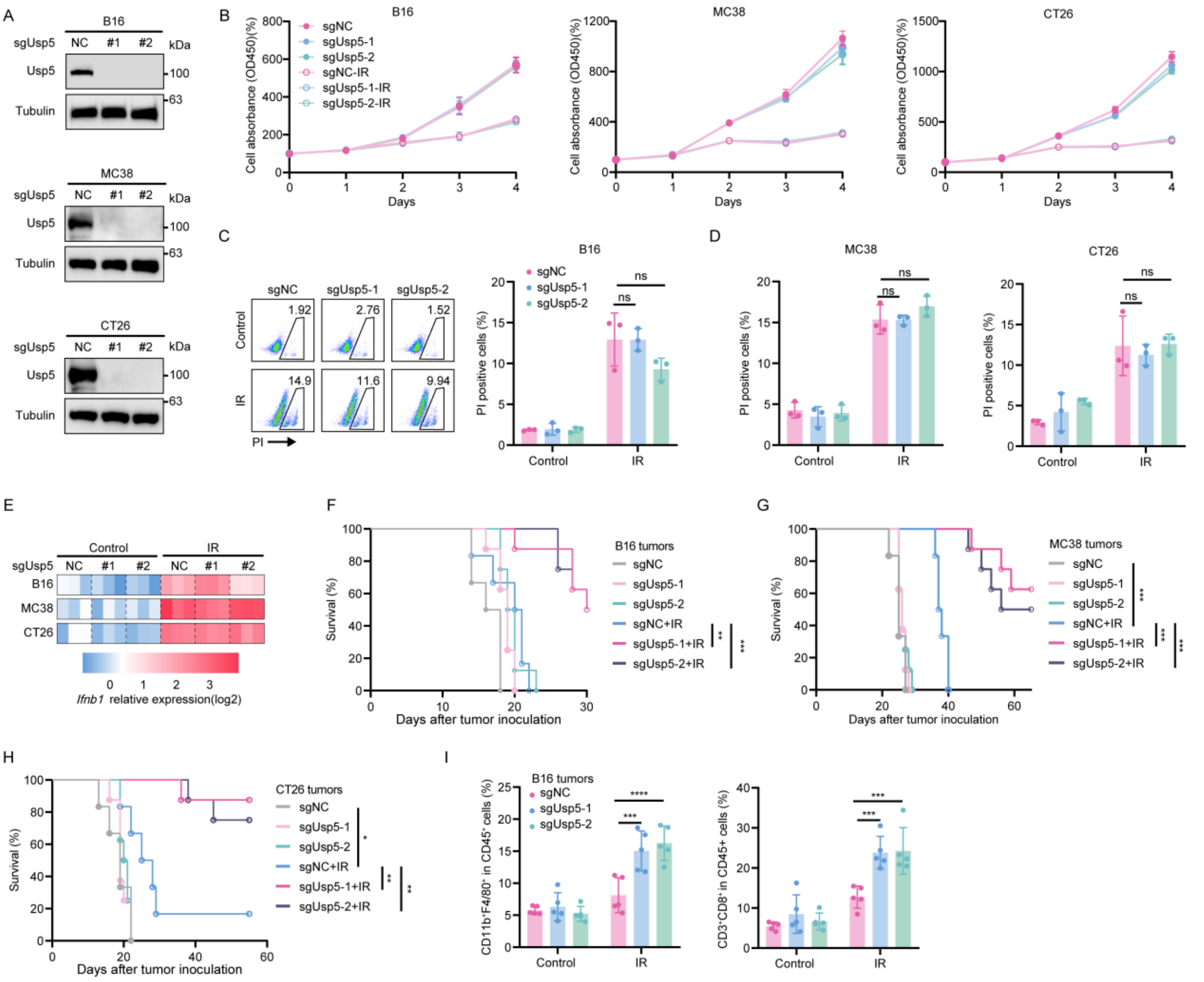

Supplemental Figure 3 (Extended data related to Main Figure 2)

(**A**) The Usp5 protein levels in control and Usp5 knockout B16, MC38, and CT26 cells. (**B**) Cell growth rates of WT or USP5-deficient B16, MC38, and CT26 cells with or without IR examined by CCK-8 assays. (**C** and **D**) Percentage of Propidium Iodide (PI) positive cells in control and Usp5-depleted B16, MC38, and CT26 cells with or without IR. (**E**) Heatmap showing the relative *Ifnb1* mRNA expression in B16, MC38, and CT26 tumor cells with or without IR. (**F-H**) The survival of B16 (**F**), MC38(**G**), or CT26 (**H**) tumor-bearing mice with indicated treatments (n=6 in sgNC groups and n=8 in sgUsp5 groups, log-rank test). (**I**) Flow cytometric results showing the increased tumor-infiltrating CD11b^{+} F4/80^{+} macrophages and CD3^{+}CD8^{+} T cells in Usp5-depleted B16 tumors after IR (n=5 in each group). The results are representative of three independent experiments (**A-E**). The data are presented as the mean ± SD, and comparisons were performed using two-way ANOVA with Bonferroni's test for

multiple comparisons (**B-D,** and **I**). *$P < 0.05$, **$P < 0.01$, ***$P < 0.001$ and ****$P < 0.0001$.

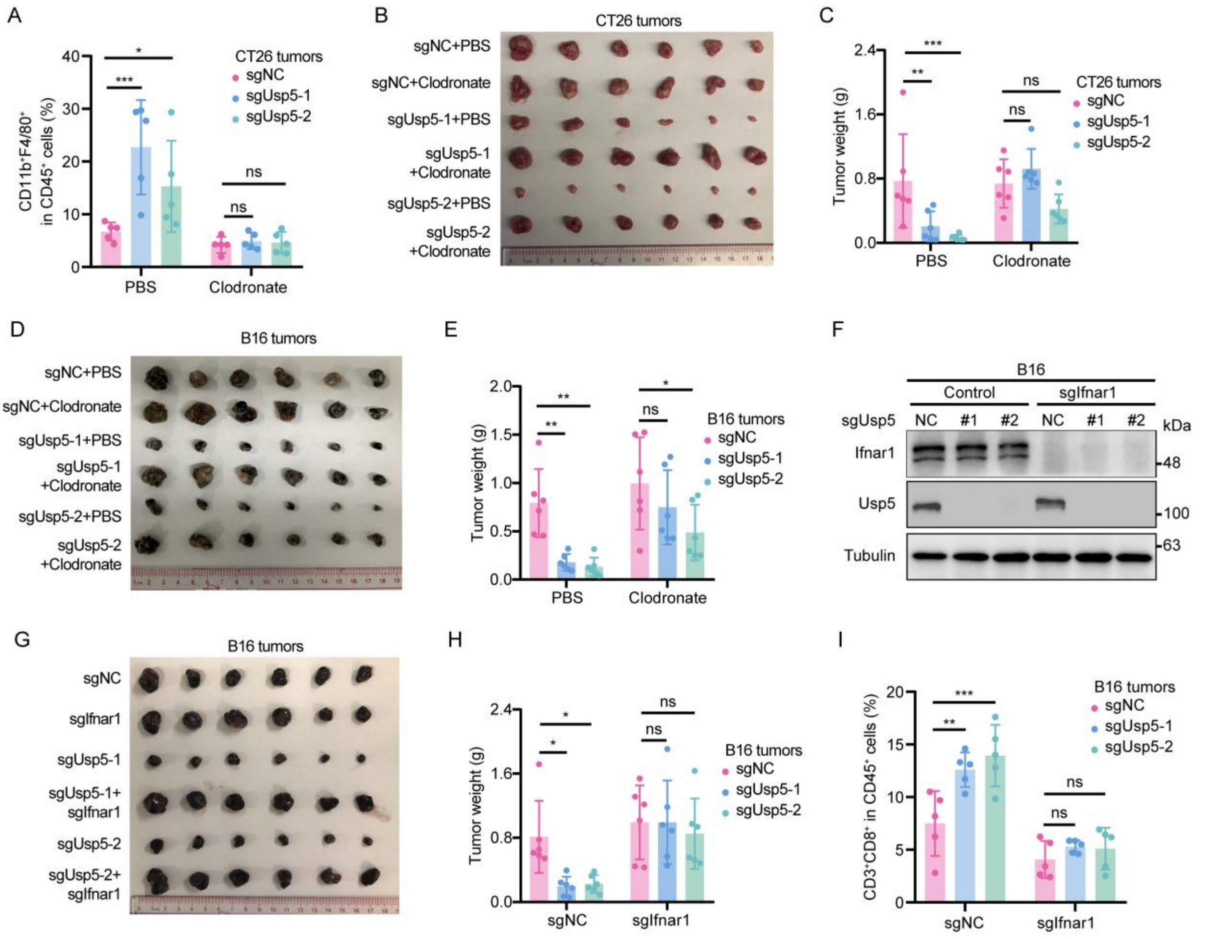

Supplemental Figure 4 (Extended data related to Main Figure 3)

(**A**) Flow cytometry confirmed the decreased tumor-infiltrating CD11b$^+$F4/80$^+$ macrophages in Usp5-depleted B16 tumors after Clodronate treatment (n=5 in each group). (**B-E**). Images and tumor weights of CT26 tumors (**B** and **C**) and B16 tumors (**D** and **E**) with indicated treatments (n=6 in each group). (**F**) The Ifnar1 protein levels in control and Ifnar1 knockout B16 cells. (**G** and **H**) The image and tumor weights of control and Ifnar1-depleted B16 tumors (n=6 in each group). (**I**) Flow cytometric results show the reduced tumor-infiltrating CD3$^+$CD8$^+$ T cells in Ifnar1-depleted B16 tumors after IR (n=5 in each group). The data are presented as the mean ± SD, and comparisons were performed using two-way ANOVA with Bonferroni's test for multiple comparisons (**A**, **C,** **E**, **H,** and **I**). *$P < 0.05$, **$P < 0.01$ and ***$P < 0.001$.

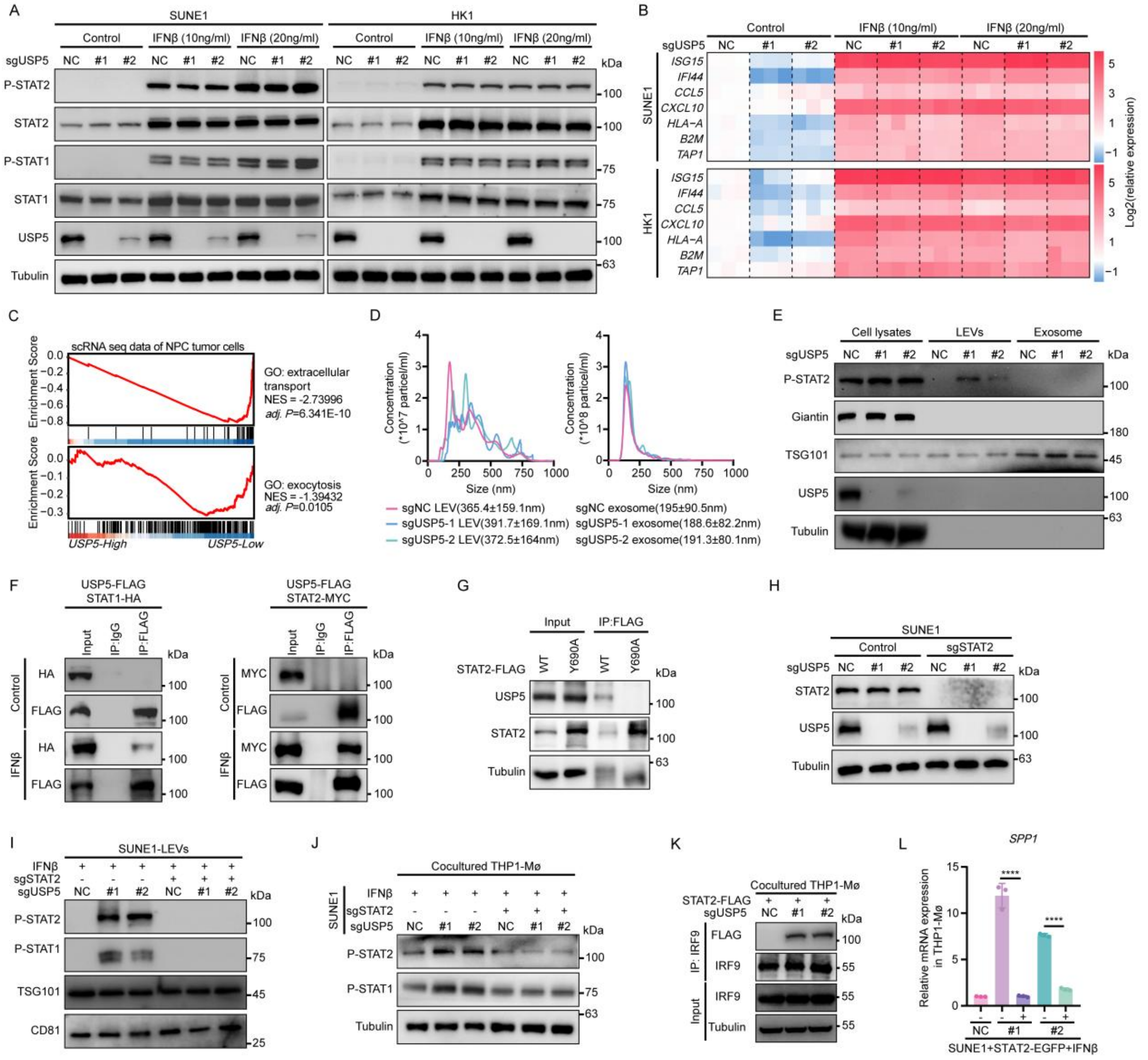

Supplemental Figure 5 (Extended data related to Main Figure 4)

(**A**) The total and phosphorylated STAT2/STAT1 protein levels in control and IFNβ-treated SUNE1 and HK1 cells. (**B**) Heatmap showing the relative *ISG15*, *IFI44*, *CCL5*, *CXCL10*, *HLA-A*, *B2M,* and *TAP1* mRNA expression in control and IFNβ-treated SUNE1 and HK1 cells. (**C**) The enriched extracellular transport and exocytosis pathways in *USP5*-low-expressed NPC cells. (**D**) Representative histograms of a NanoSight nanoparticle tracking analysis of large extracellular vesicles (LEVs) and exosomes secreted by IFNβ-treated control and USP5-depleted SUNE1 cells. (**E**) The P-STAT2 protein levels in cell lysate, LEVs, and exosomes from the culture medium of SUNE1 cells. **(F)** Co-IP showing interaction between USP5-FLAG and STAT1-HA or STAT2-MYC in control and IFNβ-treated SUNE1 cells. (**G**) Co-IP showing interaction between USP5 and FLAG-tagged STAT2-WT or STAT2-Y690A mutant.

(**H**) The STAT2 protein levels in control and STAT2-depleted SUNE1 cells. (**I**) The P-STAT1 and P-STAT2 levels in LEVs from control and STAT2-depleted SUNE1 cells. (**J**) WB shows decreased P-STAT1 and P-STAT2 levels in THP1-Mø cocultured with IFNβ-treated control and STAT2-depleted SUNE1 cells. (**K**) The interaction between THP1-Mø IRF9 and SUNE1 cells-derived FLAG-tagged STAT2. (**L**) The relative *SPP1* expression in $EGFP^+$ and $EGFP^-$ macrophages (two-way ANOVA with Bonferroni's correction). The results are representative of three independent experiments. ****$P < 0.0001$.

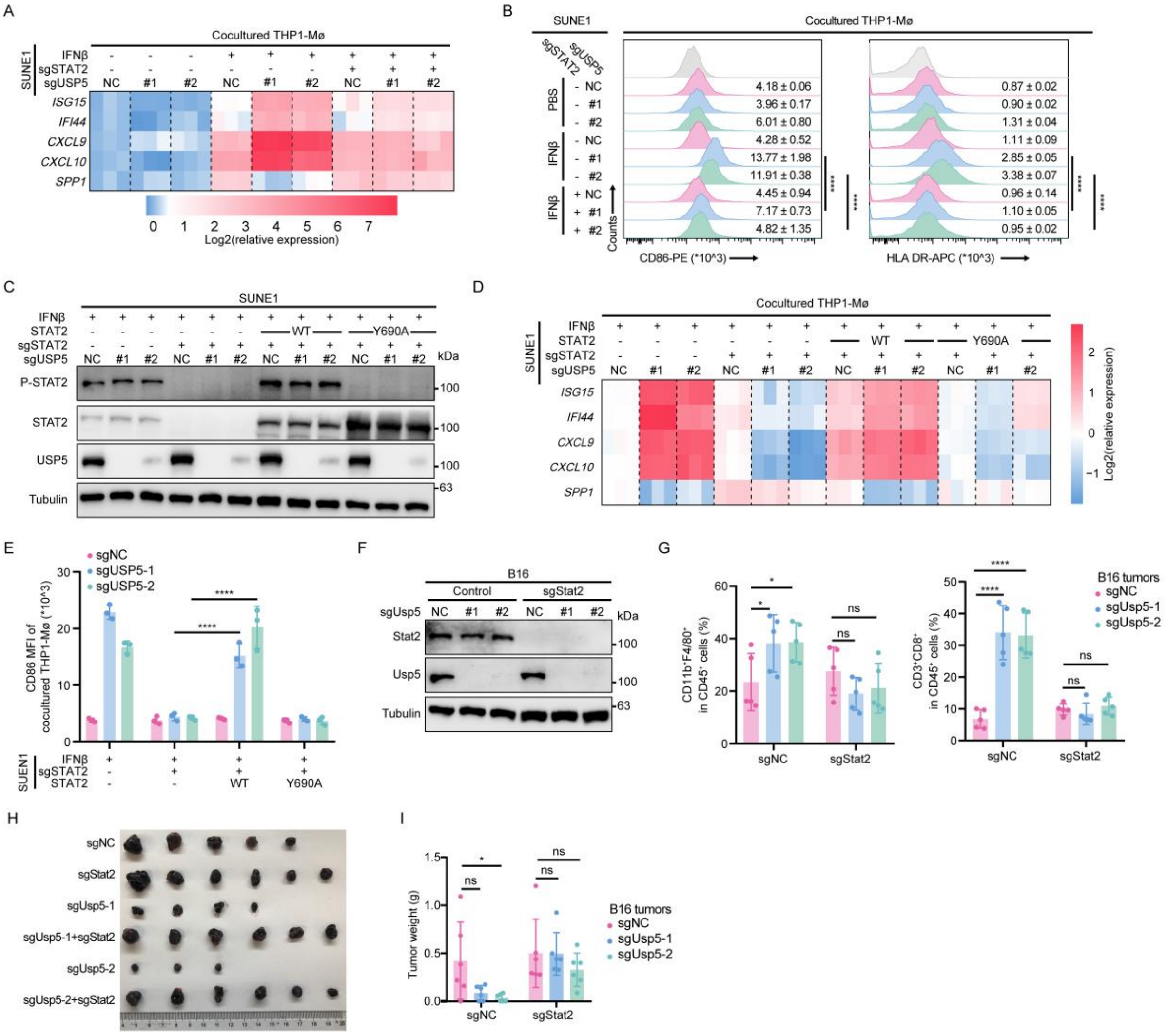

Supplemental Figure 6 (Extended data related to Main Figure 4)

(**A**) Heatmap showing the relative *ISG15*, *IFI44*, *CXCL9*, *CXCL10,* and *SPP1* mRNA expression in THP1-Mø after cocultured with indicated SUNE1 cells. (**B**) Histogram showing the surface CD86 and HLA-DR expression on THP1-Mø after cocultured with indicated SUNE1 cells. (**C**) The P-STAT2 and STAT2 protein levels in SUNE1 transfected with STAT2-WT or STAT2-Y690A mutant after IFNβ treatment. (**D**) Heatmap showing the relative *ISG15*, *IFI44*, *CXCL9*, *CXCL10,* and *SPP1* mRNA expression in THP1-Mø after cocultured with SUNE1 cells transfected with STAT2-WT or STAT2-Y690A mutant after IFNβ treatment. (**E**) The CD86 level on THP1-Mø after cocultured with SUNE1 cells transfected with STAT2-WT or STAT2-Y690A mutant after IFNβ treatment. (**F**) The Stat2 protein levels in control and Stat2-depleted B16 cells. (**G**) Flow cytometric results showed decreased tumor-infiltrating CD11b^{+}F4/80^{+} macrophages and CD3^{+}CD8^{+} T cells in Stat2-depleted B16 tumors after IR

(n=5 in each group). (**H** and **I**) The image and tumor weights of control and Stat2-depleted B16 tumors (n=6 in each group). The results are representative of three independent experiments (**A**-**F**). The data are presented as the mean ± SD. Comparisons were performed using two-way ANOVA with Bonferroni's test for multiple comparisons (**B**, **E**, **G,** and **I**). $*P < 0.05$ and $****P < 0.0001$.

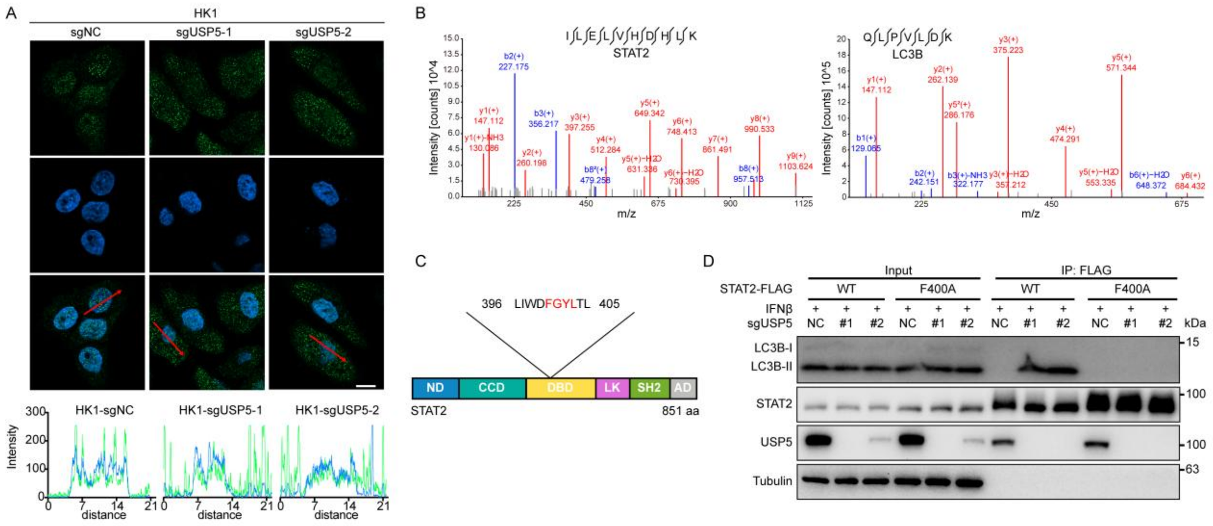

Supplemental Figure 7 (Extended data related to Main Figure 5)

(**A**) Representative confocal microscopy images and line scan analyses showing the different localization of P-STAT2 in WT or USP5-deficient HK1 cells. Scale bar, 10 μm. (**B**) The STAT2 and LC3B peptides were identified by immunoprecipitation with an anti-STAT2 antibody in USP5-depleted SUNE1 cells. (**C**) A domain map and the primary LC3-interaction region in STAT2. (**D**) Co-IP showed the impaired interaction between FLAG-tagged STAT2-F400A mutant and LC3B-II in IFNβ-treated USP5-depleted SUNE1 cells. The results are representative of three independent experiments (**A** and **D**).

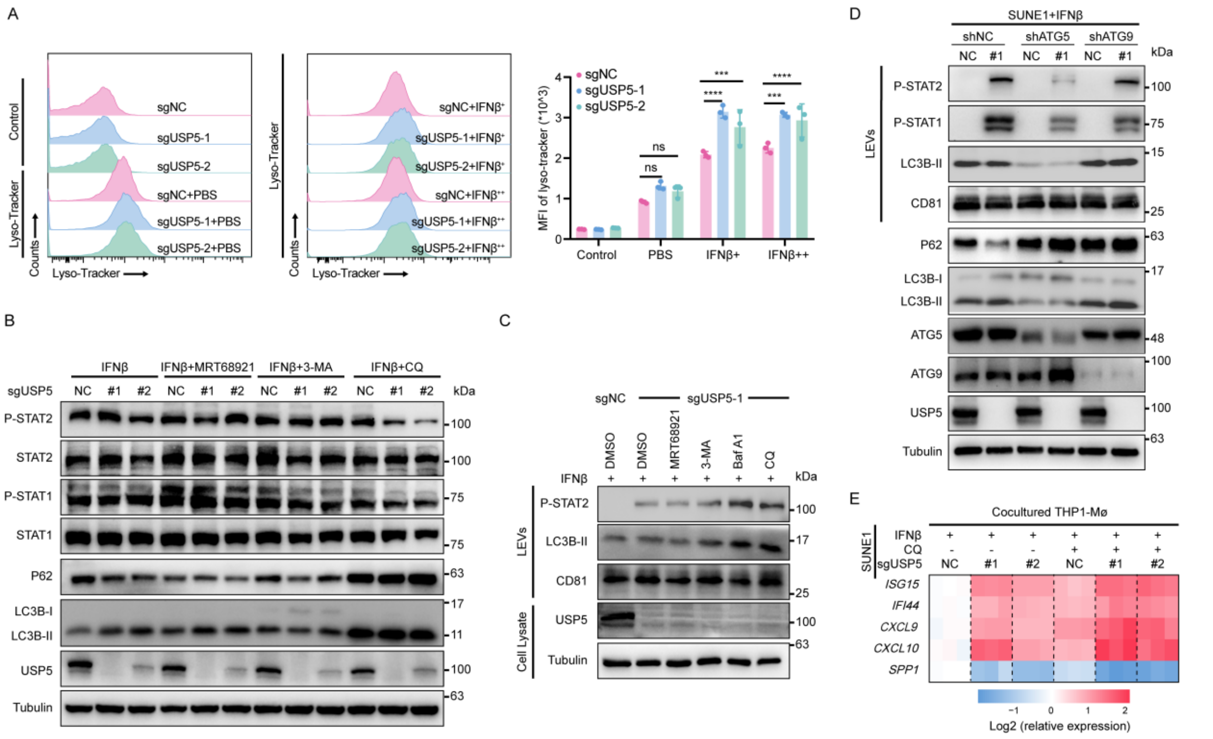

Supplemental Figure 8 (Extended data related to Main Figure 5)

(**A**) Flow cytometric results and histograms showing the lysosome function in control and USP5-depleted SUNE1 cells after IFNβ treatment. (**B**) The total and phosphorylated STAT2/ STAT1 protein levels in SUNE1 cells treated with IFNβ together with MRT68921, 3-MA, or CQ. (**C**) The P-STAT2 and P-STAT1 protein levels in large extracellular vesicles (LEVs) from USP5-depleted SUNE1 cells treated with IFNβ together with DMSO, MRT68921, 3-MA, Baf A1, or CQ. (**D**) The P-STAT2 and P-STAT1 protein levels in LEVs from ATG5- or ATG9-knockdown SUNE1 cells after IFNβ treatment. (**E**) Heatmap showing the relative *ISG15*, *IFI44*, *CXCL9*, *CXCL10,* and *SPP1* mRNA expression in THP1-Mø after cocultured with indicated SUNE1 cells. The results are representative of three independent experiments (**A**-**E**). The data are presented as the mean ± SD, and the comparisons were performed using two-way ANOVA with Bonferroni's test for multiple comparisons (**A**). ***$P < 0.001$ and ****$P < 0.0001$.

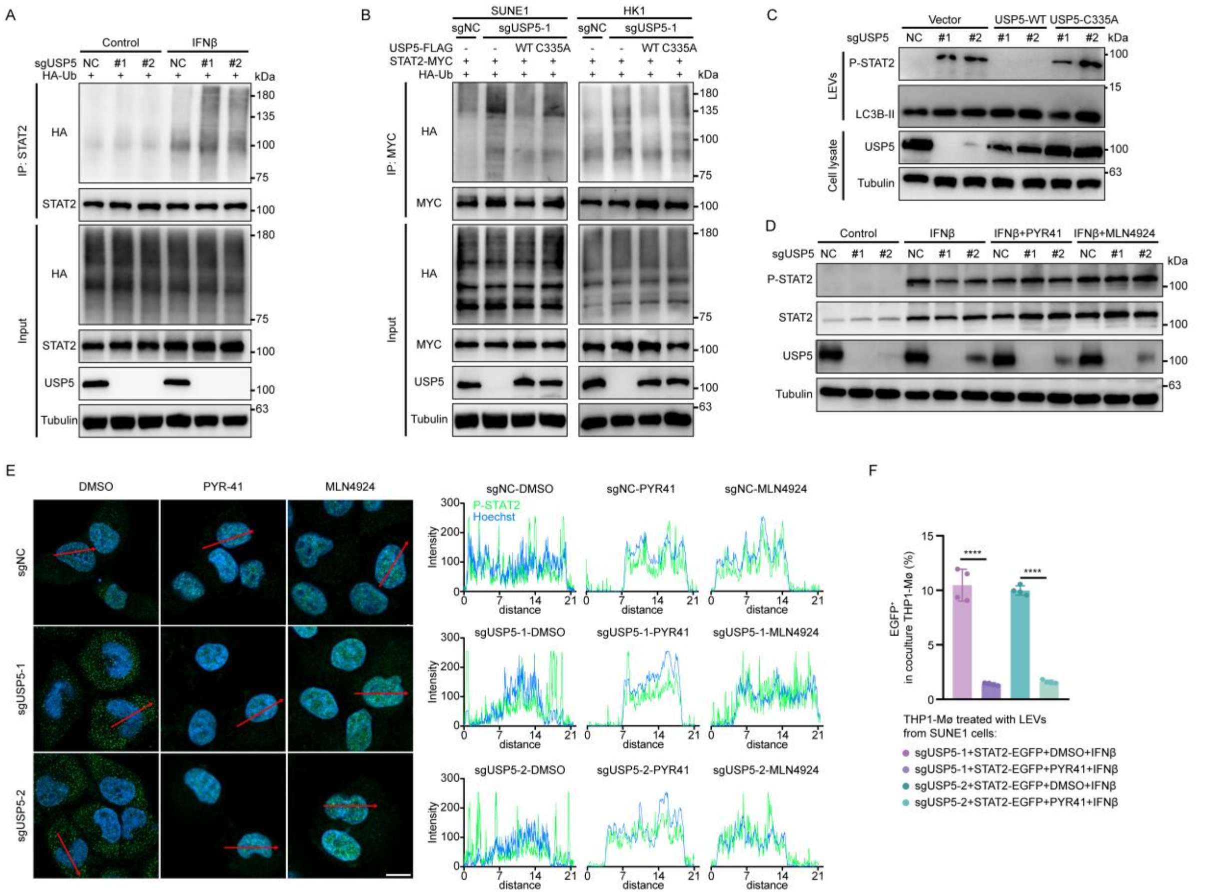

Supplemental Figure 9 (Extended data related to Main Figure 6)

(**A**) WB showing the increased ubiquitination of STAT2 in USP5-depleted HK1 cells after IFNβ treatment. (**B**) WB showing the decreased ubiquitination of STAT2 by overexpressing the WT USP5 in SUNE1 and HK1 cells. (**C**) The P-STAT2 proteins in large extracellular vesicles (LEVs) from SUNE1 cells with USP5-WT or -C335A mutant overexpression. (**D**) The total and phosphorylated STAT2 levels in SUNE1 cells treated with IFNβ together with PYR41 or MLN4924. (**E**) Representative confocal microscopy images showing the localization of P-STAT2 in SUNE1 and HK1 cells after PYR41 or MLN4924 treatment. Line scan analysis results of fluorescence intensity along the indicated lines are shown. Scale bar, 10 μm. (**F**) The percentage of $EGFP^{+}$ cells in THP1-Mø after being treated with LEVs from indicated SUNE1 cells. The results are representative of three independent experiments. The data are presented as the mean ± SD, and the comparisons were performed using two-way ANOVA with Bonferroni's test for multiple comparisons (**F**). ****$P < 0.0001$.

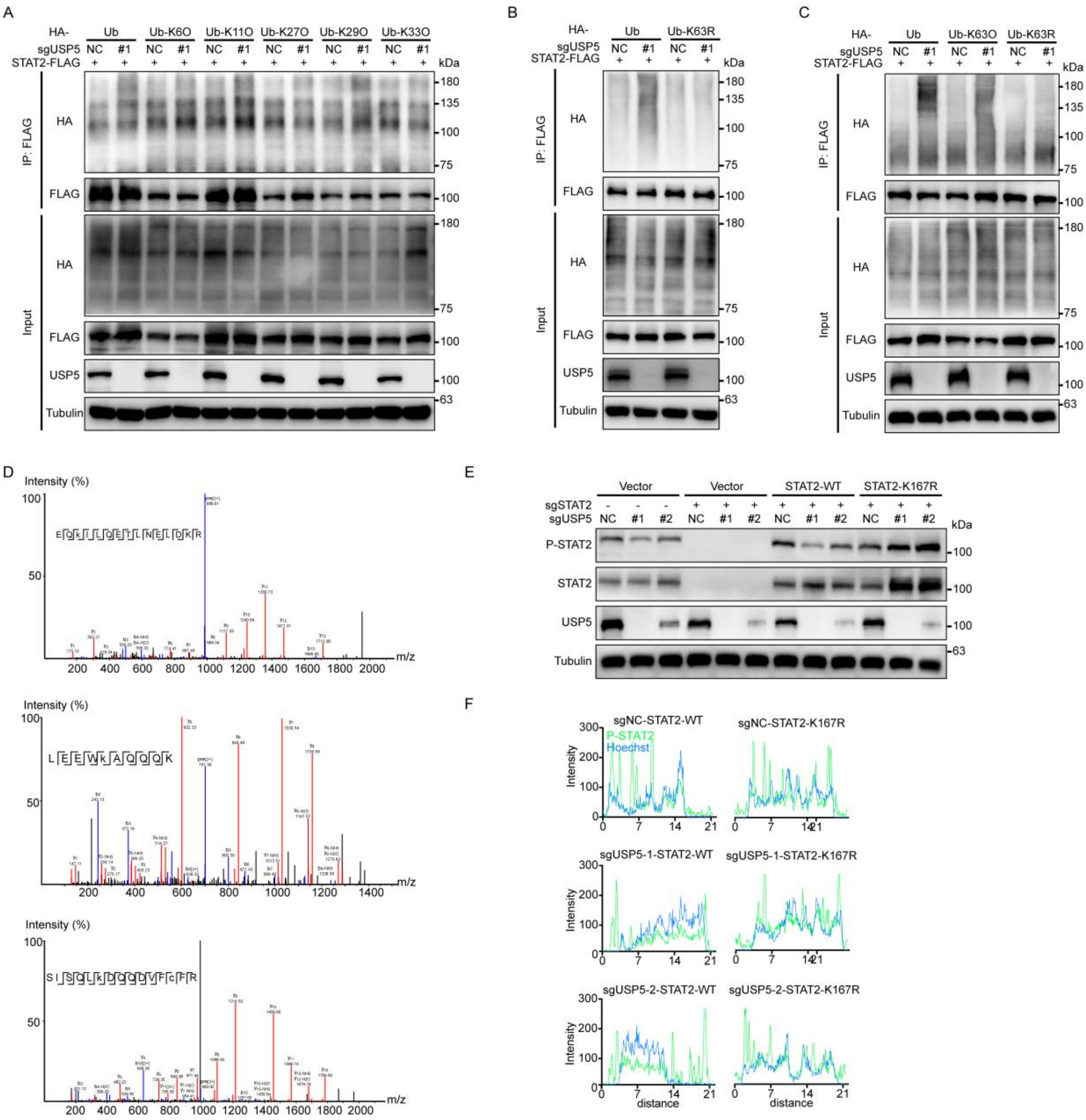

Supplemental Figure 10 (Extended data related to Main Figure 6)

(**A-C**) WB showing that USP5 depletion increases the K63-link ubiquitination of STAT2 in SUNE1 (**A** and **B**) and HK1 (**C**) cells. (**D**) Mass spectrometry (MS) analysis revealing three potential ubiquitination sites in STAT2-FLAG. (**E**) The P-STAT2 and STAT2 protein levels in SUNE1 transfected with STAT2-WT or STAT2-K167R mutant after IFNβ treatment. (**F**) The line scan analysis shows the different localization of phosphorylated WT or K167R-mutated STAT2 in WT or USP5-deficient SUNE1 cells. The results are representative of three independent experiments (**A-C, E,** and **F**).

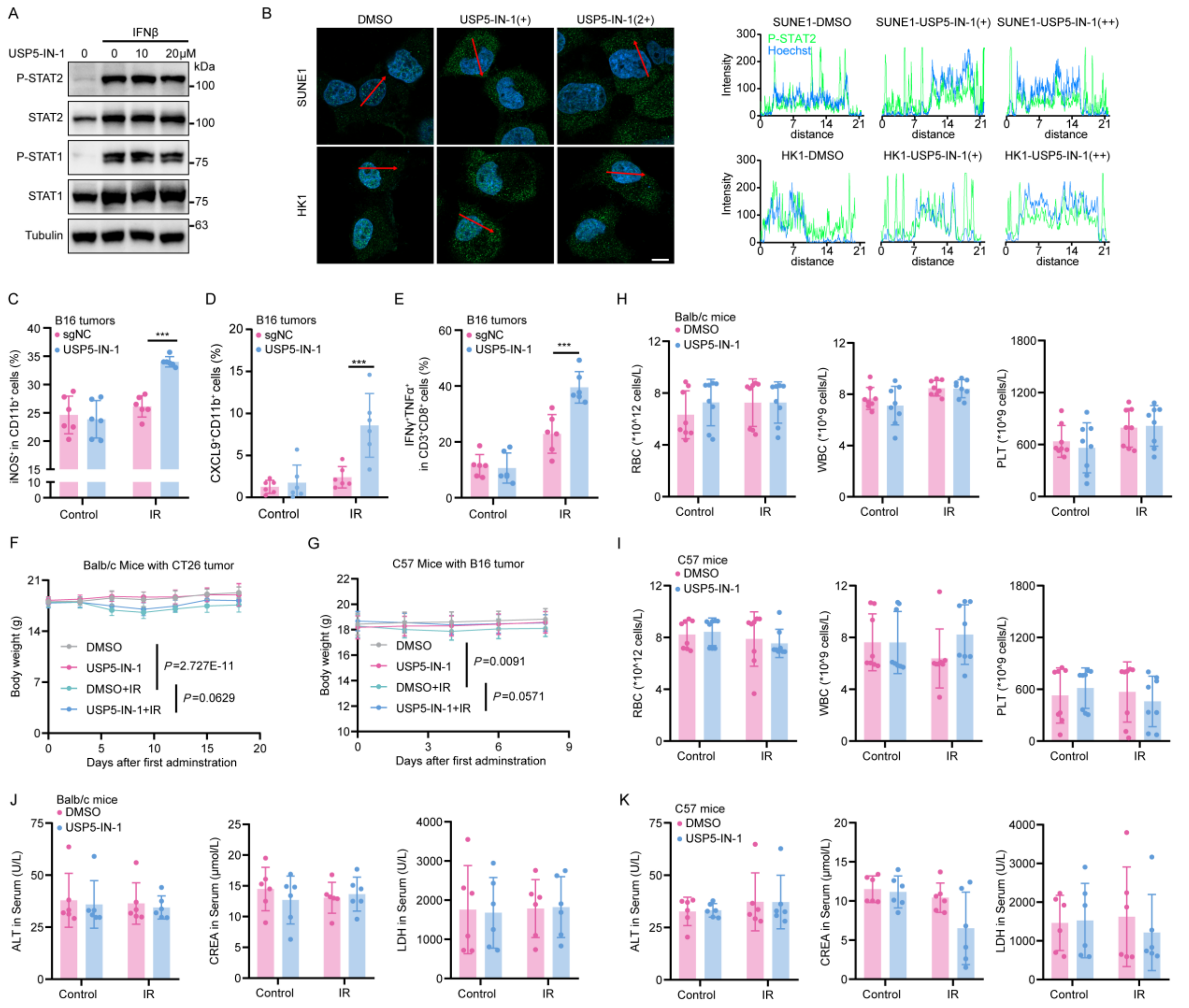

Supplemental Figure 11 (Extended data related to Main Figure 7 and Figure 8)

(**A**) The total and phosphorylated STAT2/STAT1 protein levels in SUNE1 cells treated with IFNβ together with USP5-IN-1. (**B**) Representative confocal microscopy images showing the localization of P-STAT2 in SUNE1 and HK1 cells after IFNβ and USP5-IN-1 treatment. Line scan analyses of fluorescence intensity along the indicated lines are shown. Scale bar, 10 μm. (**C**) Flow cytometric analyses showing the elevated iNOS expression of CD11b^{+} macrophages in B16 tumors with USP5-IN-1 and IR treatment (n=6 in each group). (**D**) Quantitative results of CXCL9^{+}CD11b^{+} macrophages in control and USP5-IN-1-treated B16 tumors (n=6 per group). (**E**) Flow cytometric analyses showing the increased IFNγ^{+}TNFα^{+} CD3^{+}CD8^{+} T cells in B16 tumors with USP5-IN-1 and IR treatment (n=6 in each group). (**F** and **G**) The body weights of tumor-bearing BALB/c or C57 mice with indicated treatments (n=8 in each group). (**H** and **I**) The blood routine tests of red cells, white cells, and platelets in tumor-bearing

BALB/c or C57 mice with indicated treatments (n=8 in each group). (**J** and **K**) The blood biochemical tests of ALT, CREA, and LDH levels in tumor-bearing BALB/c or C57 mice with indicated treatments (n=6 in each group). The results are representative of three independent experiments (**A** and **B**). The data are presented as the mean ± SD, and the comparisons were performed using two-way ANOVA with Bonferroni's test for multiple comparisons (**C**-**G**). ***$P < 0.001$.

Supplemental Table 1. Genes upregulated in *CXCL9*^{high} macrophages in NPC single cell RNA sequencing data.

gene	p-value	avg_log2FC	pct.1	pct.2	p-value adjusted
CXCL9	8.77E-32	0.881848	0.448	0.242	2.03E-27
C1QB	2.27E-27	0.804268	0.489	0.322	5.25E-23
C1QA	1.21E-24	0.76746	0.494	0.342	2.8E-20
C1QC	1.24E-23	0.736674	0.493	0.351	2.86E-19
ADAMDEC1	5.94E-17	0.718157	0.279	0.159	1.37E-12
CXCL10	3.31E-27	0.702402	0.595	0.411	7.64E-23
CMKLR1	3.3E-26	0.692381	0.399	0.229	7.63E-22
SLAMF7	3.34E-26	0.672762	0.426	0.251	7.72E-22
RASSF4	1.5E-41	0.66438	0.683	0.528	3.46E-37
IDO1	2E-13	0.649743	0.325	0.213	4.62E-09
ENPP2	3.7E-17	0.625918	0.279	0.152	8.55E-13
FGD2	4.46E-17	0.615455	0.454	0.317	1.03E-12
CIITA	1.53E-23	0.612002	0.573	0.414	3.53E-19
CD38	2.82E-16	0.604306	0.285	0.162	6.52E-12
C3	1.13E-20	0.600128	0.544	0.407	2.6E-16
SLCO2B1	1.06E-19	0.596008	0.473	0.349	2.44E-15
C2	5.68E-13	0.582678	0.273	0.169	1.31E-08
SAMD9L	1.06E-22	0.573144	0.617	0.478	2.46E-18
ACP2	1.59E-17	0.566877	0.429	0.31	3.66E-13
GSAP	6.25E-17	0.55943	0.472	0.336	1.44E-12
OLFML2B	1.54E-14	0.556871	0.339	0.228	3.55E-10
NR1H3	1.27E-12	0.5543	0.288	0.18	2.94E-08
GBP1	9.84E-32	0.550993	0.753	0.661	2.27E-27
ENTPD1	1.7E-15	0.550814	0.419	0.289	3.94E-11
ITPRIPL2	1.72E-15	0.550305	0.492	0.378	3.96E-11
VASH1	1.45E-26	0.547812	0.71	0.581	3.34E-22
ST3GAL5	3.24E-13	0.54679	0.307	0.196	7.49E-09
SGPL1	1.03E-20	0.546473	0.544	0.436	2.38E-16
TCF4	9.89E-16	0.545222	0.407	0.28	2.28E-11
CD84	3.21E-18	0.544184	0.539	0.414	7.41E-14
NHLRC3	1.55E-14	0.539415	0.317	0.197	3.59E-10
SEMA4A	1.09E-19	0.536524	0.502	0.363	2.51E-15
PTMS	1.29E-16	0.53649	0.594	0.524	2.98E-12
AHR	1.09E-18	0.534713	0.545	0.424	2.51E-14
SAMHD1	1.99E-55	0.534371	0.871	0.745	4.61E-51
SYNGR2	5.02E-30	0.533631	0.748	0.659	1.16E-25
PALD1	2.36E-14	0.532182	0.358	0.245	5.45E-10
SERPINF1	1.65E-11	0.526677	0.418	0.331	3.81E-07
GBP4	2.88E-20	0.525945	0.61	0.467	6.65E-16
SFT2D2	1.35E-12	0.5236	0.448	0.344	3.12E-08
SLC8A1	2.51E-28	0.522616	0.75	0.653	5.8E-24
TTYH3	3.02E-17	0.522552	0.545	0.449	6.98E-13
GSN	1.34E-39	0.513827	0.794	0.703	3.09E-35
P2RY13	2.7E-11	0.513469	0.353	0.252	6.24E-07

MMP14	1.03E-17	0.51298	0.561	0.444	2.38E-13
ADAP2	8.32E-19	0.512368	0.564	0.462	1.92E-14
PHACTR2	1.1E-14	0.512242	0.396	0.276	2.55E-10
ITPR2	7.65E-17	0.510724	0.582	0.477	1.77E-12
VOPP1	3.06E-18	0.510389	0.554	0.437	7.08E-14
RGL1	3.03E-12	0.508037	0.312	0.208	6.99E-08
CD4	1.12E-26	0.502178	0.699	0.607	2.58E-22
PTAFR	1.05E-30	0.502065	0.722	0.601	2.42E-26
TCN2	5.47E-12	0.499353	0.395	0.301	1.26E-07
PLD3	1.82E-12	0.496559	0.565	0.515	4.2E-08
HAPLN3	4.55E-13	0.495161	0.356	0.241	1.05E-08
MARCKS	1.24E-43	0.494545	0.83	0.731	2.87E-39
TBC1D2B	4.09E-12	0.490352	0.339	0.226	9.44E-08
PARP14	6.16E-22	0.48591	0.771	0.671	1.42E-17
STAT1	7.28E-36	0.485833	0.821	0.754	1.68E-31
RASSF2	1.64E-14	0.485558	0.41	0.281	3.79E-10
APOL3	1.03E-11	0.479658	0.355	0.241	2.38E-07
CTSC	4.62E-33	0.477299	0.816	0.761	1.07E-28
CSF2RB	2.98E-11	0.475748	0.366	0.26	6.87E-07
STAT2	6.75E-14	0.473174	0.653	0.567	1.56E-09
LRRK2	1.55E-07	0.470455	0.268	0.193	0.003583
PLXNC1	2.37E-16	0.470066	0.563	0.461	5.46E-12
UTRN	1.34E-11	0.470006	0.538	0.455	3.09E-07
ZNF518A	6.58E-09	0.46651	0.264	0.18	0.000152
POLR2J3.1	1.8E-09	0.462291	0.476	0.389	4.16E-05
RGS12	2.54E-09	0.462018	0.398	0.313	5.86E-05
HLA-DOA	3.75E-11	0.460326	0.317	0.216	8.65E-07
NAIP	7.01E-15	0.455905	0.563	0.455	1.62E-10
AXL	4.05E-12	0.45467	0.474	0.382	9.35E-08
TRIM56	5.23E-07	0.453459	0.337	0.263	0.012081
TAPBP	1.43E-20	0.449522	0.745	0.682	3.3E-16
TFEC	7.97E-18	0.447832	0.625	0.534	1.84E-13
LGALS3BP	4.66E-12	0.444447	0.468	0.37	1.08E-07
TNFSF10	2.16E-15	0.444353	0.681	0.579	4.99E-11
SPRED1	8.67E-09	0.443615	0.301	0.222	0.0002
RUFY3	9.43E-08	0.443313	0.254	0.177	0.002179
TMEM176B	2.2E-33	0.442744	0.767	0.733	5.09E-29
IFIH1	1.2E-09	0.440881	0.468	0.387	2.76E-05
ZBED1	1.15E-11	0.439075	0.336	0.224	2.65E-07
MILR1	7.58E-08	0.438009	0.261	0.185	0.001749
OTULINL	2.05E-11	0.437797	0.476	0.389	4.74E-07
OAZ2	4.95E-15	0.437334	0.56	0.467	1.14E-10
HLA-DRB5	1.32E-24	0.435916	0.737	0.611	3.04E-20
SNX29	5.23E-09	0.433654	0.364	0.274	0.000121
SIGLEC1	1.13E-08	0.432909	0.47	0.408	0.00026
DAPK1	5.26E-10	0.429553	0.329	0.233	1.21E-05
RNF213	8.06E-28	0.427124	0.877	0.838	1.86E-23
AP1B1	2.24E-11	0.426949	0.527	0.452	5.16E-07

APOE	2.06E-06	0.425258	0.433	0.373	0.047689
TLR7	3.52E-10	0.424668	0.293	0.197	8.14E-06
TNFAIP2	8.42E-15	0.424611	0.693	0.614	1.94E-10
SLC2A6	5.84E-10	0.424437	0.265	0.168	1.35E-05
CTNND1	6.64E-11	0.423174	0.549	0.466	1.53E-06
CBX6	2.29E-08	0.422813	0.385	0.297	0.000528
NUB1	2.54E-11	0.421712	0.55	0.472	5.86E-07
SPPL2A	3.82E-16	0.420382	0.637	0.558	8.81E-12
VPS13C	1.46E-09	0.420263	0.546	0.468	3.36E-05
PLXND1	2.54E-12	0.41954	0.524	0.443	5.87E-08
TMEM176A	1.62E-18	0.415877	0.666	0.651	3.75E-14
TSPAN4	1.24E-07	0.412605	0.335	0.266	0.002875
FKBP5	2.25E-10	0.41211	0.393	0.29	5.2E-06
DMXL2	9.5E-11	0.41201	0.534	0.457	2.19E-06
ARHGAP25	7.73E-08	0.410426	0.372	0.291	0.001786
ZNRF2	1.03E-09	0.410162	0.356	0.26	2.37E-05
FGL2	1.71E-47	0.410106	0.883	0.808	3.96E-43
IGF2R	7.81E-08	0.409132	0.457	0.397	0.001803
LPCAT2	1.16E-09	0.408004	0.452	0.371	2.68E-05
IL18	1.12E-06	0.407332	0.434	0.38	0.025863
GNB4	3.48E-13	0.40656	0.587	0.515	8.04E-09
MBNL1	1.21E-13	0.404407	0.704	0.644	2.78E-09
CD163	4.07E-07	0.404294	0.466	0.421	0.009396
SETX	9.4E-09	0.401948	0.494	0.408	0.000217
RHOQ	4.34E-11	0.400408	0.505	0.411	0.000001
PLEKHO1	1.68E-23	0.399872	0.786	0.735	3.87E-19
NUDT3	7.57E-07	0.39856	0.446	0.38	0.017489
EHBP1L1	4.08E-13	0.397727	0.697	0.606	9.43E-09
TXNIP	1.02E-20	0.396729	0.775	0.715	2.36E-16
NFE2L3	1.24E-08	0.39573	0.255	0.171	0.000286
PARP12	1.19E-06	0.395469	0.391	0.328	0.027407
GIMAP8	3.21E-10	0.393089	0.315	0.217	7.4E-06
TAP1	1.68E-11	0.393003	0.656	0.595	3.87E-07
ETV6	7.23E-11	0.39241	0.632	0.562	1.67E-06
TAP2	4.72E-09	0.391792	0.456	0.371	0.000109
IFI44L	1.7E-08	0.391299	0.62	0.601	0.000393
TACC1	4.1E-10	0.390796	0.56	0.493	9.47E-06
FMNL3	4E-08	0.390649	0.35	0.26	0.000924
DPP9	2.73E-09	0.390005	0.346	0.253	6.31E-05
SIGLEC10	2.68E-10	0.388532	0.561	0.489	6.18E-06
C1orf54	2.37E-07	0.388528	0.291	0.216	0.005482
IKBKE	1E-08	0.387156	0.315	0.226	0.000231
NADK	7.55E-10	0.38693	0.499	0.415	1.74E-05
HLA-DQA1	5.46E-24	0.386604	0.81	0.77	1.26E-19
STAT5A	1.05E-08	0.385084	0.383	0.293	0.000242
LARP4B	4.14E-07	0.383621	0.328	0.252	0.009564
ZEB2	4.05E-21	0.382447	0.83	0.788	9.36E-17
N4BP2L2	7.72E-09	0.382005	0.635	0.593	0.000178

MACF1	4.02E-07	0.38192	0.573	0.531	0.009279
CREBL2	1.77E-08	0.381611	0.42	0.347	0.00041
LILRB2	4.07E-20	0.381415	0.778	0.72	9.41E-16
GAB3	1.02E-08	0.381327	0.257	0.17	0.000235
BMP2K	2.42E-09	0.380316	0.565	0.508	5.59E-05
LGALS2	1.17E-11	0.379058	0.602	0.511	2.7E-07
C6orf62	4.18E-11	0.377397	0.672	0.631	9.65E-07
ADA2	1.34E-14	0.375463	0.667	0.607	3.09E-10
SECTM1	1.8E-10	0.373962	0.555	0.481	4.16E-06
PARP9	2.21E-07	0.370139	0.559	0.513	0.005099
TNS3	1.75E-07	0.369818	0.358	0.28	0.004034
CHD9	3.35E-09	0.368933	0.612	0.546	7.73E-05
TRIM14	2.01E-06	0.368818	0.327	0.26	0.046436
G3BP1	5.36E-07	0.368562	0.418	0.343	0.012376
JAK3	7.69E-08	0.36788	0.311	0.222	0.001776
LILRB1	1.1E-12	0.366521	0.713	0.662	2.55E-08
FPR3	7.77E-25	0.366319	0.811	0.741	1.8E-20
HLA-DMB	9.82E-23	0.364994	0.758	0.707	2.27E-18
GOLGA4	2.05E-06	0.364205	0.464	0.391	0.04732
KLF13	1.71E-07	0.361878	0.429	0.34	0.00395
RRBP1	1.93E-11	0.360943	0.67	0.603	4.47E-07
GAA	1.64E-12	0.360855	0.594	0.539	3.78E-08
GM2A	1.02E-07	0.360609	0.463	0.4	0.002357
LPAR6	3.36E-07	0.360472	0.391	0.314	0.007766
SPINT2	4.56E-09	0.360053	0.636	0.602	0.000105
FYB1	9.4E-31	0.359579	0.86	0.816	2.17E-26
GNPTAB	3.78E-08	0.357872	0.387	0.297	0.000872
RCC2	3.91E-08	0.357485	0.459	0.374	0.000902
AKR1A1	5.98E-13	0.357074	0.707	0.686	1.38E-08
TLR4	1.62E-06	0.356805	0.367	0.297	0.037449
APOL2	5.72E-07	0.355618	0.296	0.215	0.013205
NARS	8.06E-07	0.353906	0.496	0.438	0.018626
DNAJC13	3.81E-07	0.351224	0.288	0.208	0.008796
SLC43A2	7.31E-19	0.351131	0.838	0.768	1.69E-14
SLC15A3	6.49E-09	0.350311	0.558	0.511	0.00015
P2RY6	1.66E-06	0.348977	0.35	0.284	0.038373
SPATS2L	9.34E-07	0.347795	0.503	0.447	0.021575
SERPING1	1.54E-09	0.347487	0.574	0.528	3.56E-05
CYFIP1	9.67E-07	0.345448	0.444	0.39	0.022331
SMURF2	2.99E-07	0.344877	0.364	0.281	0.006911
FKBP15	9.22E-08	0.344247	0.503	0.443	0.00213
MNDA	3.42E-18	0.344196	0.774	0.713	7.91E-14
MS4A6A	2.23E-30	0.344082	0.862	0.806	5.16E-26
ADAR	6.61E-09	0.343177	0.683	0.642	0.000153
PNPLA6	2.13E-06	0.343073	0.41	0.337	0.049084
CYBB	6.68E-37	0.342533	0.859	0.841	1.54E-32
VCP	1.54E-06	0.341375	0.566	0.518	0.035452
MPEG1	1.15E-10	0.341197	0.617	0.573	2.67E-06

KCTD12	1.27E-13	0.338647	0.691	0.632	2.92E-09
KIAA0930	7.11E-07	0.337835	0.488	0.425	0.016413
CTSZ	1.42E-42	0.334283	0.874	0.86	3.29E-38
NCKAP1L	5.41E-10	0.333879	0.614	0.559	1.25E-05
ATOX1	1.48E-15	0.329749	0.779	0.802	3.41E-11
PLEKHO2	2.18E-07	0.327888	0.531	0.473	0.005043
LILRB3	1E-08	0.324945	0.656	0.609	0.000232
ABHD12	1.62E-07	0.323155	0.53	0.481	0.003741
SCPEP1	2.02E-07	0.322759	0.552	0.51	0.004672

Supplemental Table 2. **Correlations between USP5 expression and the clinical characteristics of patients with locoregionally advanced NPC.**

Characteristics	Low expression group N = 159 (100%)	High expression group N = 71 (100%)	*P* value*
Age			0.2275
≤45	92 (57.9)	35 (49.3)	
>45	67 (42.1)	36 (50.7)	
Gender			0.0949
Male	120 (75.5)	46 (64.8)	
Female	39 (24.5)	25(35.2)	
TNM stage			0.9493
III	97 (61.0)	43 (60.6)	
IV	62 (39.0)	28 (39.4)	
Death			2.0355E-9
Yes	15 (9.4)	31 (43.7)	
No	144 (90.6)	40 (56.3)	
Disease			1.2435E-17
Yes	22 (13.8)	50 (70.4)	
No	137 (86.2)	21 (29.6)	
Distant metastasis			7.4671E-10
Yes	14 (8.8)	31 (43.7)	
No	145 (91.2)	40 (56.3)	

Supplemental Table. 3 Different proteins in anti-STAT2 immunoprecipitate from sgUSP5 cells VS sgNC cells

Gene names	logFC	Coverage [%]	Gene names	logFC	Coverage [%]
TEX264	12.37842229	11	ARPC2	-0.586248103	18
JOSD1	11.20880071	4	DMAP1	-0.598479193	7
COL17A1	11.0322483	3	DDX17	-0.600535616	32
SYAP1	11.00164867	10	SRSF7	-0.601162568	9
PLEKHB2	10.80181041	3	GC	-0.605494492	2
STON2	10.64159727	1	TRIM28	-0.61286188	6
FDFT1	10.59382335	10	DYNC1LI1	-0.613741318	7
GALNT2	10.55824465	8	RPL29	-0.613860024	9
CD2AP	10.3782448	9	KHSRP	-0.616439969	9
AKAP11	10.35256544	4	LSM12	-0.620414099	14
VPS45	10.34723622	9	ARPC1B	-0.620486679	18
AIFM2	10.32237299	3	PHACTR4	-0.621840531	1
STX12	10.16317574	10	CTSB	-0.622277573	9
LMF2	10.12330853	1	SGTA	-0.626482068	12
RAB21	10.10566205	11	MCM7	-0.626510142	12
SPG21	10.08862716	12	ADD3	-0.627746339	2
SLC7A1	10.02274264	2	AURKAIP1	-0.631494988	5
NDRG1	9.973363733	4	CSNK1A1	-0.636826015	9
NEDD4	9.807621922	5	SH3BP4	-0.637505053	2
IKBIP	9.760639041	3	LRPPRC	-0.640144394	10
SORD	9.705210265	5	DSP	-0.645123748	11
RABGAP1	9.628630117	3	RPL31	-0.648157543	14
ANKRD13A	9.559528184	4	ATXN2	-0.648242184	4
RNF114	9.553031259	10	LMNA	-0.650888875	48
PTPN23	9.403046847	3	CNOT2	-0.652438759	3
RAB1A	9.373589968	24	CPSF6	-0.653036813	5
AKR1B15	9.32758482	5	NDC1	-0.653907133	3
HPS5	9.293433441	5	PRPF19	-0.654740984	19
SLC27A2	9.287633932	5	ELAC2	-0.655397128	1
SCYL1	9.272621406	3	PES1	-0.659450001	3
TRAF3	9.266043722	5	MYH9	-0.65950212	30
SHMT1	9.264796933	8	SNRPB	-0.659602766	13
RNF167	9.257012928	3	DDX5	-0.665191672	31
SLC49A4	9.211953442	3	GALK1	-0.672493421	8
MTMR3	9.20575917	4	ARPC5L	-0.67858735	16
VAC14	9.148309125	2	TOP1	-0.681992506	8
CHD2	9.130678176	1	HDAC1	-0.685561568	4
NEK9	9.118657115	3	LTF	-0.687609118	3
DHX29	9.081594581	1	SEPTIN7	-0.695078522	6
RAB27B	9.06823002	9	IPO9	-0.706076055	2
VPS28	9.033757028	13	H1-3	-0.709676068	15
KIRREL1	9.004064492	3	DCAF13	-0.71097005	2
ATG4C	8.923048225	4	MT-ATP6	-0.71124718	4
LY6K	8.920464959	12	RPS9	-0.716388171	41
PDCD6	8.907370726	5	TUBB2A	-0.719771346	53
SLC12A4	8.894519376	3	PELO	-0.724167456	11
RAB12	8.885648074	20	H1-10	-0.724576509	16
CD320	8.877834971	3	DBN1	-0.731382728	27
RAB9A	8.824474986	13	TJP2	-0.734229758	4

TRIM44	8.76147703	4	APOE	-0.739183584	9
EIF3J	8.724012766	7	NOL7	-0.747625615	3
UCHL3	8.670022651	4	AFG2A	-0.747854673	3
ZDHHC20	8.624573966	8	KIF5B	-0.751804881	10
GBF1	8.605887249	1	MCM2	-0.752628389	1
VPS37A	8.595579536	3	KRT15	-0.7586191	13
B4GALT5	8.587107171	7	ALDH2	-0.768565494	5
SLC39A8	8.571514105	2	H2AC20	-0.771856201	18
RAB27A	8.566560089	9	MTDH	-0.773874588	7
OPTN	8.557337843	23	GRWD1	-0.780004221	9
ACVR1	8.546465851	3	FABP4	-0.781303824	15
SH3GLB2	8.514135884	5	MT-CO2	-0.782628969	4
TRAF1	8.487065644	3	RPL38	-0.783528716	31
SLCO4A1	8.482127196	2	EED	-0.803739179	2
TRIP6	8.467959232	11	CPSF3	-0.804635606	4
USP8	8.446321885	4	ATAD3A	-0.80528558	12
NOCT	8.433984807	3	MYH2	-0.810454748	34
TRIM16	8.415171942	2	HSDL2	-0.81327567	5
AGL	8.373786103	1	CAPZB	-0.818217715	35
ISOC1	8.373601042	9	FAM83B	-0.831202848	16
PPP6R1	8.34293621	2	ASH1L	-0.83372146	0
STXBP2	8.33768649	2	CAPZA2	-0.83386535	37
MYL10	8.296205566	13	TXNDC12	-0.836454443	5
BGN	8.293612345	2	PSMA6	-0.842179902	18
VTI1B	8.282289	4	RPL30	-0.843321678	28
UGGT1	8.278634521	1	TWF1	-0.848477089	6
ARHGDIA	8.271464388	5	HGH1	-0.84875436	3
PTPN9	8.251071141	5	NUFIP2	-0.850555137	36
ADIPOQ	8.155036141	6	CIB3	-0.856973145	7
YIPF5	8.154110719	5	WDR36	-0.860884537	1
MRPS27	8.153436062	4	LARP4	-0.86149392	7
FKBP2	8.105925273	8	RFC4	-0.862421709	15
TRAF6	8.091852429	2	GNAS	-0.862891021	15
WDFY1	8.081663928	6	WDR82	-0.865801448	5
GABARAPL2	8.063631667	23	CALM3	-0.866629295	16
DESI1	8.056787491	5	SPCS3	-0.86705276	5
CHMP7	8.034417682	2	CAVIN1	-0.868114077	19
ADPGK	8.019403064	5	F2	-0.86878153	4
CSNK1D	8.008255083	8	AIFM1	-0.86979821	6
YIPF6	7.999592567	5	XRCC6	-0.875347524	21
ILK	7.977050827	3	GEMIN4	-0.875872568	1
OSBP	7.944827566	3	MARK1	-0.877104402	4
XPR1	7.932620412	1	ISG20	-0.878456404	4
TM9SF1	7.928997727	4	TUBB	-0.879008957	63
UFD1	7.928968202	5	SRPK1	-0.889759142	9
RCN1	7.90875098	2	NCBP1	-0.896197078	3
SLC4A7	7.908544377	1	KPRP	-0.897825116	4
PLG	7.908435488	1	DYNC1LI2	-0.903008332	9
POGLUT3	7.876712405	2	SART1	-0.904461561	3
ITGA2	7.865145098	1	GNA13	-0.917986389	11
PPP6R2	7.861284638	2	RBM39	-0.918979946	10
VPS37C	7.851049171	3	MARK2	-0.921065913	6

B4GALT1	7.83171258	5	LYPD3	-0.933415697	15
CCDC25	7.817601243	4	DAP3	-0.935069027	9
IL6ST	7.805154582	1	KIF18B	-0.937531355	3
ASPN	7.799155706	3	IMMT	-0.944727577	18
GLOD4	7.779822215	7	GSDMA	-0.945725065	4
BET1L	7.779775774	12	NDUFB10	-0.948425892	15
GPX8	7.755958846	3	PSMB5	-0.954149492	14
NDRG3	7.748770276	3	NOB1	-0.957946653	5
ABCC3	7.741897495	1	DHX36	-0.958187534	7
HLA-E	7.734321114	11	U2AF2	-0.959783919	17
OXA1L	7.703865353	3	ANXA3	-0.960658623	6
CHMP3	7.6988563	4	RBM15	-0.967804661	3
CD63	7.689105818	4	LAMC2	-0.968168766	4
PSMF1	7.684446967	4	CTNNB1	-0.972458773	6
PEX16	7.67477701	3	PSMA2	-0.97530973	11
CIAO2A	7.657466634	21	NUP153	-0.977578059	6
CSK	7.653870879	2	SUPT16H	-0.982344329	4
S100A8	7.612429026	12	DDX19B	-0.98467217	9
TMED9	7.600946208	5	GLB1	-0.984804084	2
PSMB3	7.575620001	12	PSCA	-1.004128375	9
SLC12A9	7.569085597	1	RPL32	-1.008220814	27
CHMP5	7.561609401	7	CCAR2	-1.011194208	9
VAMP7	7.551597692	12	UPF1	-1.011343961	20
KPNA3	7.548603412	7	ATP5PB	-1.011715227	7
ZFPL1	7.528681486	3	TALDO1	-1.01484254	9
N4BP1	7.52807876	2	RPL17	-1.022783784	33
MYL4	7.526302711	12	GNL3	-1.032255759	4
TM9SF4	7.510888595	3	EIF2B4	-1.034628546	12
STARD3	7.494685835	3	PPP1R18	-1.03780054	2
VPS11	7.479135204	2	ACTN4	-1.037928714	38
UBQLN1	7.475371749	2	ABCD3	-1.042699415	4
MAPK3	7.460792023	8	SF3B3	-1.046623099	7
VTI1A	7.432681499	4	CHTOP	-1.064738455	3
SHC1	7.411094284	2	RPL24	-1.068267131	25
ASNS	7.409333099	2	LMNB2	-1.068455938	8
RELT	7.409175204	7	DEK	-1.090332259	3
PRKAR1B	7.404008444	13	RPS6	-1.095528326	26
SYNPO2L	7.392014212	1	EIF4E	-1.102315634	8
SERPINF2	7.370581352	2	RBM14	-1.102470171	30
STX3	7.368654307	3	LUZP1	-1.111022312	20
DNAJC2	7.35959846	1	H2BC18	-1.130918276	35
GAPVD1	7.357797647	1	RHBDF2	-1.131725169	4
NCBP2AS2	7.355604289	22	RPS23	-1.132376571	16
NOTCH2	7.35220536	1	RBMS2	-1.134274929	7
SELENBP1	7.32650924	2	KLF16	-1.141324662	14
LPP	7.313267352	2	ALYREF	-1.144670089	19
GABARAP	7.302695677	15	SSBP1	-1.145274123	16
MAP1LC3B	7.288120042	22	HERC5	-1.149557669	7
PIGT	7.265600972	2	TNNI2	-1.15314025	14
DYNC1H1	7.260651694	0	LONP1	-1.158555769	2
TRIR	7.257151928	7	SSRP1	-1.160759671	9
DNAJB2	7.239731584	4	A2M	-1.164904617	1

FAM3C	7.190764992	9	LRRFIP2	-1.167761411	6
NDUFS7	7.16779998	7	PTP4A2	-1.169601097	14
CEP131	7.153355065	1	LTV1	-1.170163334	15
SDF2L1	7.128505294	6	CAT	-1.175005445	18
COPZ1	7.06200823	6	STAU1	-1.179481999	20
ELOVL5	7.030872313	3	ACTN1	-1.180702248	24
MTMR4	6.99783495	6	KRT1	-1.181709622	61
SQOR	6.957553382	3	FUBP3	-1.187344019	23
STK24	6.949017745	8	CSTA	-1.189373551	12
VPS26B	6.939553878	4	MYO1E	-1.189594989	32
KEAP1	6.908616245	11	LYAR	-1.190872772	3
AKR1A1	6.899884715	8	CBX8	-1.191440691	2
ZNF207	6.896974867	3	RBM47	-1.195250651	7
SNX27	6.896198244	2	TXN	-1.20115329	12
ARIH1	6.854352812	3	GPBP1L1	-1.21385836	2
BET1	6.842475554	9	KRT10	-1.22240348	46
NDUFV2	6.83299825	5	DHX15	-1.223938304	14
CHM	6.827552234	1	RPL22	-1.226770378	19
PLXNA2	6.823409098	3	SPATS2L	-1.228815207	12
DUSP3	6.810795327	7	SLC25A10	-1.229066545	7
WDR7	6.799231398	1	H2BC21	-1.232340397	36
YTHDC2	6.78087982	2	HNRNPA3	-1.233332524	34
ARFRP1	6.776760886	6	RAVER1	-1.234091201	14
RAB22A	6.769576004	19	FLII	-1.244789378	3
CTDSP1	6.759775562	3	ESD	-1.251243748	12
ATG3	6.727300991	4	THAP11	-1.252494462	3
TMEM63B	6.691706465	1	KRT2	-1.254097484	65
MARCHF5	6.659125938	8	RPS24	-1.258800867	9
CHMP1B	6.656727134	9	KRT14	-1.270302839	53
ATP11C	6.635018954	1	PKP1	-1.274207202	2
ELOVL1	6.621777909	5	HNRNPA1	-1.275329099	35
STRIP2	6.599557457	1	UNC45A	-1.276739876	6
PPP4R1	6.596887446	1	SET	-1.286034162	17
COG6	6.566916587	2	HAL	-1.290806742	6
UNC5B	6.566228442	3	KRT7	-1.293279629	53
COX5A	6.560355473	5	RBM8A	-1.295753796	16
GPRC5C	6.54433528	3	DNAJA3	-1.296054168	5
NEDD4L	6.533338573	3	LGALS3BP	-1.296631277	29
NCEH1	6.519581393	2	KRT5	-1.303124746	49
RABEPK	6.507493187	3	POP1	-1.303439606	10
OCIAD2	6.50118723	6	SPTBN1	-1.306658127	6
EIF4H	6.465589319	4	SVIL	-1.310150863	2
FTH1	6.464935059	49	PDLIM4	-1.311686825	5
RNF141	6.446123963	6	DIMT1	-1.33493451	10
PEDS1	6.413635849	4	FLG	-1.343432634	0
P3H4	6.39068246	3	STAT3	-1.347624946	8
RHOD	6.372074904	4	OASL	-1.353497932	32
UBASH3B	6.372003935	2	KPNA6	-1.354973175	7
RAB5A	6.306952922	22	DDX41	-1.355810541	2
NCOA4	6.293399848	26	PUM2	-1.361308758	1
MPZL2	6.278828792	10	RFC1	-1.362152738	2
RB1CC1	6.276805658	4	EGFL7	-1.367174503	12

MGAT2	6.27308804	4	PAPSS2	-1.369991405	10
MFF	6.255728818	3	CREBRF	-1.379704108	1
IL13RA1	6.253313261	3	BST2	-1.382642454	18
SRC	6.245921542	7	KRT18	-1.394103325	46
MCL1	6.180013263	6	HNRNPM	-1.396408079	43
ENOPH1	6.168468269	5	LASP1	-1.397153577	31
PARP4	6.158584378	2	MATR3	-1.403875646	25
TAB1	6.121431467	2	HNRNPUL1	-1.407570623	15
RAB30	6.120191356	10	MKRN2	-1.411603501	3
ATG101	6.115727167	13	CGAS	-1.411934329	4
CASP7	6.110637146	3	KRT85	-1.412873272	6
EDF1	6.105421307	10	DDX21	-1.424089818	23
CLCN6	6.096698728	1	TRIM21	-1.425727857	29
S100P	6.074201139	11	MRE11	-1.427284185	1
PEPD	6.048701876	4	NCCRP1	-1.429527913	18
FTL	5.903367925	50	RPS28	-1.430018152	30
UBL4A	5.810354529	5	GNB1	-1.432980199	25
LRATD2	5.799015529	11	LLGL1	-1.437382173	5
BMPR2	5.763800673	3	DHX30	-1.44016751	18
NBR1	5.748845303	13	ADAR	-1.440701272	13
TAX1BP1	5.705429537	36	ILF3	-1.460353219	23
KPNA4	5.68616269	7	FGFBP1	-1.460725943	6
SQSTM1	5.67620633	50	AGO2	-1.477423494	8
AP3D1	5.624699934	1	SLAIN2	-1.47876737	9
VN1R5	5.566327497	2	XRCC5	-1.498865147	16
TNFAIP3	5.528147983	18	SNRPA1	-1.509695534	10
ATG9A	5.446639077	12	KANK2	-1.512040115	4
TBK1	5.401194203	23	SERPINB5	-1.524332931	17
ALDH3B1	5.391806187	5	GNAI3	-1.524354139	28
ATG13	5.370100252	12	APOH	-1.531573059	3
HMOX2	5.356839599	6	DAPK3	-1.545875204	7
SIL1	5.291596272	4	DDX24	-1.551332643	7
PCSK9	5.249910748	2	KRT17	-1.5660873	41
CLCN7	5.194272702	3	SPECC1	-1.569906732	18
PLXNB2	5.17091062	2	PARP1	-1.570112774	12
TNIP1	5.161158797	31	CLK3	-1.581769744	3
ANKFY1	5.1254296	11	PABPC4	-1.594285345	34
ANKRD33B	5.073459885	2	RPL23A	-1.59528668	34
STING1	5.039563598	11	C6orf132	-1.609269719	5
TBC1D15	5.00425278	18	COPG2	-1.6096206	3
LRPAP1	5.000979098	14	RPL36AL	-1.612059686	21
ITGB4	4.996017299	10	HNRNPH1	-1.633199035	30
VAPB	4.948139616	21	KRT9	-1.635858614	64
PIBF1	4.94007361	7	YEATS4	-1.637677777	4
CALCOCO1	4.937264158	15	PSMA4	-1.641478589	14
NOTCH1	4.927629792	2	DIS3	-1.642192639	2
COL1A2	4.905227911	1	MYBPC1	-1.645717927	11
VPS39	4.865941069	3	KRT78	-1.646447386	22
REEP4	4.856424262	13	PTBP3	-1.651891517	3
LAMTOR1	4.836531649	15	HNRNPL	-1.660321267	21
VAMP8	4.830963727	22	RALY	-1.665992318	12
MYOF	4.790067683	6	CNNM3	-1.666709841	2

AZI2	4.78085051	19	LMNB1	-1.680068557	14
IFNGR1	4.747904643	10	SEMA3B	-1.693947428	5
ESYT1	4.72019601	4	WDR33	-1.697339835	5
LSG1	4.662732047	6	LEMD2	-1.707642834	4
RDH11	4.641224748	14	EFHD2	-1.710038766	23
RAB43	4.595707853	16	PSPC1	-1.717920773	15
NRDC	4.531163439	3	ABCG2	-1.719904781	6
GLT8D1	4.529044664	9	RRP15	-1.732701508	4
SLC12A7	4.449800774	6	YBX1	-1.73530434	44
CYLD	4.447968649	3	RRP1B	-1.744235599	4
ULK1	4.433219868	5	KRT80	-1.744285732	16
APMAP	4.431658127	11	C11orf98	-1.74702385	11
PI4K2A	4.393946817	19	ILF2	-1.748735524	27
VPS33A	4.388169972	3	PCID2	-1.749843246	7
SPATA2	4.384504406	4	IGHG1	-1.750445844	18
CC2D1B	4.381922147	4	POLD1	-1.753073531	2
PMM2	4.360652026	8	GNAI2	-1.758529877	43
IST1	4.332787873	10	OGDH	-1.765112665	19
SEC62	4.325457463	7	KRT81	-1.769399882	5
PRKAR1A	4.262136588	23	EIF3I	-1.772080842	23
MFN1	4.230598294	1	UBTD1	-1.780452306	6
HGS	4.198503833	5	SSB	-1.786033268	8
TPRG1L	4.190818622	13	CPA4	-1.803575085	5
UAP1	4.13876899	4	KRT8	-1.823935612	60
RAB3GAP1	4.133341	5	HNRNPR	-1.82438392	21
DAGLB	4.126993582	6	NCL	-1.838748681	19
VPS18	4.069181891	2	VTN	-1.839492672	3
SDCBP	4.068092477	13	C4A	-1.852719054	3
FADS1	4.051416566	6	UQCRC2	-1.869726177	10
DDR1	4.044973565	3	IMPA2	-1.875812784	8
PPP6C	4.036061092	7	KRT74	-1.877919373	7
KTN1	4.01064144	1	CMSS1	-1.879674515	3
RAB3GAP2	4.000015661	5	DSG1	-1.88882539	12
IL4R	3.994448477	4	SFPQ	-1.889693034	33
KLHL41	3.975768492	3	ARAF	-1.890444643	6
ARHGAP1	3.974543826	7	GAR1	-1.893817446	20
ATP13A3	3.954737447	6	DDX50	-1.906623888	9
YKT6	3.947812587	21	ACTB	-1.919396885	70
TRIM14	3.943619301	21	FLG2	-1.920162701	6
SERPINH1	3.923439707	20	MACROH2A1	-1.927519003	17
SQLE	3.881758494	4	RALA	-1.930784557	13
RAB31	3.87724334	21	YBX3	-1.966317354	27
DERL1	3.869361338	8	H1-5	-1.968297545	15
PLK1	3.867210299	5	CENPV	-1.971672224	10
CDC37	3.815139374	5	FIP1L1	-1.995351663	3
SAR1B	3.801794963	25	NOP56	-1.998056485	18
PTPRJ	3.766828794	3	XRCC1	-2.006980008	2
PTGES	3.712587279	21	MFGE8	-2.029420454	13
STAM2	3.706521683	4	SP1	-2.030346856	9
ARL8B	3.698276639	15	MYO1C	-2.049012939	36
TXNDC5	3.694049579	21	FBL	-2.053394059	29
GFUS	3.685511609	8	CUL1	-2.065104994	3

CALCOCO2	3.677960768	30	RBMS1	-2.07720324	7
JAK1	3.649364787	8	WDR26	-2.086306623	3
HPS6	3.627807131	10	TMPO	-2.098190389	23
ITCH	3.62138224	10	NSF	-2.103324197	32
PPCS	3.611261146	10	PURB	-2.10824803	7
EPRS1	3.5937352	13	DCP1A	-2.114300275	24
CLTA	3.591555475	10	SHFL	-2.123774578	9
AMPD1	3.588767134	2	FMR1	-2.124160023	17
RBM7	3.587508485	26	DSC1	-2.153684208	7
LCLAT1	3.583760483	7	H2AZ2	-2.24250442	31
SARAF	3.578402102	10	DHX9	-2.256887234	21
PARP10	3.572089738	2	CTSA	-2.264116714	3
CKAP4	3.565288371	32	PPP1CC	-2.269729951	29
LSR	3.564929319	11	DDX54	-2.273625486	10
THOP1	3.55532995	3	EFR3A	-2.293284697	5
VAMP3	3.548352369	24	SPTAN1	-2.297263139	14
MBOAT7	3.530281446	8	CASP14	-2.302841026	28
RNF149	3.50691793	13	FTSJ3	-2.305526207	2
TPM2	3.506470273	40	FXR1	-2.324790133	29
VPS4B	3.506373079	21	NONO	-2.341540088	41
IL7R	3.504196439	8	TRIM56	-2.34503586	6
EIF4A2	3.494087789	22	MRM3	-2.36609078	5
CCPG1	3.477565493	15	CRIP2	-2.372214476	46
ATG16L1	3.468251829	3	MRPL28	-2.372863977	5
NUP155	3.467390915	6	SF1	-2.399242196	21
LRP10	3.46606969	9	RPS7	-2.409630772	15
HPS3	3.461429627	4	ELAVL2	-2.412216767	9
ATP6V1C1	3.454894345	4	ZNF143	-2.420318647	6
RAB3A	3.425606989	14	GTF3C4	-2.436400518	2
GANAB	3.405932988	22	TUBB4B	-2.487763468	66
RHOG	3.399816951	32	SF3B2	-2.50845383	7
RHEB	3.396702434	36	MIF	-2.523732958	8
KCNJ12	3.370321691	7	ZFP36	-2.536394509	4
STX17	3.370255795	10	USP10	-2.541394087	11
DNM1L	3.360406388	17	VIM	-2.547809343	56
PDCD6IP	3.337893546	40	CACNA1B	-2.552000901	1
RAB5C	3.336968568	22	MAP7D3	-2.563136901	5
STAMBP	3.336059636	10	RSL1D1	-2.595365586	13
LMAN1	3.335989503	4	NCOA5	-2.603297073	7
HYOU1	3.320972858	7	PDE4DIP	-2.604677454	2
FABP3	3.310366703	24	CEP78	-2.62378449	4
TSG101	3.309399496	16	NOP58	-2.648592746	11
KAT5	3.30285917	2	SAV1	-2.661115235	2
ELOB	3.289358972	14	PLEKHA7	-2.68609241	2
NAPA	3.279417181	39	ARHGAP45	-2.68887829	3
PCM1	3.279018811	1	VWA3B	-2.705027219	1
PRKCI	3.262597627	7	IRF9	-2.735559129	8
NTMT1	3.262425205	10	EDC3	-2.809760815	14
CALR	3.245746541	14	FAM120A	-2.824360751	24
SLC27A4	3.23887094	7	PTBP1	-2.83588394	11
SPTLC1	3.238332971	16	KRT77	-2.836620696	16
RAB10	3.226917908	24	BLMH	-2.839233421	13

ATP6V1A	3.222824743	3	PLEKHA6	-2.853039142	7
ANGPTL4	3.217973904	5	ELAVL1	-2.864294678	27
TNFRSF10A	3.216604094	2	ARG1	-2.875371311	16
PDIA3	3.195806128	23	CPSF1	-2.887916964	4
ABCC2	3.191905356	2	SUGP2	-2.901898226	2
CANX	3.179788044	18	MOV10	-2.906420467	26
SACM1L	3.173991736	10	GPC1	-2.923232982	10
LGALS9	3.173260954	17	DST	-2.932239044	0
ACAT2	3.166504828	10	PSMD13	-2.98791989	14
KPNA1	3.163777292	8	GGCT	-3.027160803	29
EGFR	3.161530774	7	MYH14	-3.055322845	3
PARP14	3.157879106	1	STOM	-3.087707146	8
PPP6R3	3.151888552	4	BECN1	-3.096289893	24
CRTAP	3.141975325	10	NOL6	-3.106708627	6
ZDHHC13	3.129860563	10	PABPC1	-3.107394426	40
MYO15A	3.126382229	0	CMAS	-3.136313631	6
RNF31	3.126185437	4	MYL12B	-3.164166307	30
RAB32	3.119747835	30	PLAUR	-3.164599876	19
PGD	3.112237357	11	TBC1D10B	-3.182901073	11
BZW2	3.111525269	6	PHLDB2	-3.225249157	20
ATP2C1	3.11093861	8	PRKAR2A	-3.233858049	12
HSD17B12	3.091580598	11	FXR2	-3.237874418	28
TMEM214	3.07750066	3	PURA	-3.368193964	13
FYCO1	3.067452298	9	G3BP2	-3.374430645	34
SERPINB1	3.065898589	14	BASP1	-3.405074558	48
SFN	3.06154078	24	ALPI	-3.4209681	11
PLXNA1	3.053697862	3	ARL6IP4	-3.443347902	13
TRIM26	3.052559021	8	U2SURP	-3.464542	2
STX8	3.047940244	3	ZFR	-3.50394662	5
SAFB	3.043636388	12	TOP3B	-3.510224574	5
ATP11A	3.043362887	2	CD109	-3.513238691	5
GSK3B	3.038419708	15	SCRIB	-3.519164629	1
TRIM32	3.037610234	17	CAPZA1	-3.537393955	34
UBE2M	3.029377287	17	LUC7L2	-3.56431754	9
KCNN4	3.028246813	4	G3BP1	-3.580491931	41
SNX3	3.023922532	6	PPP1R10	-3.582044513	8
COL1A1	3.015881861	2	DKC1	-3.633934482	11
PGAM1	3.008196703	35	TMOD3	-3.644311662	36
SERPINE1	3.000749938	5	ARPC3	-3.710192717	16
CHMP4B	2.989146952	16	GTF2IRD1	-3.746045388	21
APOB	2.98817924	1	MSLN	-3.785670764	4
SLC20A2	2.980398777	2	CASC3	-3.810581055	13
CMBL	2.961854115	8	CEP89	-3.817288561	6
EPHB4	2.960985245	4	TDRD3	-3.835065338	8
GOSR1	2.960094224	9	KLF5	-3.869867356	20
ELFN2	2.957210541	4	DDX18	-3.918237194	8
ATP2B1	2.956774389	6	ZC3H7A	-4.077243589	23
CMPK1	2.943555648	16	FAM83H	-4.121296323	17
AASDHPPT	2.927886052	11	ERC1	-4.255323065	14
TOR1B	2.925262236	2	MYO6	-4.258866906	16
ERP44	2.920141652	10	CNBP	-4.260269651	31
BROX	2.91028823	21	STAU2	-4.28317454	10

TPT1	2.901625223	12	TPM4	-4.29926195	36
MRPL24	2.89970859	4	ABL2	-4.354382252	1
ZMPSTE24	2.899089757	3	NT5E	-4.356167766	32
SLC12A2	2.877086168	3	ASCC1	-4.358390719	2
PDIA4	2.874559494	28	RAI14	-4.368149613	31
MTPAP	2.869760841	15	MYO1G	-4.440759495	37
UBXN6	2.853484754	5	LMO7	-4.454127239	27
SAR1A	2.850519551	20	INTS6	-4.576010107	3
TMX1	2.844715247	19	PIK3R4	-4.608087915	30
RAB20	2.838744147	24	LIMA1	-4.649227148	37
AIMP2	2.830206254	25	ABLIM1	-4.662529055	8
PGRMC1	2.826536741	16	GNG12	-4.708133011	35
IFITM3	2.826055983	31	ZNF185	-4.719981653	26
PPP1R13L	2.813166688	14	IGF2BP1	-4.744038745	21
TMEM43	2.811072772	14	MYO1D	-4.747930015	6
NDUFA6	2.809300087	15	FAM120B	-4.845100588	5
CLPTM1	2.80425459	10	SPECC1L	-4.849303465	15
RAB18	2.792003279	15	MYO1B	-4.85889302	39
PRNP	2.785649548	13	MX2	-4.897728228	43
ATL3	2.781375698	6	SPOP	-4.944462167	17
WBP2	2.773151501	11	ZSCAN26	-5.016004714	2
CYP51A1	2.767910893	4	FMNL2	-5.020934315	10
GDI1	2.76285385	8	KRI1	-5.059611456	2
APOL2	2.761141182	8	AIP	-5.130078709	9
TNFRSF21	2.751038985	12	GTF3C5	-5.134802368	5
EHD1	2.749104079	16	SDR9C7	-5.170693394	3
PPIB	2.734723662	41	CD55	-5.183265362	42
NUDCD2	2.722554337	19	NRBF2	-5.267399482	42
TNFRSF10B	2.718357408	6	PIK3C3	-5.269336533	26
AIMP1	2.718339306	25	TENT5A	-5.299335548	3
LSS	2.712439723	5	RSPRY1	-5.390324722	2
TOM1L1	2.71166311	9	ALPP	-5.413283049	22
ZC3HAV1L	2.708536126	4	CCDC9B	-5.447611312	5
CSTB	2.707367263	24	DNPEP	-5.453550377	25
EIF5	2.702645229	7	KLF10	-5.526213868	3
FBN1	2.702486576	1	MISP	-5.570576453	44
IL1RAP	2.700808918	4	NUDT5	-5.582261197	7
SLC25A20	2.696883112	6	RITA1	-5.596915757	11
TIFA	2.694586798	20	PYCR2	-5.641474498	10
NAPG	2.685518988	3	DIAPH3	-5.643224524	1
PCMT1	2.685405405	15	DDX56	-5.665846978	6
PRKCSH	2.682226487	10	CTBP2	-5.665970419	2
PLAA	2.676765494	5	CCDC137	-5.676605525	5
RIPK1	2.676201677	3	ACTR1A	-5.684638468	6
VPS4A	2.67615392	16	SENP1	-5.685461285	3
ITGA5	2.674886716	2	MRPS17	-5.696473062	7
PRRC2A	2.673691822	2	CHAMP1	-5.70013079	2
CDCP1	2.668215344	11	GNL3L	-5.71277785	4
TPD52L2	2.659484466	20	UTP18	-5.743633606	2
DHCR24	2.658793177	7	SPTY2D1	-5.749322135	4
RAB7A	2.658603714	38	ZMYM1	-5.812778335	17
SEC22B	2.646922687	9	NKRF	-5.880599709	3

ATL2	2.640776546	2	GTPBP1	-5.896879852	1
PLPP2	2.632040541	11	ANP32E	-5.901369492	5
SLC39A7	2.630510994	3	AMOTL1	-5.917273919	2
PACSIN3	2.626698973	19	SEMA7A	-5.968955792	2
TMEM59	2.626210994	22	PLRG1	-6.00248628	2
TFRC	2.625975783	30	DAG1	-6.023093395	1
ANXA8L1	2.617959956	3	FRG1	-6.066538633	5
VPS41	2.606622833	2	NES	-6.134866812	0
KPNB1	2.593245462	10	ITGA6	-6.165145814	2
ACVR1B	2.578529083	3	GNAO1	-6.181259255	9
TRAF2	2.577838408	19	DDX39A	-6.192326846	17
OSMR	2.577403354	3	DNAJC10	-6.192589205	2
LANCL1	2.57692712	6	TRIOBP	-6.232787582	0
NDFIP1	2.567200035	34	NKD2	-6.242509448	4
PTPRS	2.565154549	3	TRMT1L	-6.2597052	1
COPS4	2.557294334	5	ADAMTSL4	-6.273380904	1
ST13P4	2.55122074	14	STRBP	-6.311184615	7
TOMM34	2.549214559	13	NMT1	-6.329908752	8
ALDH6A1	2.548784013	3	KRR1	-6.379877586	3
ERO1A	2.546260615	9	SEMA3C	-6.383845792	1
PGRMC2	2.542158811	16	PAK1IP1	-6.401620585	3
STUB1	2.533683627	11	USP5	-6.404651152	38
SEC63	2.531068344	3	MPRIP	-6.408936324	43
NSFL1C	2.530463273	10	DDX31	-6.413978611	1
RAB1B	2.530110669	26	LIMCH1	-6.443132323	1
SLC12A3	2.525567326	6	COASY	-6.447036644	2
RAB8A	2.524267895	41	NUDT16L1	-6.485409288	5
FKBP8	2.523768127	7	CD59	-6.500119893	25
HMGCS1	2.514493163	8	HPRT1	-6.617196395	11
PLPP3	2.513671672	4	ACOT13	-6.685716228	7
UBIAD1	2.512512362	6	MARCKS	-6.689482872	10
TMX3	2.508430929	4	PCYT1A	-6.690794722	3
SPRYD7	2.507930649	5	PPP3CB	-6.698786398	4
TNFRSF1A	2.505494593	4	RPS6KB2	-6.701318248	2
DNAJC3	2.502273826	4	RBM28	-6.706429581	3
GOLPH3	2.501871895	5	RTN4R	-6.708163777	2
TCAF1	2.498550191	2	FBXW11	-6.725180158	2
MYH8	2.495116498	24	GPATCH4	-6.732497777	6
PPIA	2.486207575	30	SNRPD2	-6.807019824	8
DNAJA2	2.483315318	11	PRPF6	-6.840536304	1
RIPK2	2.474763732	6	GJC1	-6.864266681	6
PRKCD	2.473585041	3	UVRAG	-6.924879391	3
PAFAH1B2	2.468111503	4	CCL5	-6.935842849	12
AMPD2	2.459172457	2	RNASE7	-6.941738235	9
PODXL	2.458697811	8	CNN3	-6.958051747	3
SNX1	2.457651847	7	ZNF444	-6.983786173	8
CMIP	2.457481328	1	FMNL1	-7.013955463	3
SCAMP3	2.455742408	8	POTEE	-7.173698453	10
HSP90B1	2.451512589	27	LRCH3	-7.209038874	3
PLOD3	2.442960666	4	ZC3H14	-7.223221847	20
EDEM3	2.436728401	1	IGF2BP2	-7.229817073	16
COL14A1	2.436399668	1	GATA2	-7.300413284	12

KLC1	2.430924345	5	CORO2A	-7.325008982	6
PTPN1	2.430719796	20	TSR1	-7.355347961	4
ATXN10	2.424601824	6	AHCYL1	-7.367736466	4
CHMP6	2.424326874	4	EDIL3	-7.404500955	20
ATG5	2.42280794	8	CAMK2G	-7.411620064	4
PTPA	2.411069104	6	AKAP9	-7.470237651	1
PRDX5	2.40771529	5	HELZ2	-7.505425178	1
RAB34	2.402046507	14	ABI1	-7.518687612	5
AKR1B1	2.402038613	17	SMN1	-7.526491163	3
NDFIP2	2.387836817	10	MYH10	-7.532207601	6
COTL1	2.386733436	18	ACTBL2	-7.565079437	14
UFSP2	2.385514473	2	ERAL1	-7.577513518	7
GPRC5A	2.383765831	9	PLS1	-7.586736093	7
DNAJB1	2.381680264	19	NCBP2	-7.591194369	7
EPHA2	2.373250612	21	PLCD3	-7.62993468	6
MPLKIP	2.37057936	22	CPSF2	-7.680644591	4
TBL2	2.36741379	11	GNAI1	-7.711650145	17
FAM20B	2.364366264	2	SPATS2	-7.849678179	10
CAP1	2.36206148	17	AFAP1	-7.899357463	6
PGLS	2.359299047	6	NEXN	-7.942969136	3
RHOF	2.359057466	14	CDH13	-7.955574859	5
ATP6V0D1	2.356052219	4	CPSF4	-8.032614038	4
PLN	2.353688361	21	RBM17	-8.037382482	5
AMFR	2.353301443	1	NIFK	-8.231099732	12
TCIRG1	2.353206257	5	ATP5MG	-8.253637239	11
P4HB	2.352281051	33	KGD4	-8.323385005	12
STX10	2.350539116	8	GRTP1	-8.359898259	3
RPS27A	2.347112386	58	F5	-8.446610667	3
GPD1	2.345451329	8	CDSN	-8.536794011	4
CERS2	2.345401802	9	YTHDF2	-8.537210656	8
P3H1	2.344377875	5	ITPR3	-8.641417098	2
CCDC61	2.336775012	2	ZCCHC3	-8.786249695	18
SEC23A	2.334717203	4	YY1	-8.824666624	2
KARS1	2.329789114	24	MELTF	-9.483376116	10
FRY	2.328397702	0	LIG3	-9.509895088	6
PDXDC1	2.323153869	7	L1RE1	-9.592634694	17
GSK3A	2.322968529	17	TGM1	-9.609466782	6
STX6	2.322483131	9	UACA	-9.65870016	5
PDIA6	2.313815055	24	KLF3	-9.736350156	11
ATP2A2	2.31253528	26	IGKV1-37	-9.952447104	14
CDK2	2.310304641	10	KLF4	-9.997378331	15
HNRNPDL	2.302716165	16	KRT19	-10.24845261	32
ITM2B	2.299747644	7	ITPRID2	-10.27077504	7
SLC39A1	2.298611592	4	PPP1R9B	-10.45335938	14
EIF5A	2.290833775	37	RASA3	-10.83296664	16
SEPTIN11	2.290458415	2	ULBP2	-10.86265031	17
RARS1	2.288157664	29	SHTN1	-11.05842008	1
PITPNB	2.287644051	14	ZBTB34	-11.14772138	6
TBC1D17	2.284988129	9	IGKV4-1	-13.62615088	12
TARS1	2.284955264	16			
ARFGAP1	2.283174316	8			
GLO1	2.28229008	9			

POR	2.281453804	10			
UBE2K	2.276056706	24			
ALOX12B	2.267529805	3			
VPS26A	2.263587113	8			
HACD3	2.256079632	9			
IDH1	2.252204337	9			
UBE2L5	2.248437868	6			
OAS2	2.240587623	21			
DARS1	2.235446567	46			
TAP2	2.234136966	11			
RAB11B	2.233122122	26			
MYDGF	2.225593895	12			
HSPB1	2.225301329	43			
QARS1	2.21328091	32			
CHUK	2.212800255	2			
RER1	2.211442316	10			
TBCB	2.211253766	14			
IL18	2.208328121	4			
RTN3	2.206718643	1			
TELO2	2.193331823	2			
SRP72	2.184292838	8			
AK1	2.183196534	23			
IPO5	2.181829259	10			
KRT16	2.179555972	52			
RRM1	2.179066638	8			
PIP4P2	2.175798126	18			
TNNT2	2.173743417	2			
SH3GL1	2.171611308	9			
PHLDA2	2.168451594	11			
INF2	2.168403403	2			
ANXA1	2.167685423	38			
CYB5B	2.166921987	27			
SPINT2	2.153467562	17			
SLC30A7	2.152958788	3			
PGM1	2.151791989	25			
EEF1E1	2.14751086	11			
NAMPT	2.142792289	15			
VPS35	2.139388927	14			
ADGRL2	2.127773142	1			
ALG5	2.125549229	6			
ARRDC3	2.124929236	12			
CPT1A	2.122646344	9			
SBDS	2.122276171	7			
THRAP3	2.119657084	6			
FSCN1	2.115423495	26			
ALG1	2.115009088	5			
TAPBP	2.108277032	11			
TAF6L	2.104649927	3			
KRT6B	2.100324261	47			
ATP6V1B2	2.099879032	7			
PFN1	2.096710158	59			
TTC4	2.092390332	6			

PSMD6	2.091589124	14			
ENO3	2.091539814	34			
SNX17	2.087205597	18			
PGM3	2.079885191	3			
PRKACA	2.079503939	15			
MET	2.070345007	4			
RAB6A	2.067511018	15			
PLOD2	2.066400072	2			
ITGB1	2.065576641	4			
MSN	2.065481115	18			
YWHAZ	2.065231283	40			
TTLL12	2.057715842	6			
ENO1	2.053132605	38			
NSDHL	2.053058317	9			
SLC25A4	2.051772519	27			
TMEM33	2.051395652	20			
MVP	2.048451634	32			
TUBGCP2	2.040352223	1			
STX7	2.034978764	9			
FARSA	2.034828135	6			
MROH1	2.026938479	0			
SF3B1	2.026180051	7			
VPS16	2.024063999	4			
RRM2	2.023928955	6			
MRPS12	2.019887953	7			
MAGT1	2.01900688	3			
PARVA	2.017912974	6			
EEF2	2.017322666	32			
YWHAH	2.014257166	29			
TRIM29	2.011135423	23			
AP1B1	2.01022907	7			
FABP5	2.009081676	15			
ALDH3A2	2.007518097	13			
ANKRD28	1.994878385	5			
GPI	1.993081606	18			
FLCN	1.989574349	9			
STX5	1.98339264	3			
VCP	1.982426787	25			
CRK	1.975696094	3			
TMEM11	1.965137397	4			
DDOST	1.964424926	9			
UBE2NL	1.962193043	7			
PLCH1	1.961246467	0			
ATP1A2	1.953761186	7			
AP3B1	1.950545078	6			
TRAPPC3	1.949243733	4			
S100A11	1.944610017	17			
HSPA2	1.942936646	16			
GOLGA7	1.941190394	6			
SGCA	1.939859385	2			
UBE3C	1.938974752	2			
CKMT2	1.93845881	5			

ANXA5	1.936406151	20			
UBA1	1.933287141	11			
EMC1	1.929656861	2			
RPS18	1.926842549	44			
NIBAN2	1.914057443	9			
ADAM10	1.911516709	3			
OTUB1	1.909833431	10			
CASQ2	1.90564221	4			
UNC13D	1.903862646	1			
ATP2A1	1.897247349	22			
SLC38A1	1.892175823	2			
PLEKHH3	1.891596241	3			
NXN	1.88716653	2			
PGK1	1.88278391	49			
COQ9	1.881829175	3			
PLSCR1	1.875925482	6			
PVALB	1.861106141	7			
TNC	1.859483198	1			
GLRX3	1.858826235	13			
RHOA	1.856590478	24			
EHD4	1.853549809	19			
COPA	1.852148714	17			
AXL	1.850832473	6			
FHL1	1.847450153	13			
PAFAH1B1	1.846235671	14			
ABHD12	1.845854243	2			
QPCTL	1.83994495	5			
MYL2	1.838898599	30			
CAPN7	1.83713219	11			
TMCO1	1.836258211	15			
EIF3G	1.834772802	11			
GARS1	1.826359342	10			
MYH6	1.824591775	31			
TUSC2	1.82221263	8			
CTPS1	1.816742805	27			
EPPK1	1.813582549	6			
YIPF4	1.812770944	4			
SGPL1	1.811996365	5			
PRMT3	1.811500788	4			
HSPH1	1.808876809	18			
FN1	1.808559159	1			
NDRG2	1.804735382	3			
ATIC	1.800639215	22			
ACO2	1.799925041	13			
MGST2	1.79624111	10			
CBR1	1.792547592	22			
HTATIP2	1.792200816	29			
DNAJB4	1.791348124	7			
SLC38A2	1.790507738	9			
USP14	1.788777223	4			
ALG8	1.788207664	2			
YWHAG	1.786001791	23			

DNAJA1	1.782563029	24			
ACAA2	1.780220577	7			
ETF1	1.778811052	7			
CAPNS1	1.77776485	12			
SMC4	1.776656076	3			
FHL2	1.776189454	6			
DPM1	1.77554659	16			
CLCN3	1.775442464	2			
ACADS	1.772861157	8			
STOML2	1.771850586	15			
PYGM	1.767572097	19			
AP1G1	1.767322259	6			
RAB2A	1.766776761	24			
COLGALT1	1.755187854	9			
PBK	1.752996574	3			
CFL2	1.748877221	13			
AP3S1	1.74528811	6			
ERBB3	1.743851869	1			
COX6B1	1.743616554	13			
CS	1.737073026	9			
CSRP1	1.72860563	60			
UGDH	1.725817139	9			
ITPRIPL2	1.717285076	7			
OXCT1	1.715840365	2			
PPP2R1A	1.714885262	17			
SPART	1.713149851	17			
YES1	1.712898487	22			
NDUFV1	1.712499888	4			
RPS27L	1.708697577	23			
SLC6A6	1.708411197	5			
YWHAE	1.707639415	49			
FAR1	1.703098299	7			
COPS2	1.701571245	2			
NOMO2	1.697269468	2			
SDF4	1.695246126	2			
DECR1	1.692297916	10			
PPP5C	1.691433028	6			
COPS5	1.690203151	9			
PSMD4	1.689418091	6			
COX5B	1.68921185	9			
ASS1	1.687620862	15			
SLC35E1	1.68552851	2			
CLPTM1L	1.681802017	2			
TMEM97	1.678906157	5			
TOR1AIP2	1.676879046	2			
GYG1	1.67602333	3			
RPN1	1.67559765	26			
ATP1A1	1.674414136	20			
RELL1	1.673886168	4			
ALB	1.673369011	21			
EIF4A1	1.672700447	37			
SRRT	1.672111914	2			

PFKP	1.67178824	12			
TRIM11	1.667880347	4			
PI4K2B	1.666528186	7			
MDH1	1.66624741	24			
NID1	1.664626881	2			
CA3	1.662227743	10			
TREX1	1.661694264	7			
DHRS4	1.660268014	3			
MAP3K20	1.660124206	8			
CKM	1.6593944	27			
LUM	1.657035811	9			
ATG7	1.654145861	2			
RNF19A	1.653816676	4			
DIABLO	1.646335371	4			
RPS5	1.644722009	11			
SNX6	1.643253301	7			
SARS1	1.64302493	7			
RUFY1	1.639451407	3			
ATP5F1D	1.638440411	5			
LRRC8A	1.636798042	6			
EIF4G2	1.634038171	2			
HSP90AA1	1.631705316	33			
ACADL	1.631370821	3			
IDI1	1.627902194	11			
ABHD16A	1.625809175	2			
RPN2	1.623892685	14			
OSBP2	1.623690182	1			
VASP	1.622602866	11			
SRPRB	1.62164356	23			
HBB	1.620831166	38			
KIFC1	1.61791194	4			
MRPL16	1.613823635	12			
BZW1	1.612967754	17			
OGN	1.611170133	5			
XRN2	1.610481204	8			
DYNLL2	1.60842755	12			
MYH1	1.606107364	37			
HADH	1.603391635	3			
PPA1	1.602829167	11			
SERBP1	1.600562637	13			
CRYAA	1.598825046	6			
ETFDH	1.596583762	4			
MYOZ2	1.595972024	4			
HSD17B11	1.595187256	15			
ARF5	1.586133279	27			
AP3M1	1.586103289	8			
TNS4	1.585337553	14			
SRPRA	1.580760566	5			
LDB3	1.580750167	6			
OSBPL9	1.57953068	1			
COPG1	1.578330304	12			
EIF3B	1.575053273	9			

ATP1B3	1.572449605	13			
PTPN11	1.565578489	4			
WDR1	1.564065085	19			
CFL1	1.56345387	46			
HPR	1.562556228	3			
ACSL3	1.562178928	15			
ISG15	1.560759778	29			
MAP2K1	1.560284285	12			
ACTN2	1.554310386	31			
FKBP9	1.550959925	4			
SND1	1.549482227	9			
RFC2	1.547773055	6			
NFKB1	1.541027984	2			
CRAT	1.539628266	1			
UQCRC1	1.539084936	14			
MYH7	1.538839828	34			
TTN	1.538451064	6			
ADSS2	1.536398053	2			
SLIRP	1.536255048	19			
NAP1L4	1.536217819	15			
NFKB2	1.532998496	3			
ERAP1	1.532980312	2			
SUN2	1.531484927	3			
CCT6A	1.530656841	21			
ARL1	1.528555237	11			
STT3A	1.523772238	8			
CARS1	1.522335939	3			
VTA1	1.518428492	6			
CDK1	1.517927358	26			
HSPA8	1.515609278	49			
TPI1	1.51045459	63			
GFPT1	1.510228522	14			
HSPE1	1.5101058	20			
MARS1	1.507521376	8			
SEC11A	1.506161019	4			
PKM	1.506061708	45			
YWHAQ	1.504496954	40			
PARP12	1.503631732	5			
RRP12	1.502221166	1			
HLA-C	1.498180615	26			
ACSL6	1.497278901	2			
COL6A3	1.496999218	1			
TES	1.494527692	15			
SDR16C5	1.48910387	3			
ACP1	1.486973647	15			
XPO7	1.485979993	3			
ACTC1	1.483679018	47			
PKP2	1.483384263	6			
FASN	1.481004999	4			
PFN2	1.477198576	19			
COX6C	1.475656601	11			
SEC24C	1.473563323	3			

SEC31A	1.471834286	2			
DCTN2	1.4704174	6			
GRAMD1A	1.469807147	2			
SEC61B	1.463166302	10			
OXSR1	1.462947468	11			
MRPS7	1.462922283	14			
IDH3A	1.462915941	18			
TBC1D10A	1.461634089	2			
PSMD2	1.45959812	19			
CAPG	1.458961513	14			
CRYAB	1.456137767	21			
MYOM2	1.453895474	3			
TNNC1	1.446716738	16			
TGFBR2	1.44367122	3			
FERMT2	1.44261691	5			
TMED10	1.44086179	4			
SCD	1.440280316	4			
ACSL1	1.43846668	5			
DNHD1	1.433587973	0			
LPCAT1	1.431439481	7			
GSTM5	1.427601833	8			
NIT2	1.427577294	5			
RAB35	1.426575776	22			
RRAS	1.425322088	18			
FBXO45	1.422541555	7			
PPP2R2A	1.419827538	8			
KDELR2	1.419214281	5			
GNL2	1.415969485	4			
DDRGK1	1.414981145	4			
CASP1	1.414742022	2			
PARK7	1.413967911	30			
ZYX	1.413173621	15			
PSME2	1.411436739	8			
PRDX6	1.408790409	31			
MYBPC2	1.406607975	4			
TRAP1	1.40502434	7			
SNCG	1.40317516	12			
UBE2I	1.401250677	12			
DTX3L	1.400768259	17			
AP1M1	1.395555185	7			
TRAPPC4	1.395534665	4			
G6PD	1.394829994	17			
MRI1	1.393988285	6			
MAPK1	1.392757551	8			
ATP1B1	1.391891003	9			
RTN4	1.390480147	2			
FGB	1.390284906	2			
DDX60L	1.389915192	1			
TIPRL	1.387960548	7			
COX7C	1.387507285	14			
ACYP2	1.384746093	9			
SYNPO2	1.380837933	1			

PATL1	1.380537022	3			
ACTN3	1.378540295	25			
CHCHD3	1.376380243	10			
BUB3	1.374956993	19			
KYNU	1.372703876	17			
SRM	1.372559004	7			
RAP1GDS1	1.366070472	6			
NEB	1.365996777	6			
RETREG3	1.363037725	10			
TNPO1	1.358359106	9			
TXNRD1	1.35427849	10			
MYL3	1.353212355	39			
VANGL1	1.352592316	2			
DENR	1.352405337	4			
SKIC3	1.351730247	1			
SLC25A3	1.348580599	19			
NUTF2	1.347070535	6			
MTREX	1.346665888	3			
RAN	1.346202551	31			
ADK	1.3414314	6			
BAG3	1.341079891	9			
TFG	1.340724352	3			
PLOD1	1.340398704	6			
HSP90AB1	1.340210399	37			
HARS1	1.340024186	6			
EEF1B2	1.337143064	16			
SLC16A3	1.335143634	13			
NDUFA9	1.3337901	10			
OAS3	1.333412853	12			
RAB14	1.330631645	35			
NHERF2	1.330308776	4			
ECHS1	1.327872502	10			
DHCR7	1.327748481	14			
TMEM185B	1.327211434	7			
MTCH1	1.326753608	3			
STX4	1.326338441	13			
APRT	1.324162522	10			
GART	1.318848555	8			
EEF1G	1.318250289	25			
RAP1B	1.316953477	18			
PFDN6	1.316221954	6			
PRSS3	1.315501646	4			
LGALS1	1.314593034	21			
SERPINB6	1.312777449	14			
TYMP	1.308220695	3			
COPS3	1.30687679	3			
MTHFD1	1.306743779	18			
TOLLIP	1.306568368	13			
MYL11	1.306331148	44			
PAK2	1.306224953	9			
FGFR3	1.30595078	2			
NQO1	1.304486794	25			

RNH1	1.302197116	32			
YARS1	1.299724174	15			
SRL	1.296838681	6			
VAT1	1.295187644	10			
SERPINB3	1.293780858	4			
ARF4	1.293676319	36			
MDH2	1.291636271	33			
RPS10	1.28304097	24			
NDUFS1	1.282685555	11			
MTHFD1L	1.281265566	4			
RANGAP1	1.27682157	10			
MT-ATP8	1.276541857	13			
PSAP	1.276196973	2			
DSTN	1.274462715	26			
FNIP1	1.272658556	7			
AAAS	1.271997299	2			
FAM168A	1.267770485	18			
IARS1	1.267472398	18			
TNNT3	1.266866226	8			
GNG5	1.265009436	53			
TPM1	1.26498223	41			
HSPA1B	1.261907722	46			
GSPT1	1.261641882	6			
MYOM1	1.259441479	5			
MYH4	1.258993617	31			
RPS29	1.258834885	13			
SOD2	1.258636821	4			
PRKD2	1.254642054	2			
ALG11	1.253201518	3			
API5	1.25221135	17			
EXOSC4	1.250634942	5			
PXN	1.249554182	1			
RPL23	1.249525071	33			
UGP2	1.249407574	8			
SEC24A	1.245954396	1			
NCSTN	1.245646822	4			
MYH13	1.244938988	13			
CDC27	1.235946245	2			
TRIM22	1.232905295	8			
RAB4A	1.231156746	9			
CASQ1	1.230682716	4			
NUDC	1.230238561	21			
MYLK	1.229135622	0			
CDK9	1.226265783	9			
OTX1	1.225823979	5			
PLIN3	1.220859208	21			
ZDHHC9	1.213563403	3			
CNDP2	1.205390888	2			
RCC2	1.200454014	17			
PALLD	1.197999745	1			
NDUFS2	1.196950606	8			
MOB1B	1.196266074	11			

CAP2	1.191829236	5			
PDHA1	1.188976158	16			
ERGIC1	1.180616333	10			
TNNC2	1.179792625	30			
COL6A1	1.177235491	4			
SLC3A2	1.176669982	20			
EHD2	1.176502129	13			
ACTA1	1.176108784	47			
DLAT	1.170938708	6			
GET3	1.169774922	5			
HNRNPC	1.169464732	26			
DNAJB11	1.168867645	12			
DNAJC7	1.163794001	10			
ENO2	1.161629177	15			
PRELP	1.158617643	3			
LDHA	1.158288844	33			
SLC7A5	1.157801193	13			
MAP2K7	1.157630522	2			
NUDCD1	1.153366127	5			
ZC3H12A	1.151212201	1			
VAPA	1.149942358	11			
GSTP1	1.149341752	18			
PSME1	1.148691382	23			
CEP55	1.14435503	2			
NUP160	1.140976049	1			
CAPN2	1.138585593	4			
OPA1	1.138382989	1			
HNRNPD	1.137890085	22			
PYGL	1.136556219	5			
NDUFA4	1.133818718	12			
MTCH2	1.133719441	7			
EEF1AKMT4	1.132721424	5			
ALDOA	1.132718562	56			
GOT1	1.125004343	10			
PYGB	1.123489176	9			
SLC20A1	1.120893083	4			
IDH2	1.119198664	17			
PRDX4	1.11913586	15			
CLIC1	1.117924974	41			
DCUN1D3	1.116607906	4			
ARL3	1.116420181	6			
RPL19	1.115531357	14			
TMEM167A	1.115218835	13			
MAP4K4	1.105607252	1			
HADHA	1.101302523	19			
ME1	1.100547634	5			
CLTC	1.095232974	13			
IDE	1.09408553	1			
CUL4A	1.093275912	5			
MYL1	1.093118388	25			
ARCN1	1.092631806	13			
PAM	1.090976143	1			

RNPEP	1.090504796	8			
ERP29	1.089543704	5			
ABCE1	1.087842818	13			
ESYT2	1.083576108	7			
HRNR	1.082909062	11			
AP2B1	1.077965571	7			
CDK17	1.077058244	10			
RPL37A	1.072869803	28			
FKBP4	1.069427988	23			
PSMD3	1.068759406	16			
PPP2CB	1.065965373	4			
UCK2	1.060969259	13			
PDLIM5	1.060065864	19			
EZR	1.059994853	18			
PRMT5	1.058910298	7			
HSPA5	1.058410566	38			
NOP2	1.054369196	9			
P4HA1	1.053579149	6			
IGLL5	1.052884395	4			
LRRC47	1.049900066	7			
VDAC2	1.049707948	33			
DUSP11	1.049628902	12			
NDUFS5	1.048921412	16			
CYCS	1.045475652	31			
RPS19	1.044491956	48			
RPS16	1.04445867	40			
TK1	1.044319961	9			
TLN1	1.044013745	1			
ADRM1	1.043408159	4			
AHSA1	1.041935974	13			
VDAC3	1.03940271	12			
GCLM	1.037410402	9			
TKT	1.036686872	18			
NARS1	1.035713298	7			
RNMT	1.028088467	25			
COPE	1.022389514	13			
NME7	1.022327687	3			
GOT2	1.021472929	20			
RAP2C	1.020361248	10			
CPNE3	1.019160689	13			
MAGED2	1.016935067	1			
HSD17B4	1.01681625	8			
APEH	1.016417381	4			
ACOT7	1.016108097	7			
HADHB	1.013385881	9			
PRSS23	1.01080965	20			
COPB2	1.001114852	14			
ATP5F1B	1.000176132	34			
CARM1	0.999614965	7			
AHCY	0.999213946	25			
ELP1	0.998531875	1			
HNRNPH3	0.998520445	18			

SLC25A22	0.995425644	14			
OAS1	0.994908638	6			
CCT4	0.992487286	17			
PSMD11	0.984803786	28			
HDLBP	0.984364763	2			
GMPPA	0.980445817	6			
GPS1	0.97924508	10			
PCBP1	0.977858063	29			
TM9SF2	0.975967189	2			
ATP5PO	0.975821906	37			
UBAP2L	0.975011371	9			
SRP68	0.973198197	7			
EIF2A	0.972476655	3			
DRG2	0.971896074	3			
LRRC40	0.970731446	3			
EIF6	0.969503445	17			
RTRAF	0.969026739	53			
PEBP1	0.966642274	27			
NDUFA3	0.966405138	13			
CA2	0.964015867	5			
MYOT	0.962093735	6			
HLA-H	0.961216691	18			
TMEM263	0.959986763	9			
HBA1	0.959636219	43			
WIPI2	0.95874313	2			
SLC25A1	0.957189487	16			
CCT3	0.956421792	27			
RELA	0.955038182	3			
SOAT1	0.954467576	2			
AP2A1	0.953288429	7			
PARP9	0.952019435	6			
LUC7L	0.950866361	11			
PFKM	0.950688558	14			
EEF1D	0.949996357	39			
SLFN5	0.949500519	2			
DUSP23	0.948396513	5			
FEN1	0.948181288	3			
DTYMK	0.945854548	5			
PDHB	0.944261031	6			
S100A9	0.943426148	11			
RABL3	0.943118104	9			
LARS1	0.942735773	6			
HERC4	0.942387141	1			
OLA1	0.938752917	6			
CD70	0.936168708	6			
TECR	0.936074117	19			
SUMO3	0.935644813	12			
CCT7	0.933880993	24			
COPB1	0.933250351	12			
TBC1D5	0.932788362	5			
WARS1	0.92996977	5			
IGF2BP3	0.927792753	35			

AGPAT5	0.92533625	2			
SMAD2	0.92379704	5			
DHX57	0.922182485	1			
STIP1	0.921478827	12			
BIRC2	0.918413462	10			
IGF1R	0.915456761	2			
PSMC6	0.915125102	11			
MGST1	0.914400932	8			
PSMC5	0.912393736	16			
OGT	0.91222588	4			
GTPBP4	0.910635819	9			
ARF3	0.910558441	34			
TRIP4	0.910191079	1			
HNRNPAB	0.909538443	12			
PSMC2	0.902350138	31			
CCT8	0.900121659	35			
HLA-A	0.891026884	22			
UGCG	0.889562749	5			
DCAF7	0.888263301	3			
ATG14	0.888139206	19			
OTUD4	0.884475226	24			
CPSF7	0.883483571	4			
OR51L1	0.879175103	3			
PSMC1	0.877400116	34			
TRAF4	0.875898575	4			
CSRP2	0.873273983	22			
HK2	0.873168202	8			
HM13	0.872318064	5			
IGKV2-40	0.869821661	11			
PC	0.867809095	5			
IQGAP1	0.865392045	4			
QDPR	0.863555399	5			
ETFA	0.862421902	19			
CKB	0.859384191	8			
TPBG	0.857373439	3			
POSTN	0.856981502	1			
TNIP2	0.856358062	2			
RPL7A	0.854837894	44			
EPS15L1	0.854602086	1			
SEC61A1	0.853613907	8			
TBCE	0.853498467	2			
SMC2	0.852222356	2			
GNL1	0.849991251	3			
VDAC1	0.849798483	38			
IPO7	0.849776048	8			
FAF2	0.849673394	11			
ACTR2	0.848827248	7			
GMPS	0.847054646	8			
APP	0.843923321	3			
PSAT1	0.843314862	17			
UBA6	0.838535934	2			
TRIM25	0.836941834	13			

TGFBI	0.835774849	3			
PSMD1	0.834168297	10			
EBP	0.832947915	4			
FUS	0.825171983	14			
AP2M1	0.824009035	9			
SLC25A5	0.820514556	35			
DDX3X	0.819432489	40			
RHOC	0.818303422	24			
MAP3K5	0.817697811	3			
PRDX1	0.817114558	39			
GNB2	0.809336051	25			
LETM1	0.805558707	2			
HSPA4L	0.802077117	6			
IFIT2	0.800818572	19			
CDKN2A	0.799745118	17			
FLNC	0.799734812	9			
TNNI3	0.797931264	5			
PRPS2	0.795699975	4			
XPO1	0.795515195	5			
PAFAH1B3	0.795291474	11			
SLC25A13	0.792988714	14			
FUBP1	0.792451085	11			
MRPS14	0.789842172	10			
BIN1	0.789610803	2			
RPL26L1	0.789244599	36			
CCT5	0.78782339	26			
SSR1	0.787358199	3			
PAICS	0.787271353	18			
CLPX	0.779431025	9			
AKR1C1	0.77212819	16			
RHOT1	0.771650718	1			
DRG1	0.768577572	6			
PSMD14	0.762281859	14			
CPNE1	0.761915594	9			
ZNF106	0.760082584	2			
GDI2	0.751716718	14			
P4HA2	0.751277037	4			
PSMD12	0.74977091	14			
RPSA	0.748859965	29			
CORO1B	0.747083983	6			
DNAJB12	0.745985676	13			
NTPCR	0.743636616	25			
BLVRA	0.741225241	7			
SUCLG1	0.736438901	7			
DAZAP1	0.733714027	4			
ZFP36L2	0.732263972	9			
MYPN	0.729026224	1			
ACADVL	0.724610835	13			
AFG2B	0.719587709	5			
SKIC8	0.718178603	3			
PPP1CB	0.715310135	34			
ZC3HAV1	0.714332396	37			

SSR4	0.71340539	12			
CCT2	0.711480344	18			
DNM1	0.71146088	2			
XDH	0.708627666	1			
CUL2	0.707655818	1			
TF	0.706863973	2			
RPL13	0.702297906	34			
KRT6A	0.69939344	47			
STT3B	0.695278309	3			
EIF2AK2	0.695131704	14			
RDX	0.689459043	8			
SEPTIN9	0.688064589	12			
TRAM1	0.687951554	5			
PYCR1	0.684903496	8			
VKORC1	0.680533009	8			
CYC1	0.68045264	4			
IFI44	0.680187364	22			
SAMHD1	0.679568373	19			
SLC7A11	0.679071221	3			
RPS25	0.677025853	24			
FAM171A1	0.672007853	1			
SNW1	0.667529036	7			
RPL36	0.667199023	30			
SLC25A11	0.665061129	18			
TMEM165	0.665032192	11			
EIF2B2	0.661094698	7			
LRRC59	0.660521487	18			
B4GAT1	0.658974967	2			
RPS3	0.657769966	50			
S100A10	0.654409039	10			
TAGLN	0.650322808	6			
CYB5R3	0.647736505	14			
SDHB	0.643994527	3			
EXOSC6	0.642333629	4			
ATP5F1C	0.641948198	20			
ABCF1	0.64125154	10			
PON1	0.639466841	3			
NAA15	0.633010639	2			
CDKN2AIP	0.627301082	4			
GLG1	0.626615991	2			
RAC3	0.621974199	20			
RFC3	0.617947288	6			
ANXA11	0.613929596	9			
EWSR1	0.613483934	14			
UQCRFS1P1	0.612975122	7			
RPL11	0.612408682	13			
CACYBP	0.606758207	26			
RAB13	0.60447191	25			
RAP2A	0.604159466	10			
AK2	0.597413928	16			
DCD	0.597159144	10			
PRDX2	0.59675154	15			

TAP1	0.594341234	8			
ARRDC1	0.592429638	4			
NNMT	0.590365229	7			
SUCLA2	0.590170447	20			

Supplemental Table 4. List of antibodies used in this study.

Antibodies	Supplier	Catalogue	Host Species	Species activity	Application	Dilution
anti-USP5	Proteintech	10473-1-AP	Rabbit	Hu, Mo	IHC, WB, IP	1:1000(WB), 3ug (IP), 1:600(IHC)
anti-CXCL9	CST	30327	Rabbit	Hu	mIHC	1:100
anti-CD68	ZSGB-BIO	ZM-0060	Mouse	Hu	mIHC	
anti-CD8	Abcam	ab4055	Rabbit	Hu	mIHC	1:800
anti-GZMB	ZSGB-BIO	TA505931	Mouse	Hu	mIHC	
anti-Cxcl9	Abcam	ab320827	Rabbit	Mo	mIHC	
anti-CD11b	Abcam	ab133357	Rabbit	Mo	mIHC	1:4000
anti-P-STAT2	CST	88410	Rabbit	Hu	WB, IF	1:1000(WB), 1:50(IF)
anti-STAT2	CST	72604	Rabbit	Hu, Mo	WB	1:1000
anti-STAT2	Proteintech	16674-1-AP	Rabbit	Hu	IP	3ug for IP
anti-P-STAT1	CST	9167	Rabbit	Hu	WB	1:1000
anti-STAT1	Proteintech	10144-2-AP	Rabbit	Hu	WB	1:1000
anti-TSG101	Abcam	ab125011	Rabbit	Hu	WB	1:1000
anti-CD81	Proteintech	66866-1-IG	Mouse	Hu	WB	1:1000
anti-Tubulin	Proteintech	66031-1-Ig	Mouse	Hu, Mo	WB	1:1000
anti-FLAG	Sigma	F3165	Mouse		WB, IP	1:1000(WB), 3ug (IP)
anti-FLAG	CST	14793	Rabbit	Hu	WB	1:1000
anti-IRF9	Proteintch	14167-1-AP	Rabbit	Hu	WB, IP	1:1000(WB), 3ug (IP)
anti-Lamin B1	Proteintech	12987-1-AP	Rabbit	Hu	WB	1:1000
anti-LC3B	CST	83506	Mouse	Hu	WB, IP, IF	1:1000(WB), 3ug (IP), 1:100(IF)
anti-HA	Sigma	H6908	Rabbit	Hu	WB	1:1000(WB)
anti-HA	Sigma	H9658	Mouse	Hu	WB	1:1000(WB)
anti-MYC	Proteintech	60003-2-Ig	Mouse	Hu	WB, IP	1:1000(WB), 3ug (IP)
anti-Giantin	Abcam	ab80864	Rabbit	Hu	WB	1:500
anti-Ifnar1	Abclonal	A18594	Rabbit	Mo	WB	1:500
anti-P62	Proteintech	18420-1-AP	Rabbit	Hu	WB	1:1000

anti-ATG5	Abcam	ab108327	Rabbit	Hu	WB	1:1000
anti-ATG9A	Abcam	ab108338	Rabbit	Hu	WB	1:1000
anti-Rabbit IgG	CST	7074	Goat	Rabbit	WB	1:5000
anti-Mouse IgG	CST	7076	Horse	Mouse	WB	1:5000
Alexa Fluor 488	ThermoFisher	A11008	Goat	Rabbit	IF	1:2000
Alexa Fluor 594	ThermoFisher	A11005	Goat	Mouse	IF	1:2000
**Antibodies**	**Supplier**	**Catalogue**	**Clone**	**Species activity**	**Application**	**Dilution**
APC – anti-human HLA-DR	Biolegend	307610	L243	Hu	FC	1:100
PE – anti-human CD86	Biolegend	374206	BU63	Hu	FC	1:100
FITC-anti-mouse CD11b	Biolegend	101205	M1/70	Mo	FC	1:100
BV421-anti-mouse CD11b	Biolegend	101235	M1/70	Mo	FC	1:100
AF700-anti-mouse CD45	Biolegend	103127	30-F11	Mo	FC	1:100
FITC-anti-mouse CD45	Biolegend	103107	30-F11	Mo	FC	1:100
PE-anti-mouse CD45	Biolegend	103105	30-F11	Mo	FC	1:100
APC-anti-mouse CD86	Biolegend	105011	GL-1	Mo	FC	1:100
AF700-anti-mouse CD3	Biolegend	100215	17A2	Mo	FC	1:100
APC-anti-mouse CD3	Biolegend	100311	145-2C11	Mo	FC	1:100
BV605-anti-mouse F4/80	Biolegend	123133	BM8	Mo	FC	1:100
PE-anti-mouse F4/80	Biolegend	123109	BM8	Mo	FC	1:100
PE-Cy7-anti-mouse CD8	Biolegend	100721	53-6.7	Mo	FC	1:100
APC-anti-mouse CD8	Biolegend	100711	53-6.7	Mo	FC	1:100

PE-anti-mouse iNOS	Biolegend	696805	W16030C	Mo	ICFC	1:50
BV421-anti-mouse GZMB	Biolegend	396413	QA18A28	Mo	ICFC	1:50
BV785-anti-mouse TFNα	Biolegend	506341	MP6-XT22	Mo	ICFC	1:50
FITC-anti-mouse IFNγ	Biolegend	505805	XMG1.2	Mo	ICFC	1:50
PE-anti-mouse CD40	Biolegend	124609	3/23	Mo	FC	1:100
Human TruStain FcX™ (Fc Receptor Blocking Solution)	Biolegend	422301	NA	Hu	FC	1:50
TruStain FcX™ (anti-mouse CD16/32) Antibody	Biolegend	101320	93	Mo	FC	1:50
InVivoMab anti-mouse IFNAR-1	BioXcell	BE0241	NA	Mouse	in vivo IFNAR-1 blockade	
InVivoMAb mouse IgG1 isotype control	BioXcell	BE0083	NA	Mouse		

Supplemental Table 5. Sequences of the sgRNAs and primers used in the study.

	Sequence (forward: 5’ to 3’)
Human sgRNAs	
sgUSP5-1	GCCTTAGGGACCCGGATCGT
sgUSP5-2	AGAAGGCGCACTCGTCTTTG
sgSTAT2	GGTGCAGCTGATCCTGAAAG
Human shRNA	
shATG5	CCTGAACAGAATCATCCTTAA
shATG9	TGTAGGAGCAGGATGGAAATA
Human siRNA	
siLC3B-F	GAGUGAGCUCAUCAAGAUA(dT)(dT)
siLC3B-R	UAUCUUGAUGAGCUCACUC(dT)(dT)
Mouse sgRNA	
sgUsp5-1	GACGATCCGTGTCCCCAAGG
sgUsp5-2	CGCACTCGTCTTTATGGACC
sgIfnar1-1	GCTCGCTGTCGTGGGCGCGG
sgStat2-1	CCTGGACCAACTGAACCAGT
Primers used for RT–qPCR	
GAPDH-F	AACGGATTTGGTCGTATTGG
GAPDH-R	TTGATTTTGGAGGGATCTCG
ISG15-F	CGCAGATCACCCAGAAGATCG
ISG15-R	TTCGTCGCATTTGTCCACCA
IFI44-F	TTTTCGATGCGAAGATTCACTGG
IFI44-R	CCTGATGCGTTACATGCCCTT
CCL5-F	CCTGCTGCTTTGCCTACATTGC
CCL5-R	ACACACTTGGCGGTTCTTTCGG
CXCL9-F	CCAGTAGTGAGAAAGGGTCGC
CXCL9-R	AGGGCTTGGGGCAAATTGTT
CXCL10-F	GGTGAGAAGAGATGTCTGAATCC
CXCL10-R	GTCCATCCTTGGAAGCACTGCA
HLA-A-F	AGATACACCTGCCATGTGCAGC
HLA-A-R	GATCACAGCTCCAAGGAGAACC
B2M-F	CCACTGAAAAAGATGAGTATGCCT

B2M-R	CCAATCCAAATGCGGCATCTTCA
TAP1-F	GCAGTCAACTCCTGGACCACTA
TAP1-R	CAAGGTTCCCACTGCTTACAGC
SPP1-F	CTCCATTGACTCGAACGACTC
SPP1-R	CAGGTCTGCGAAACTTCTTAGAT
Gapdh-F	CATCACTGCCACCCAGAAGACTG
Gapdh-R	ATGCCAGTGAGCTTCCCGTTCAG
Cxcl9-F	GGAGTTCGAGGAACCCTAGTG
Cxcl9-R	GGGATTTGTAGTGGATCGTGC
Cxcl10-F	ATCATCCCTGCGAGCCTATCCT
Cxcl10-R	GACCTTTTTTGGCTAAACGCTTTC
