# Supplementary material for: LC3-dependent intercellular transfer of phosphorylated STAT1/2 elicits CXCL9+ macrophages and enhances radiation-induced antitumor immunity
Source: J Clin Invest. 2025 Dec 1;135(23):e195279. doi: 10.1172/JCI195279 (PMC12646668; doi:10.1172/JCI195279)

Figure 4F

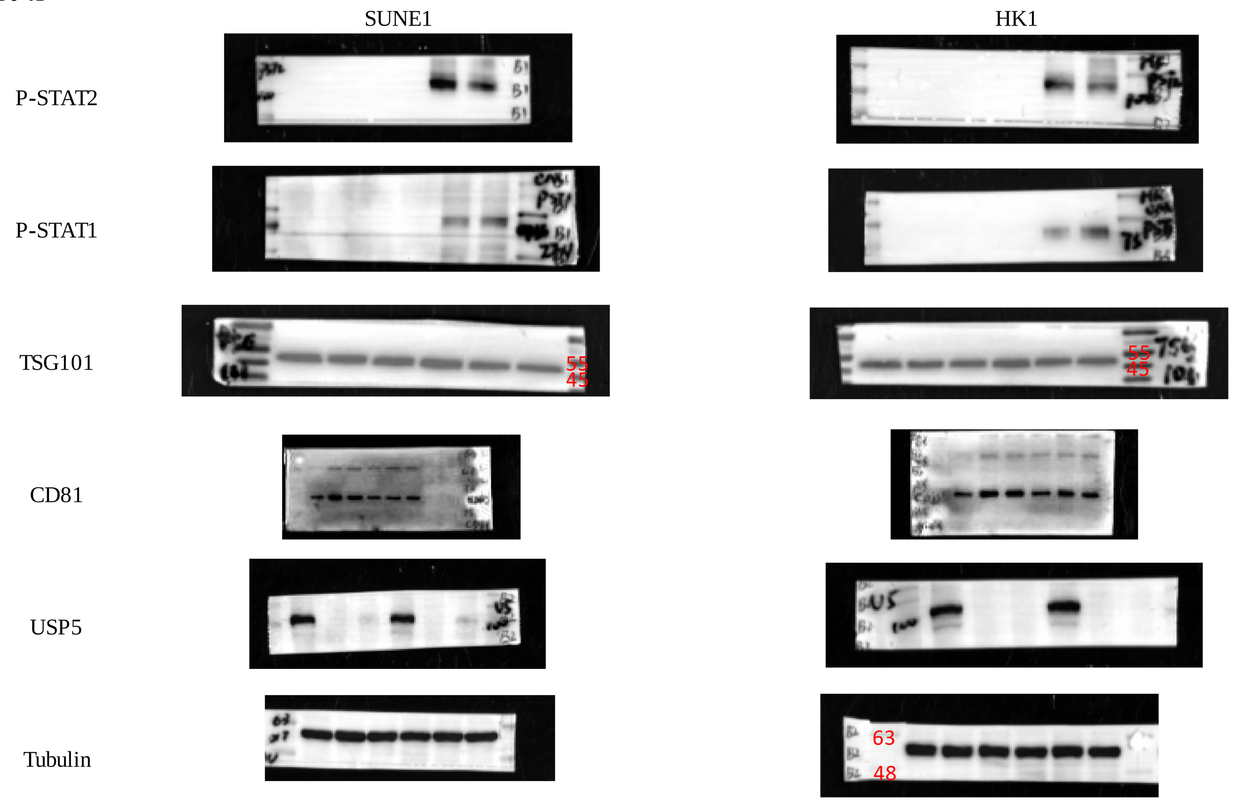

Figure 4G

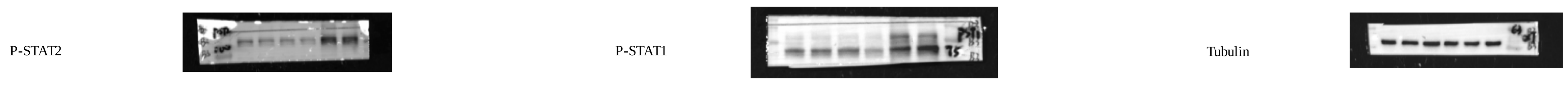

Figure 5A

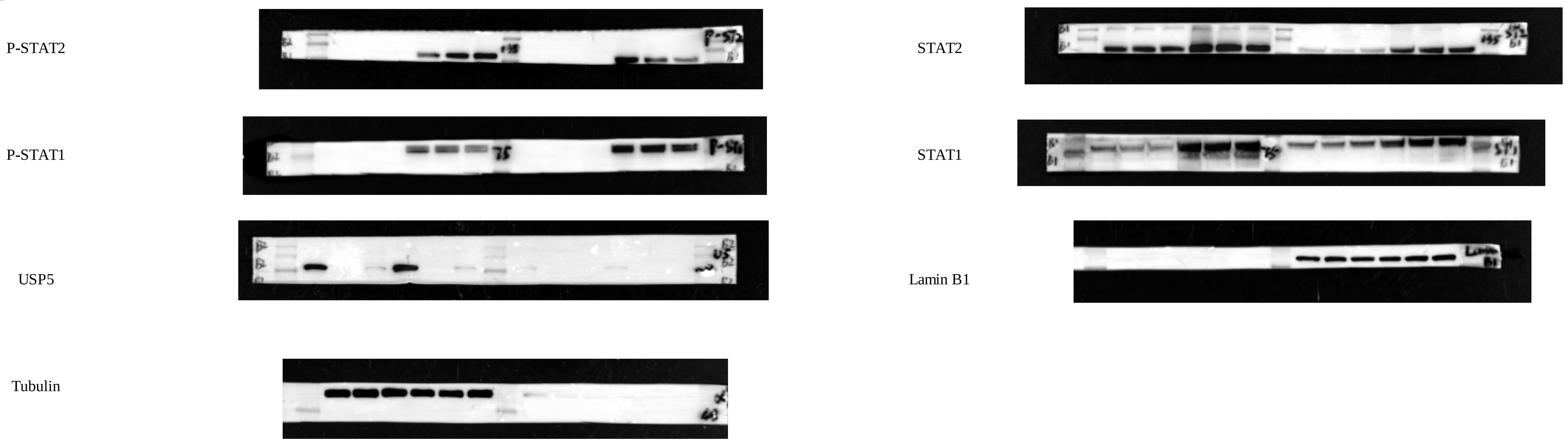

Figure 5E

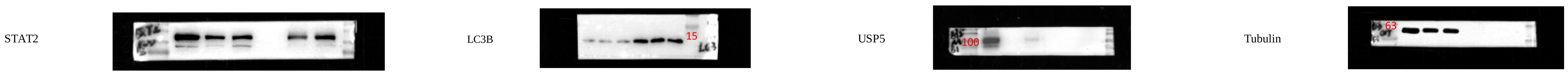

Figure 5F

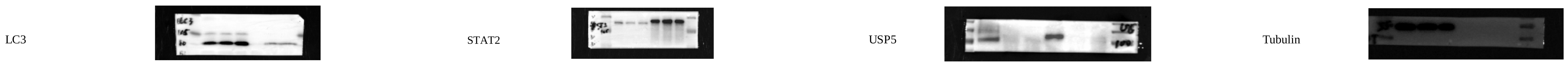

Figure 5I

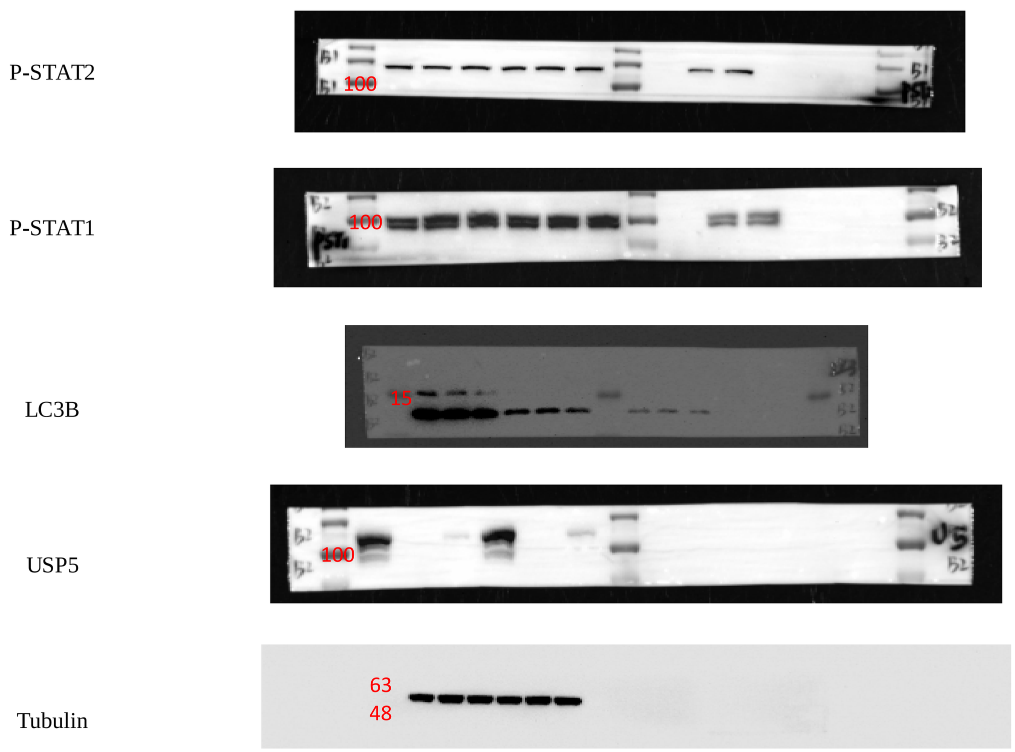

Figure 6A

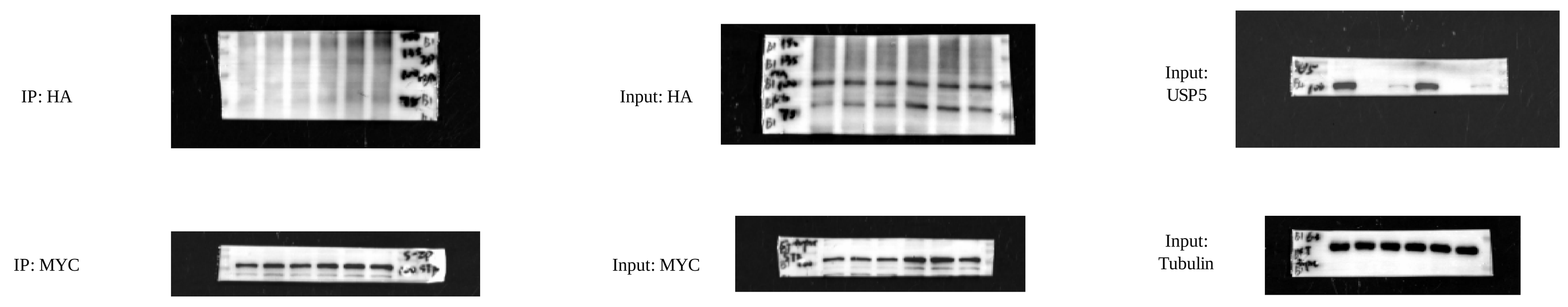

Figure 6B

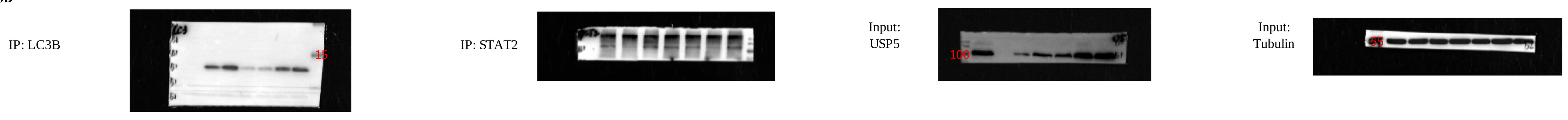

Figure 6C

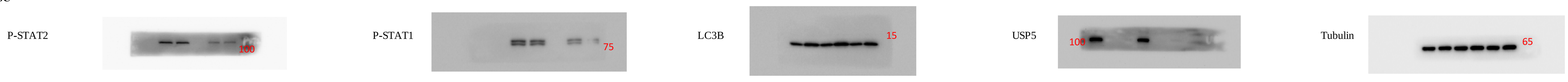

Figure 6D

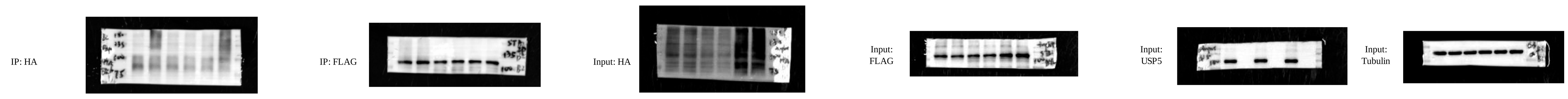

Figure 6F

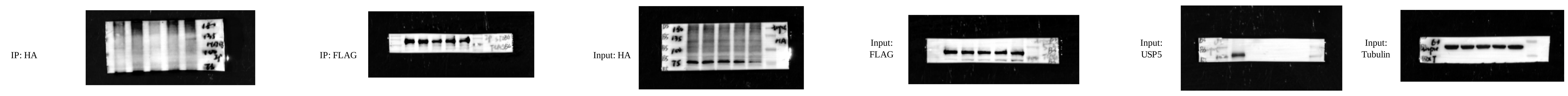

Figure 6J

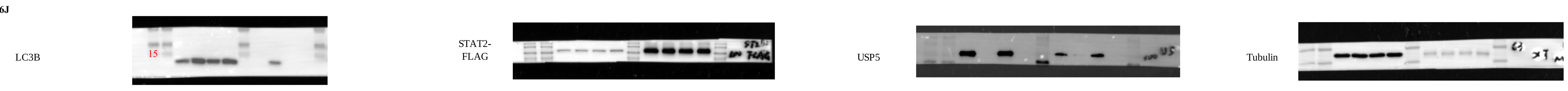

Figure 6K

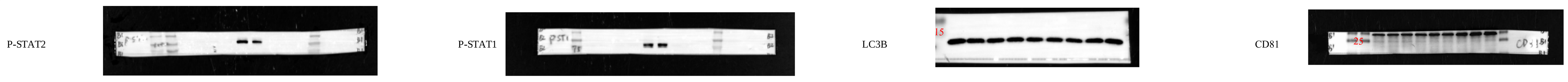

Figure 7A

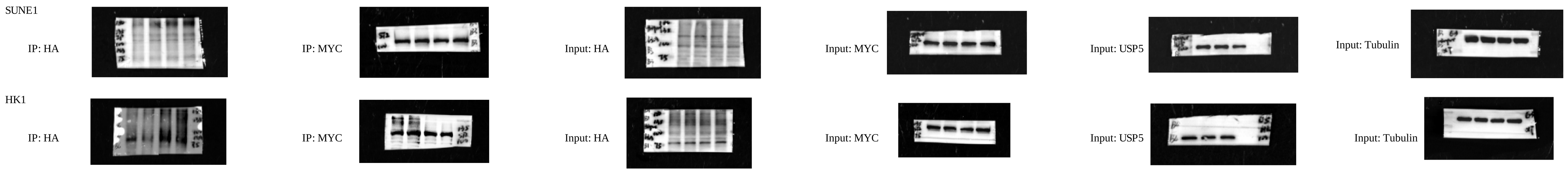

Figure 7B

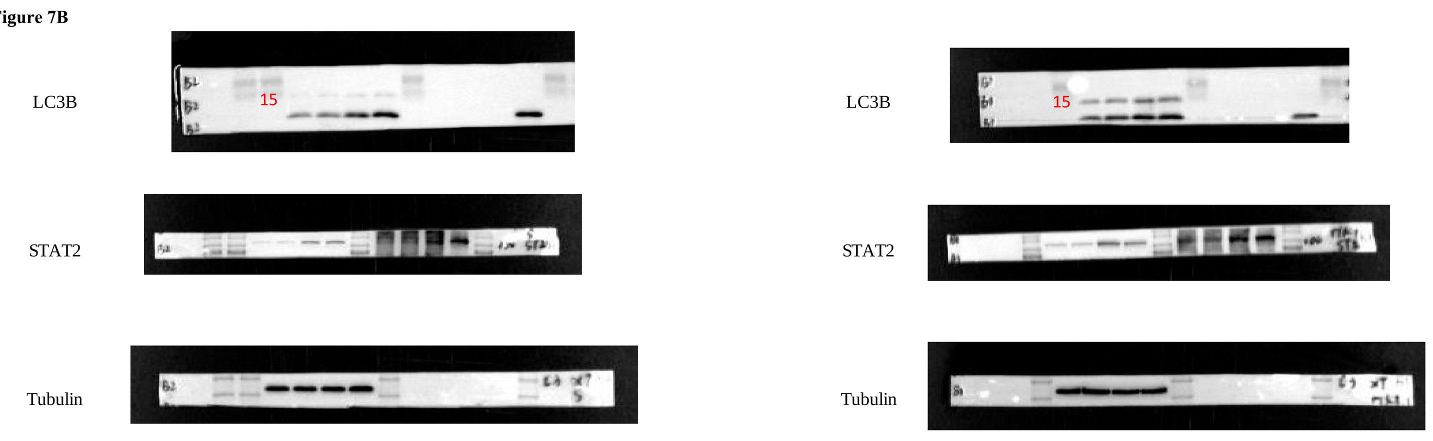

Figure 7D

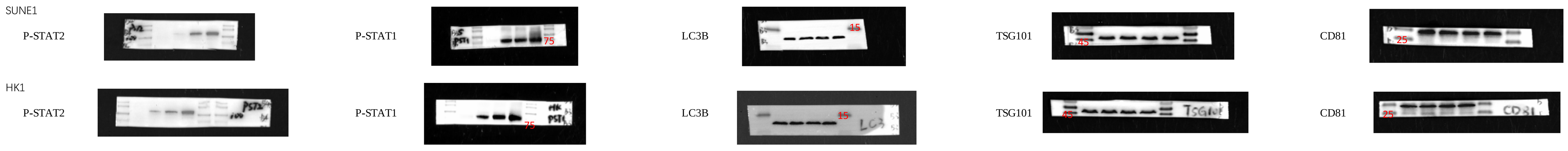

Supplemental Figure 3A

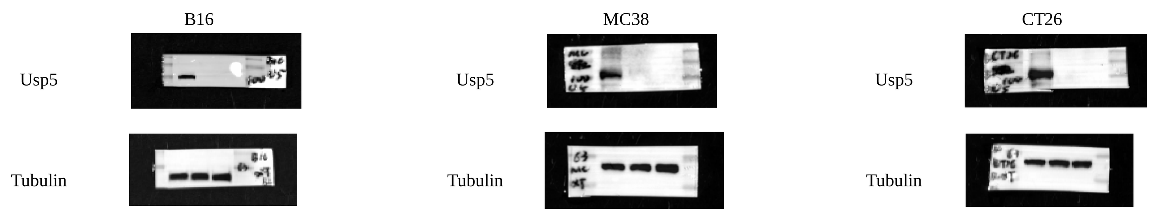

Supplemental Figure 4F

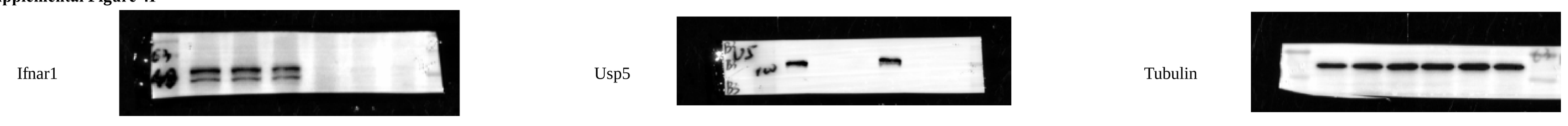

Supplemental Figure 5A

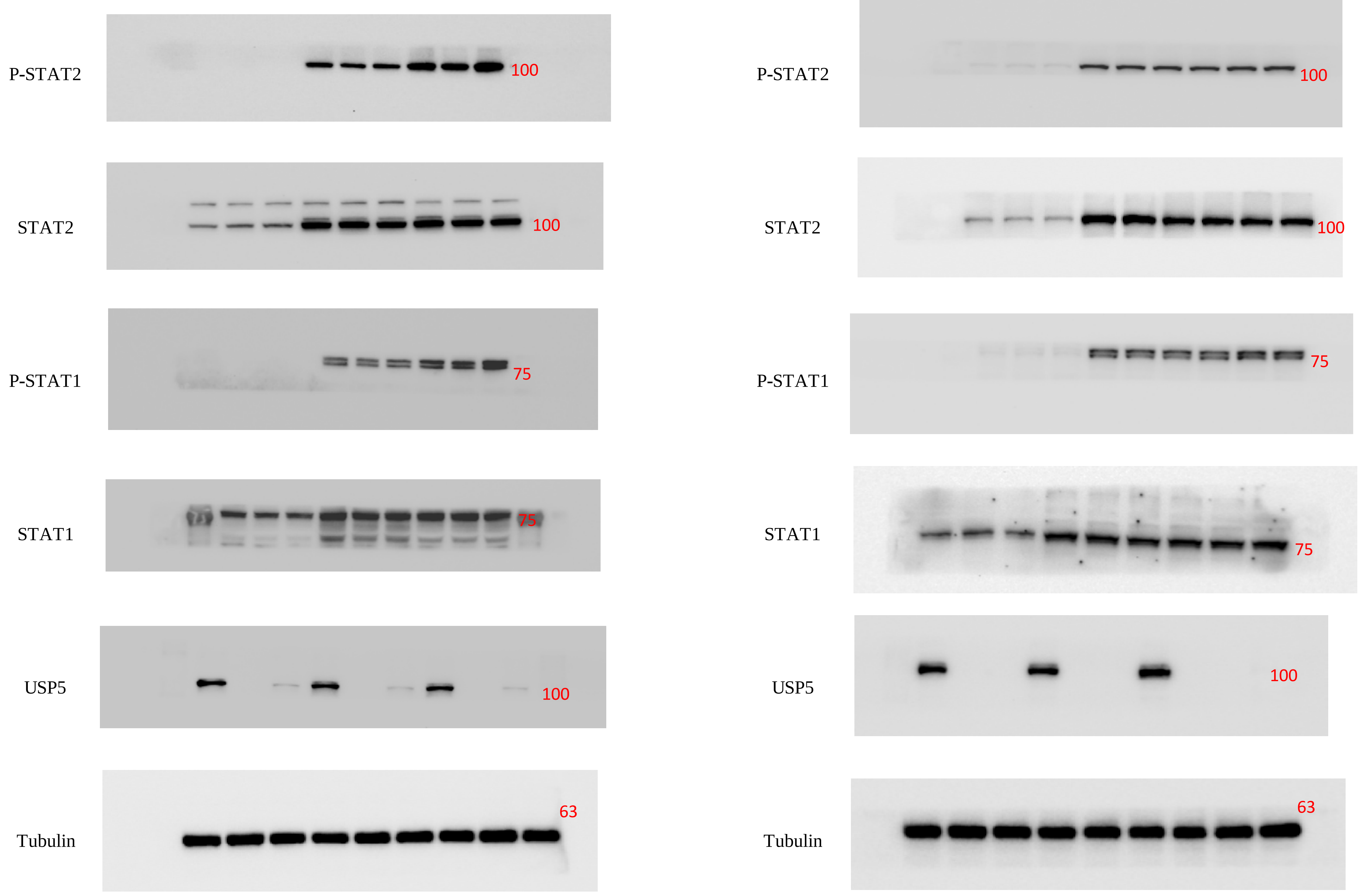

Supplemental Figure 5E

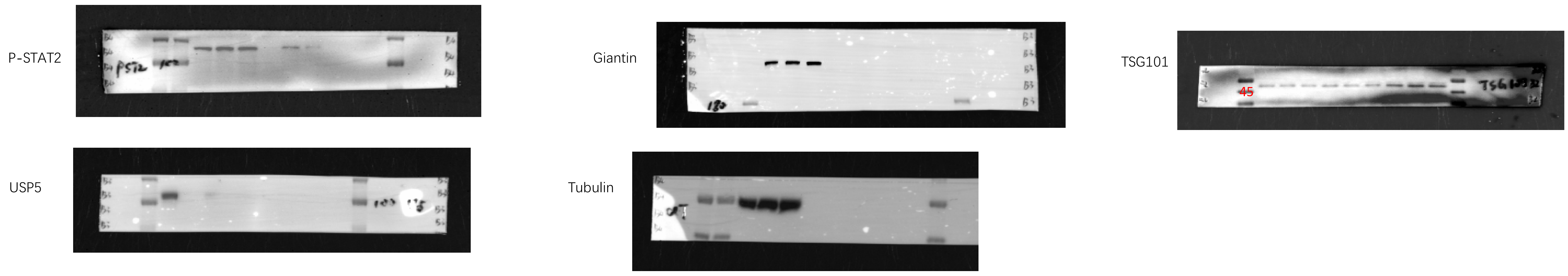

Supplemental Figure 5F

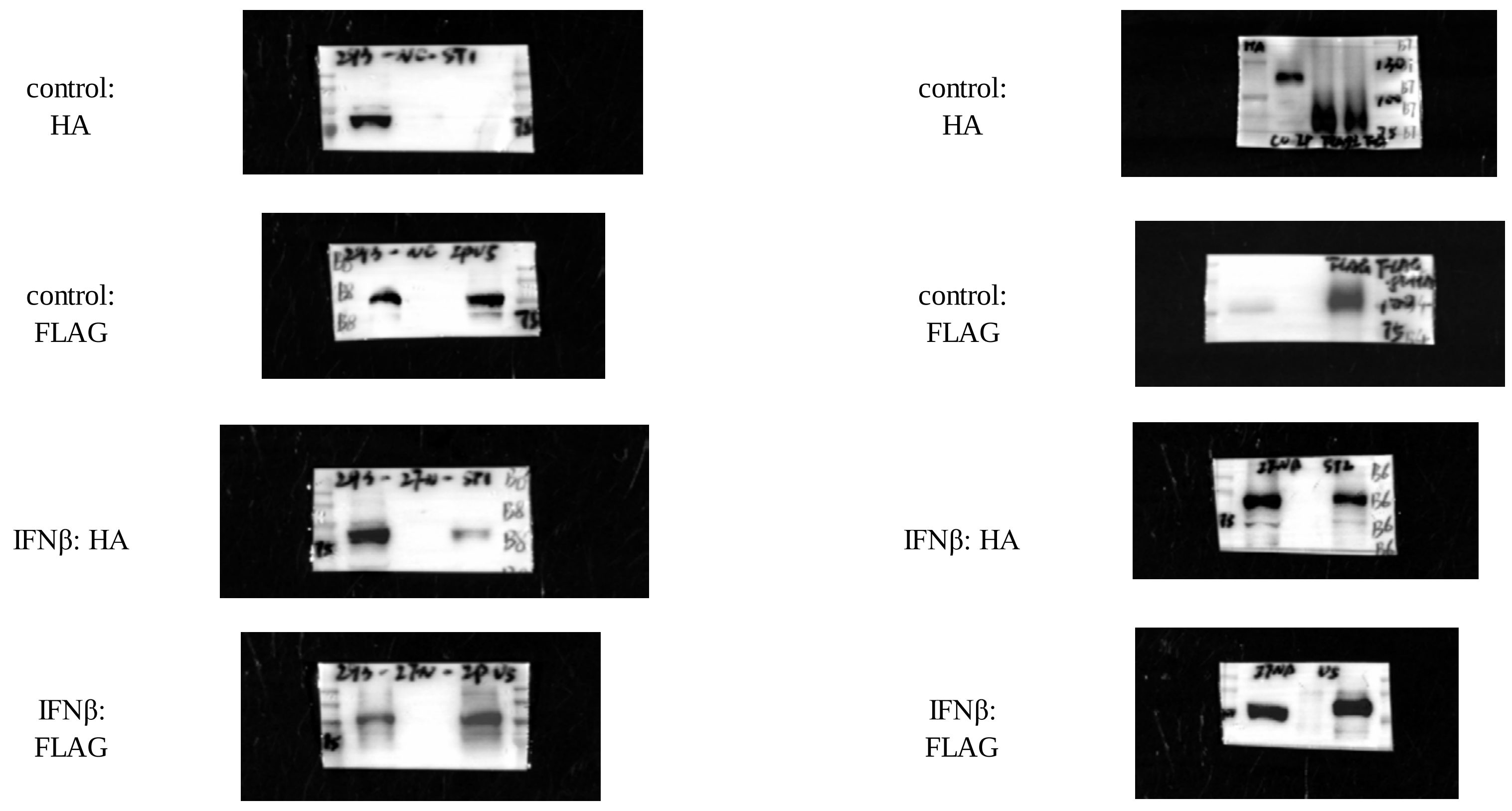

Supplemental Figure 5G

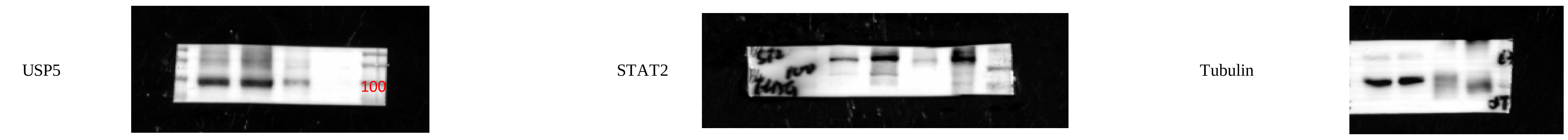

Supplemental Figure 5H

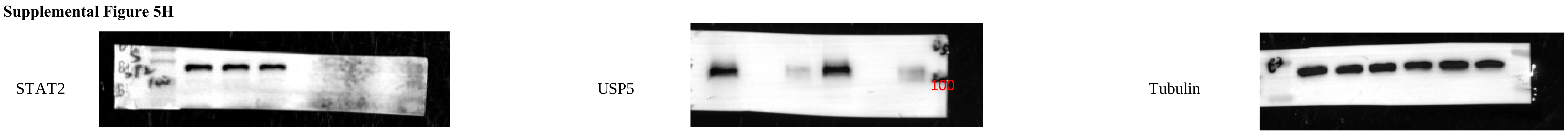

Supplemental Figure 5I

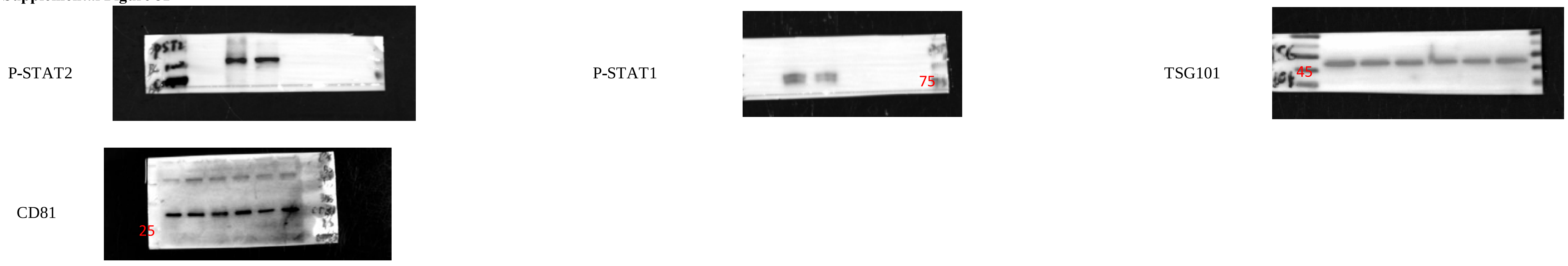

Supplemental Figure 5J

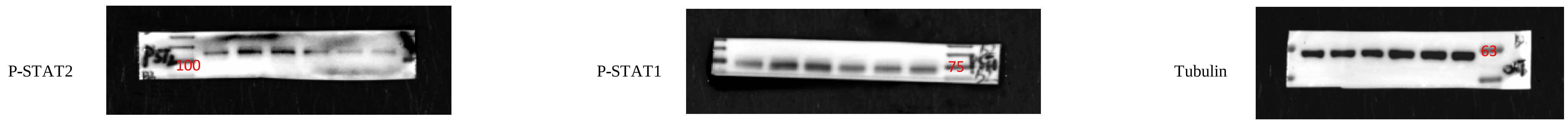

Supplemental Figure 5K

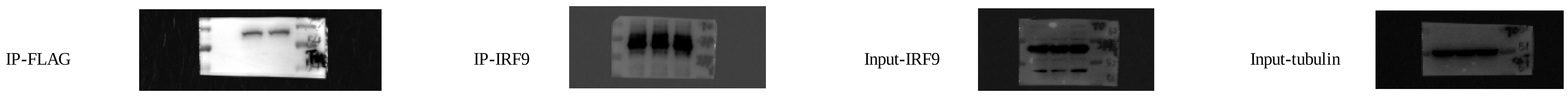

Supplemental Figure 6C

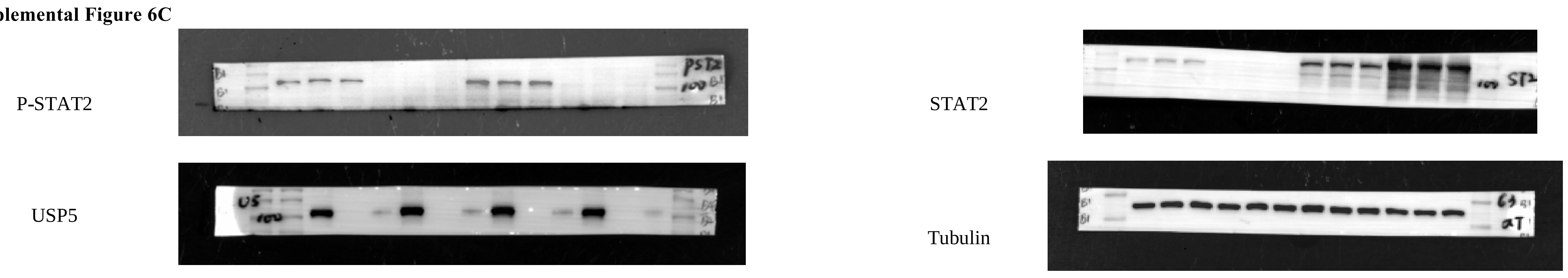

Supplemental Figure 6F

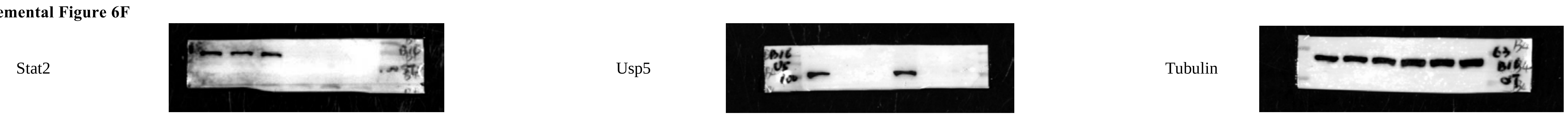

Supplemental Figure 7D

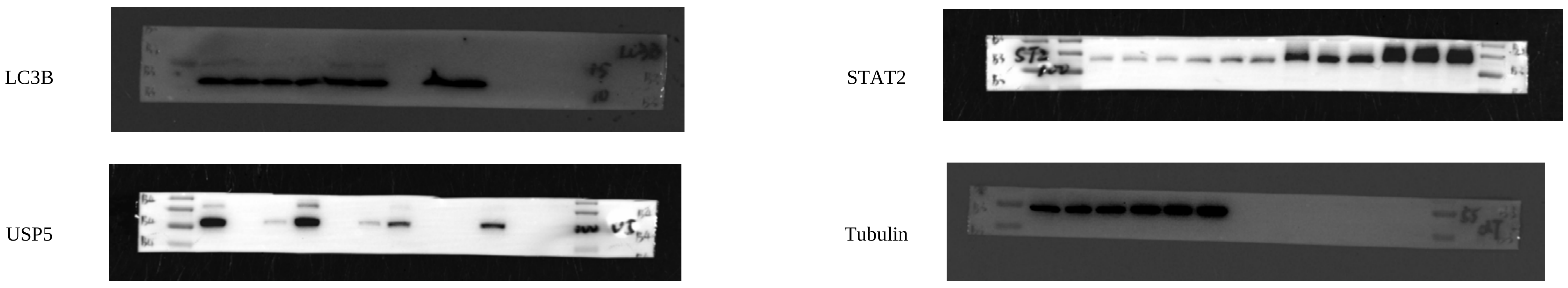

Supplemental Figure 8B

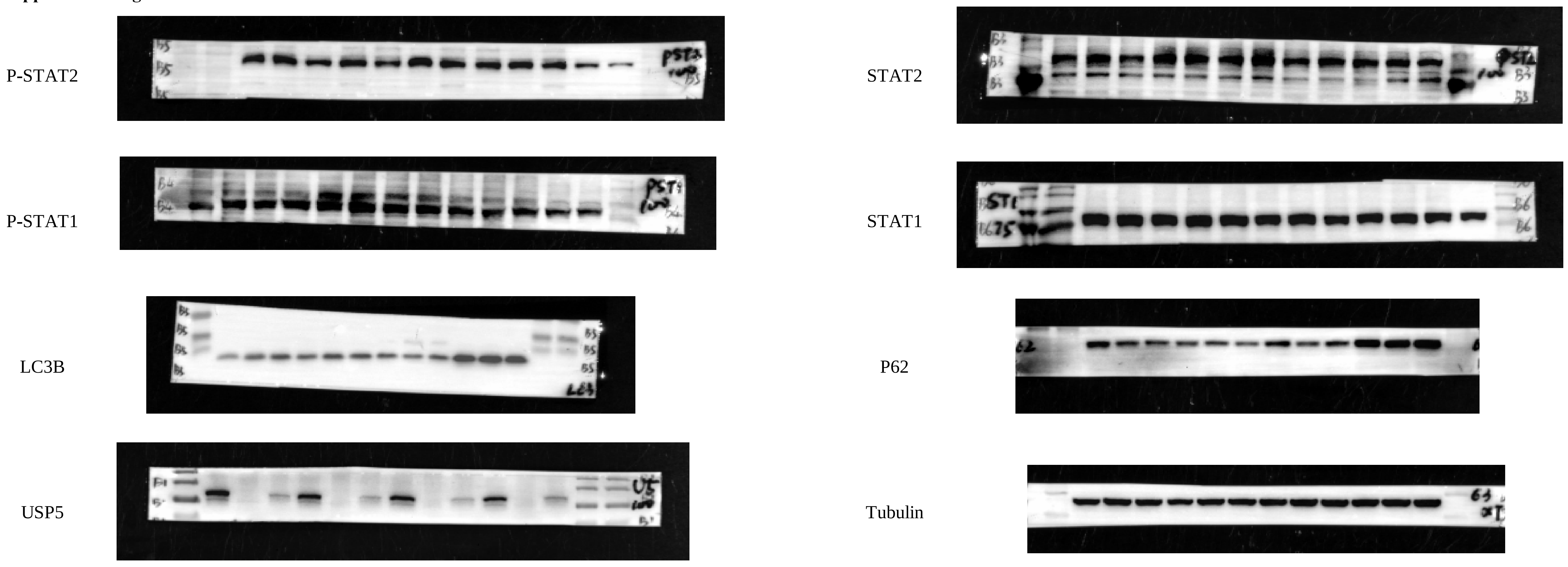

Supplemental Figure 8C

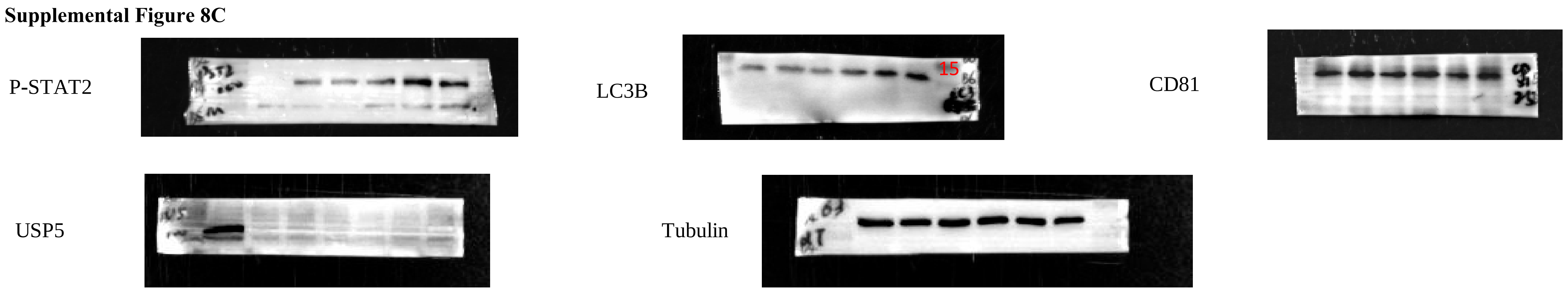

Supplemental Figure 8D

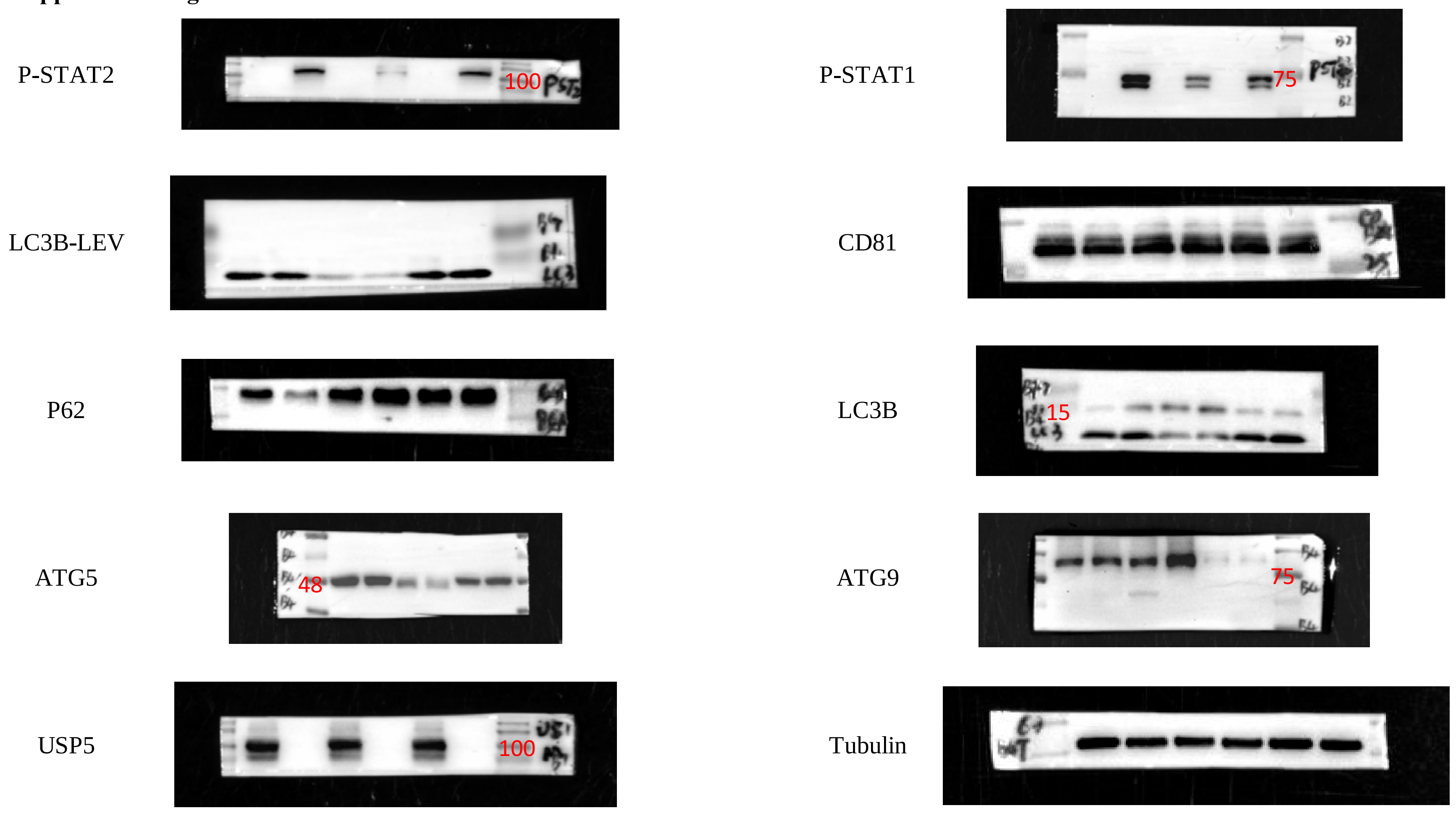

Supplemental Figure 9A

IP: HA

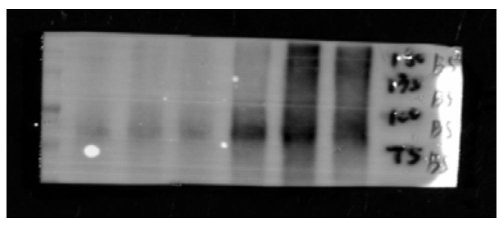

Input: HA

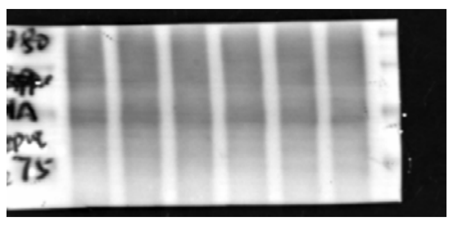

Input: USP5

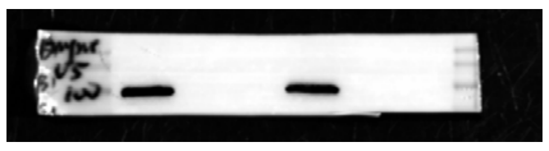

IP: STAT2

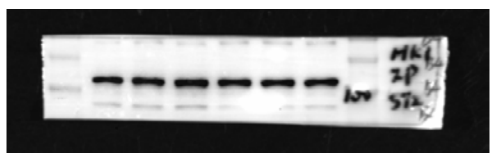

Input: STAT2

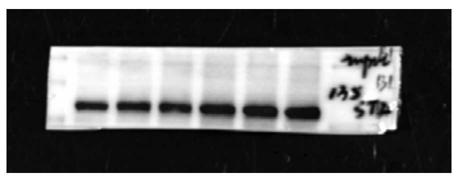

Input: Tubulin

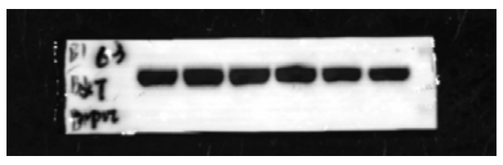

Supplemental Figure 9B

SUNE1

HK1

IP: HA

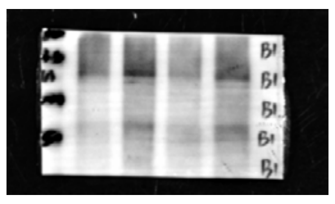

IP: HA

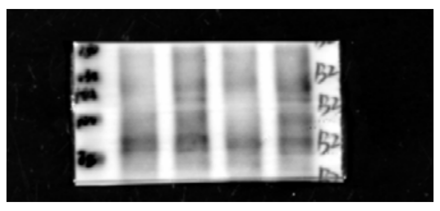

IP:STAT2-MYC

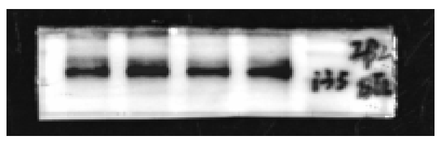

IP:STAT2-MYC

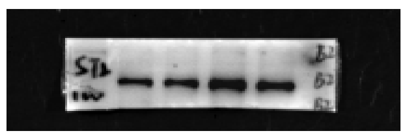

Input: HA

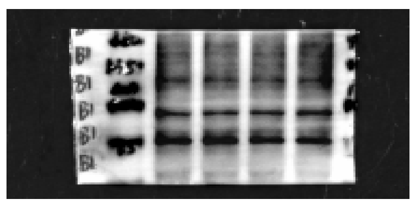

Input: HA

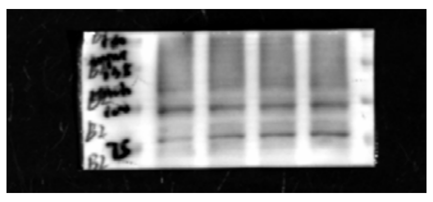

Input: STAT2-MYC

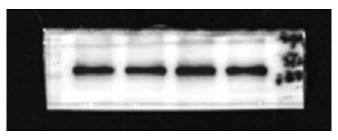

Input: STAT2-

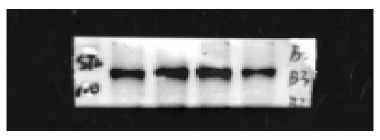

Input: USP5

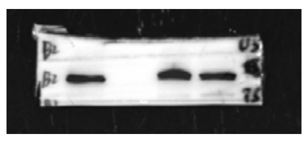

Input: USP5

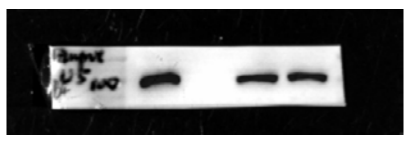

Input: Tubulin

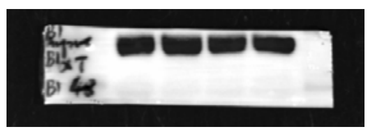

Input: Tubulin

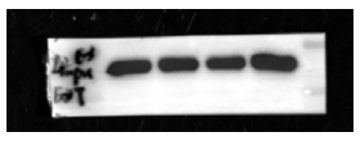

Supplemental Figure 9C

P-STAT2

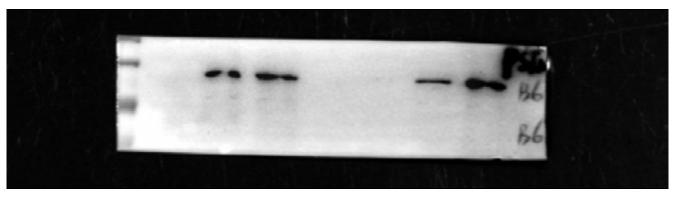

LC3B

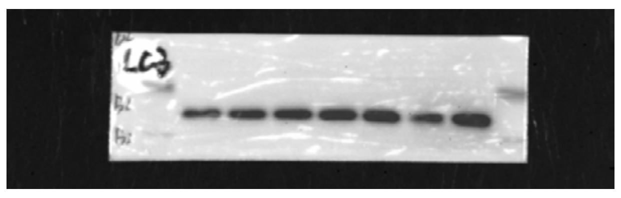

USP5

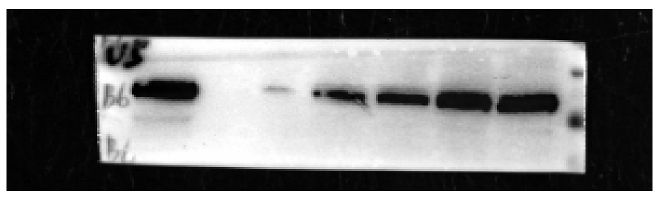

Tubulin

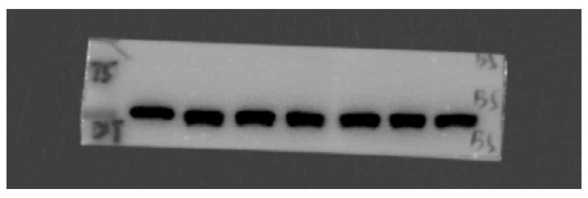

Supplemental Figure 9D

P-STAT2

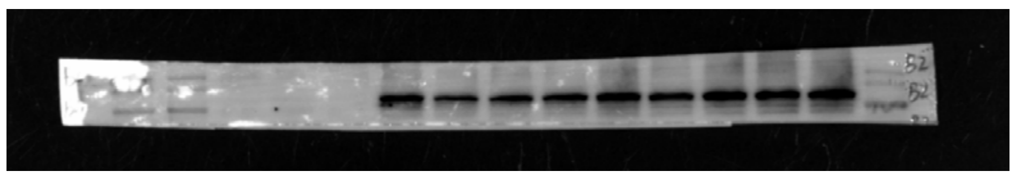

STAT2

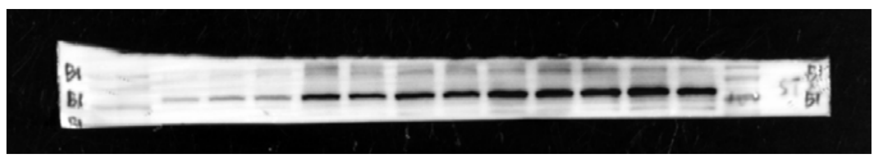

USP5

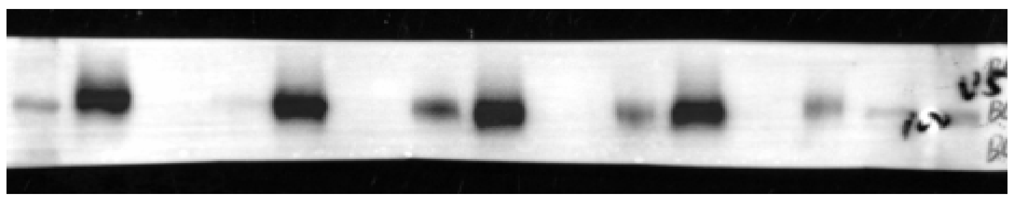

Tubulin

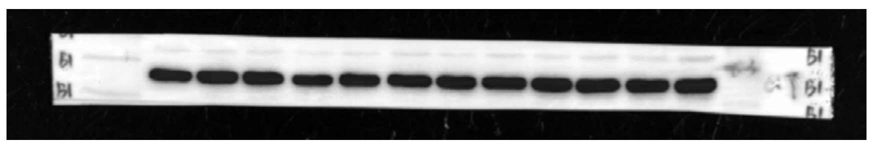

Supplemental Figure 10A

IP: HA

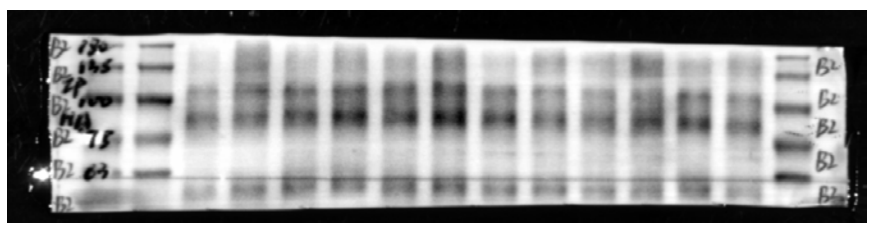

Input: HA

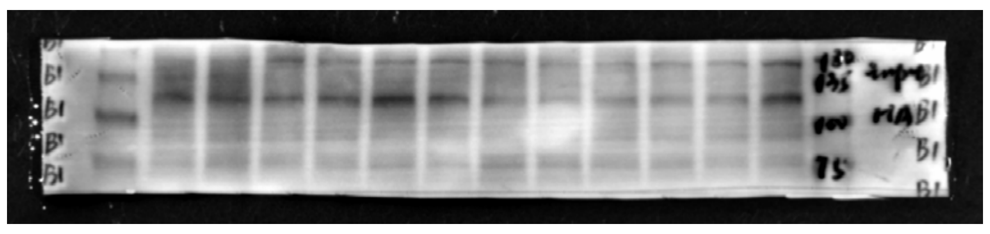

IP: FLAG

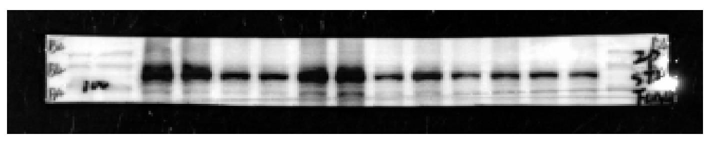

Input: FLAG

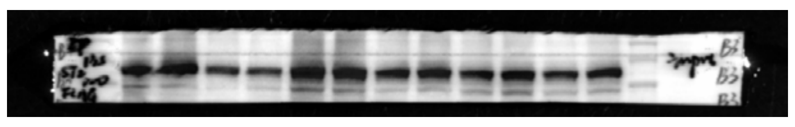

Input: USP5

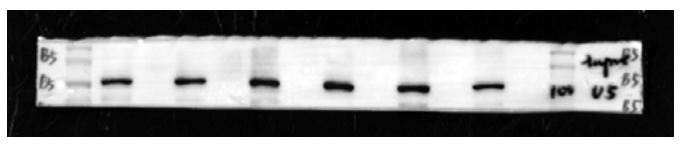

Input: Tubulin

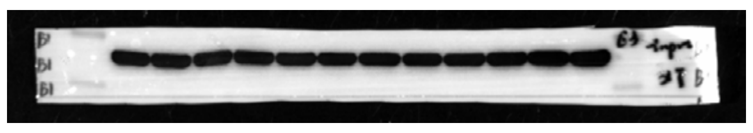

Supplemental Figure 10B

IP: HA

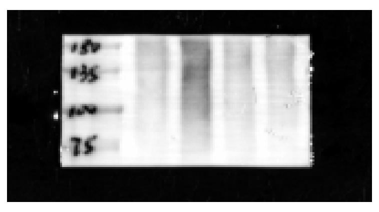

IP: FLAG

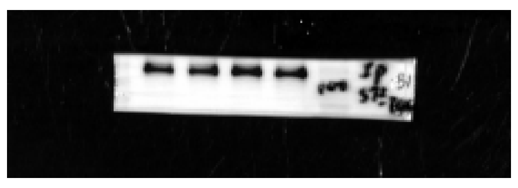

Input: HA

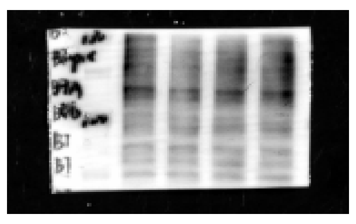

Input: FLAG

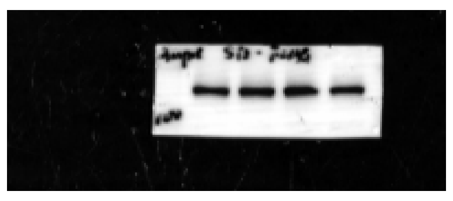

Input: USP5

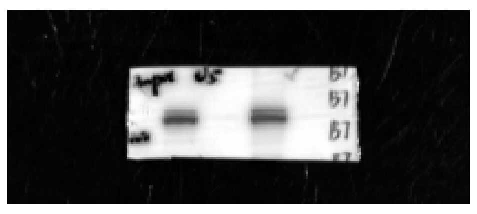

Input: Tubulin

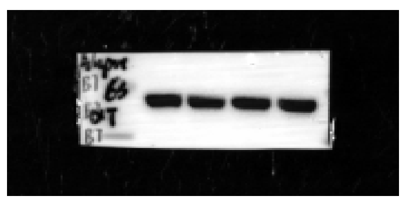

Supplemental Figure 10C

IP: HA

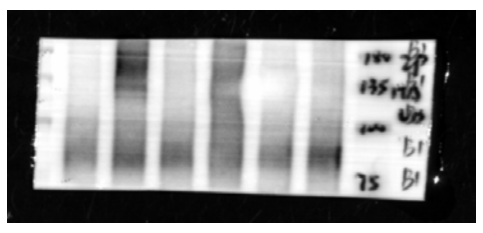

Input: HA

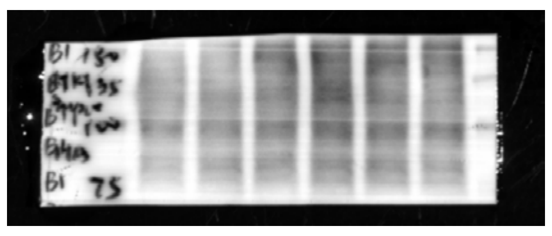

IP: FLAG

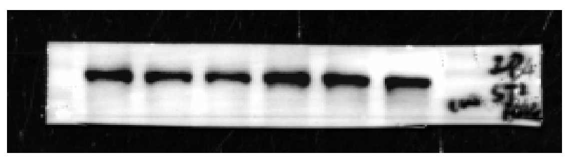

Input: FLAG

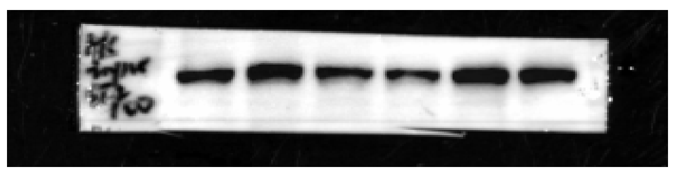

Input: USP5

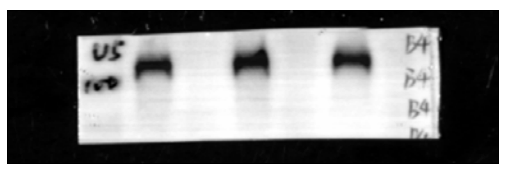

Input: Tubulin

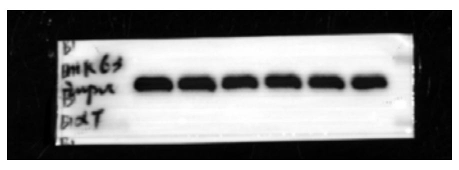

Supplemental Figure 10E

P-STAT2

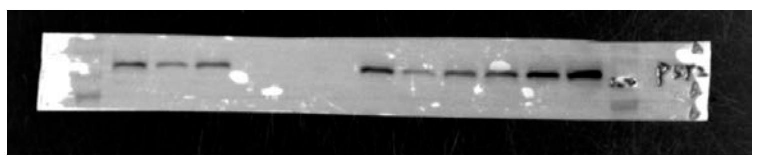

STAT2

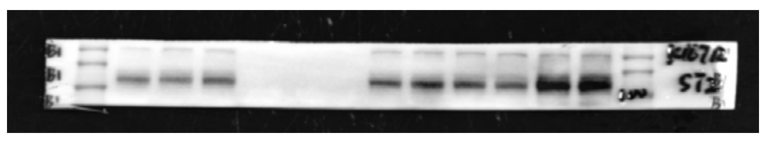

USP5

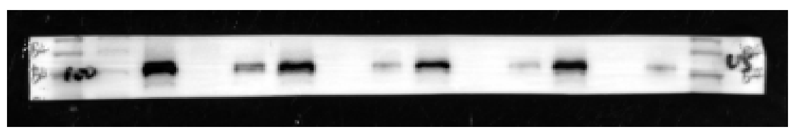

Tubulin

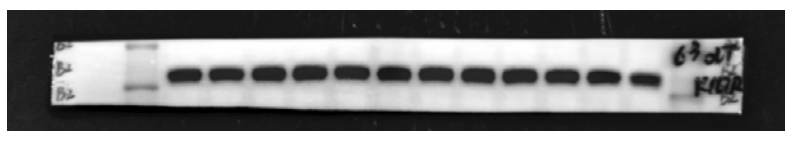

Supplemental Figure 11A

P-STAT2

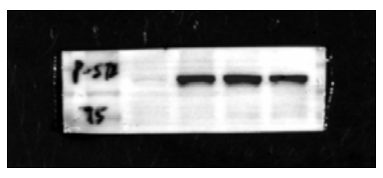

STAT2

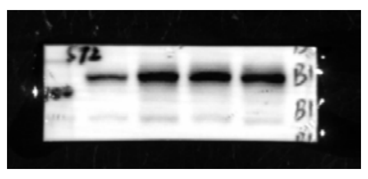

P-STAT1

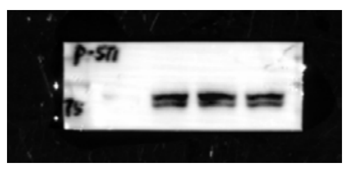

STAT1

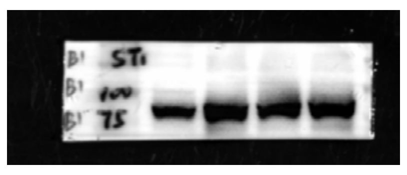

Tubulin

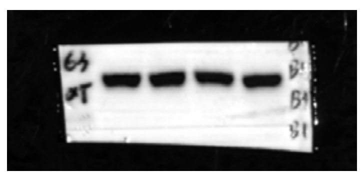

Supplement: Unedited blot and gel images [file jci-135-195279-s127.pdf]
